# Assessment of oligomerization of bacterial micro-compartment shell components with the tripartite GFP reporter technology

**DOI:** 10.1371/journal.pone.0294760

**Published:** 2023-11-27

**Authors:** Lucie Barthe, Vanessa Soldan, Luis F. Garcia-Alles

**Affiliations:** 1 Toulouse Biotechnology Institute, CNRS, INRAE, INSA, Université de Toulouse, Toulouse, France; 2 Plateforme de Microscopie Electronique Intégrative METi, CNRS, Centre de Biologie Intégrative, Toulouse, France; Consejo Superior de Investigaciones Cientificas, SPAIN

## Abstract

Bacterial micro-compartments (BMC) are complex macromolecular assemblies that participate in varied metabolic processes in about 20% of bacterial species. Most of these organisms carry BMC genetic information organized in operons that often include several paralog genes coding for components of the compartment shell. BMC shell constituents can be classified depending on their oligomerization state as hexamers (BMC-H), pentamers (BMC-P) or trimers (BMC-T). Formation of hetero-oligomers combining different protein homologs is theoretically feasible, something that could ultimately modify BMC shell rigidity or permeability, for instance. Despite that, it remains largely unknown whether hetero-oligomerization is a widespread phenomenon. Here, we demonstrated that the tripartite GFP (tGFP) reporter technology is an appropriate tool that might be exploited for such purposes. Thus, after optimizing parameters such as the size of linkers connecting investigated proteins to GFP10 or GFP11 peptides, the type and strength of promoters, or the impact of placing coding cassettes in the same or different plasmids, homo-oligomerization processes could be successfully monitored for any of the three BMC shell classes. Moreover, the screen perfectly reproduced published data on hetero-association between couples of CcmK homologues from *Syn*. *sp*. PCC6803, which were obtained following a different approach. This study paves the way for mid/high throughput screens to characterize the extent of hetero-oligomerization occurrence in BMC-possessing bacteria, and most especially in organisms endowed with several BMC types and carrying numerous shell paralogs. On the other hand, our study also unveiled technology limitations deriving from the low solubility of one of the components of this modified split-GFP approach, the GFP1-9.

## Introduction

Bacterial micro-compartments (BMC) are polyhedral structures present in almost 20% of prokaryotes [1]. They are composed of a semi-permeable protein shell containing a set of enzymes whose nature will define the specialization of the BMC in a given metabolic pathway. In addition to increasing the catalytic efficiency of the process by concentrating the enzymes in a restricted space, BMCs play a barrier role, sequestering in their lumen reaction intermediates that are toxic for the cell or volatile [2]. There exists various BMC among prokaryotic phyla [1, 3], with carboxysomes and the Eut and the Pdu micro-compartments being the best characterized.

Shell proteins can be divided in three classes: those associating as pentamers (BMC-P), which compose the Pfam03319 structural fold and were predicted [4] and shown [5] to occupy vertices of BMC polyhedral shells, and those organizing as hexamers (BMC-H) or as trimers (BMC-T), which assemble in BMC faces and edges. The last two groups are structurally related, BMC-T consisting of two connected BMC-H domains (Pfam00936). Several paralogs coding for monomers from the 3 different classes are often present in a given organism. For instance, an average of 3.5 paralogs were estimated for BMC-H monomers [3].

Bearing in mind BMC usual organization in operons, it is reasonable to consider that hetero-oligomers could form. Two studies demonstrated cross-associations between CcmK3 and CcmK4 monomers from two β-cyanobacteria when the two BMC-H were co-expressed in *E*. *coli*, as well as between CcmK1 and CcmK2 from *Synechocystis sp*. PCC 6803 (*Syn6803*) [6, 7]. However, it is presently unknown whether hetero-oligomerization occurs in the natural host, whether it can be extended to other BMC types such as the Pdu or Eut or to other shell components like BMC-T or BMC-P. It is important to highlight that by breaking subunit symmetry, hetero-oligomerization might modify shell properties like permeability or rigidity.

Diverse technologies are available for the detection of protein/protein interactions (PPI) in cells, and might be appropriate to search for compatibilities between BMC shell homologs *in vivo* with mid/high throughput. None of them offers a perfect coverage of PPIs, and combining multiple approaches is often required [8]. Since proteins targeted in our study belong to well characterized structural families, we opted instead by adapting one of the most suited techniques, the tripartite GFP (tGFP). This technology is based on an interaction-guided reconstitution of a GFP that was split into three portions [9]: GFP β-strands 1 to 9 (GFP1-9), and the two individual β-strands 10 and 11 (*i*.*e*. GFP10 and GFP11) that are fused to the proteins of interest (POI) to be screened. Apart from presenting intrinsic advantages over bipartite split approaches, such as decreased random reconstitution probability, our interest for tGFP was justified by the small size of GFP10 and GFP11, something that should minimize effects on POIs expression and solubility, as well as interferences on PPI.

Here, we demonstrate that tGFP is a suitable technology for the detection of BMC shell protein interactions. Not only BMC-H oligomerization but also associations leading to BMC-T or BMC-P oligomers could be captured correctly. Moreover, the technology corroborated reported conclusions on compatibilities between pairs of CcmK proteins from *Syn6803* that cause formation of hetero-hexamers [6]. This validated approach thus opens an avenue for mid/high throughput investigations intending to unravel the physiological importance and extent of such uncharacterized events.

## Results

### Monitoring BMC-H oligomerization: Design of controls

As model protein for the optimization phases of this study, we selected the BMC-H from the *Rhodococcus* and *Mycobacterium* Microcompartments (RMM) [10]. In our experience, this protein is expressed recombinantly at average levels when compared to other BMC-H. Its N- and C-terminal extensions that protrude from the hexamer core are among the shortest of BMC-H. GFP10 and GFP11 peptides were connected to the C-terminus. Although tagging position was expected to impact signal readouts, as shown in later stages of this work using a N-ter tagged construct, our initial choice was based on the higher tolerance for C-ter modifications demonstrated with CcmK proteins [6].

Apart from targeted PPI that lead to oligomerization, other phenomena might contribute nonspecifically to fluorescence signals, which needed to be gauged. These included the random encounter of partners induced by intracellular protein accumulation during the assay coupled to the expected irreversibility of the fluorescence reconstitution process [11]. The buckwheat trypsin inhibitor (BWI) was selected to account for it. BWI is well expressed and its crystal structure revealed a monomeric state [12]. Though Alphafold2 (AF2) predictions suggested a potential dimerization (S1 Fig and S1 Table), the fact that the last residue of the native protein was fully embedded in the dimer interface guaranteed a monomeric state for the C-ter tagged construct, something indeed confirmed by AF2. Nonetheless, other proteins expected to be monomeric, dimeric or trimeric were included in later phases of the study to assess the tGFP assay robustness (see Supplementary information and S2 Fig). A second probed contribution was the potential GFP reconstitution from tags exposed on aggregated material. For this purpose, we chose CcmK3 from *Syn6803*, in virtue of its high expression and insolubility in *E*.*coli* [13]. Another considered source of fluorescence was the reconstitution implying tags attached to neighbor hexamer subunits in higher order assemblies, something common when BMC-H are over-expressed *in vivo* [14, 15]. Several strategies pursuing the impediment of RMM assembly were applied to quantify such signal input (see below).

### Fluorescence reconstitution is hampered when partners are expressed from different vectors

**Fig 1:**
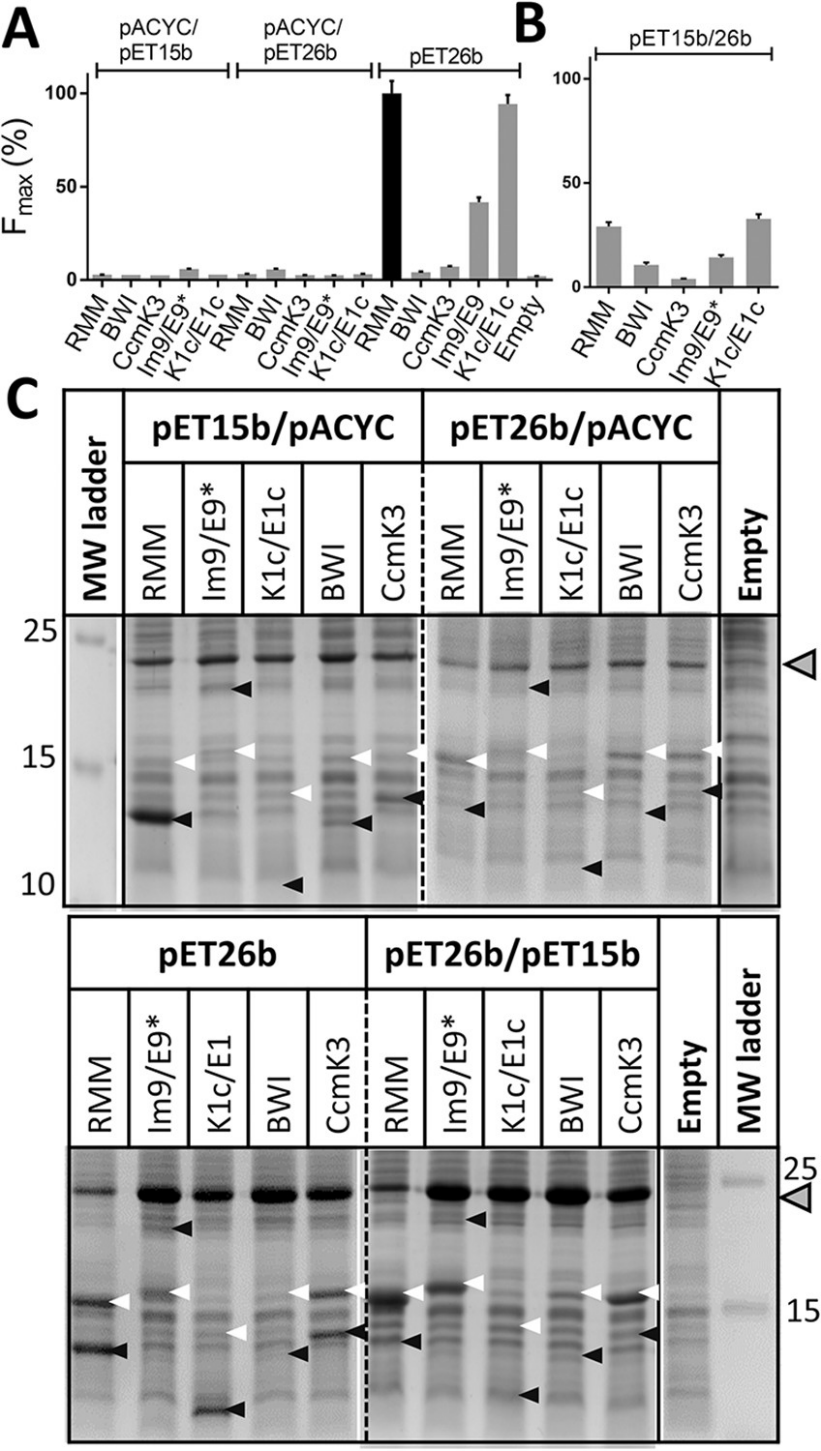
PPI detection when partners are expressed from separated vectors or not. **A, Absence of fluorescence signals when POIs are expressed from two compatible vectors.** Cells were transformed with either 1 vector coding for the 3 tGFP partners (pET26b) or with two different combinations of 2 vectors: a pACYC carrying GFP1-9 plus POI_1_-10 together with a pET15b for POI_2_-11 (pET15b/pACYC), or a combination of pET26b for POI_1_-GFP10 plus a pACYC for GFP1-9 and POI_2_-11 (pET26b/pACYC). A single POI name is given when the same POI is fused to GFP10 and GFP11 tags, otherwise the two components are separated by a slash. After fits to sigmoidal curves, F_max_ signals are given as percentages relative to the value measured for the RMM reference pair coded in a single vector (black bar). Data correspond to averages and standard deviations of at least two independent experiments, each one including several replicates per case (i.e. 3 clones), and are presented normalized because absolute fluorescence signals varied considerably among independent experiments. **B, Fluorescence induced from two incompatible vectors.** Signals were measured as in panel A but from cells transformed with a combination of pET26b carrying GFP1-9 and POI_1_-10 and pET15b with POI_2_-GFP11 (pET15b/pET26b). **C, Protein expression verification.** Total cellular extracts for different cases studied in panels A and B were analyzed by SDS-PAGE after 16 h induction. White arrows indicate the approximate migration of POI-10 species, whereas black arrows are for POI-11 partners. GFP1-9 position is given by grey arrows on the right. The molecular weights (kDa) of protein ladder components are also shown. Dashed lines are to indicate mounted independent gels.

The final aim of the study was to setup optimal parameters to screen cross-associations of BMC-H monomer pairs. In principle, molecular biology efforts to construct BMC-H pair libraries would be considerably reduced if the two POIs were expressed from independent vectors, when compared to using a single vector. For that reason, we privileged studies using BL21(DE3) strains transformed with two different combinations of compatible vectors: (1) a pACYC coding for the POI-GFP10 (POI-10) and GFP1-9 plus a pET15b carrying the POI-GFP11 (POI-11) or (2) a pACYC with the POI-11 alongside the GFP1-9 plus a pET26b coding for the POI-10. To our surprise, the fluorescence induced by the expression of the RMM-10/11 combination remained at levels comparable to the negative controls (BWI and CcmK3) and cell auto-fluorescence (Fig 1A). The same was also noticed for other combinations with expected positive PPIs, such as a combination of cationic and anionic K1/E1 coils [16], or the Im9/E9* pair (the *E*. *coli* immunity protein 9 and colicin endonuclease 9, the latter inactivated by mutation of the active site His575 into alanine to prevent toxicity that would result from a stoichiometric imbalance of E9/Im9 levels [17]). On the contrary, maximal fluorescence intensities (F_max_) were strong when expression of RMM, Im9/E9* or K1c/E1c pairs was induced from the same pET26b single vector (Fig 1A).

A detailed analysis by SDS-PAGE of cellular extracts collected at late culture stages indicated that the POI coded alongside the GFP1-9 was mostly absent when over-expression was carried out in cells co-transformed with 2-vectors (Fig 1C). The bands of the two POIs were more evident in the single vector strategy, especially with RMM and CcmK3 cases. An even clearer-cut view was obtained when cells transformed with each individual plasmid were analyzed similarly (S3 Fig). Only POIs coded by the pET15b or pET26b (*i*.*e*. in absence of GFP1-9) showed correct expression, whereas bands for the same POI were basically missing in combinations including the GFP1-9. Although part of difference might derive from lower copy numbers of pACYC with regard to pET vectors, the overall data pointed to a deleterious effect on POI viability caused by the known GFP1-9 insolubility [18]. This conclusion was corroborated using a set of diverse proteins described in the supplementary information file (see also S2 Fig).

Finally, similar experiments were performed using combinations of pET15b (coding for the POI-11) and pET26b (POI-10 and GFP1-9), a genetically instable combination, often termed incompatible. Interestingly, not only the RMM pair, but also K1c/E1c resulted in significant fluorescence, above signals of negative controls (Fig 1B), although values were still 3 to 4 times lower than with the single vector strategy. As for the other combinations of two vectors, a strong imbalance of POI-10/POI-11 ratios was observed on SDS-PAGE gels (Fig 1C).

### Impact of genetic organization

In light of previous results, single-vector configurations were adopted for subsequent experiments. Considering that BMC proteins are often coded within operons [3], and that the efficiency of oligomerization could vary depending on whether proteins are transcribed on the same mRNA or not [19–21], we sought to establish the optimal genetic organization scheme for studying BMC-H oligomerization. Three different constructs were considered: (1) leading to three independently transcribed partners (POI-10; POI-11; GFP1-9), (2) transcription of both POI-10 and POI-11 giving rise to a bicistronic mRNA segment, while GFP1-9 remained independent, and (3) all 3 partner sequences integrated in a single tricistronic mRNA.

**Fig 2:**
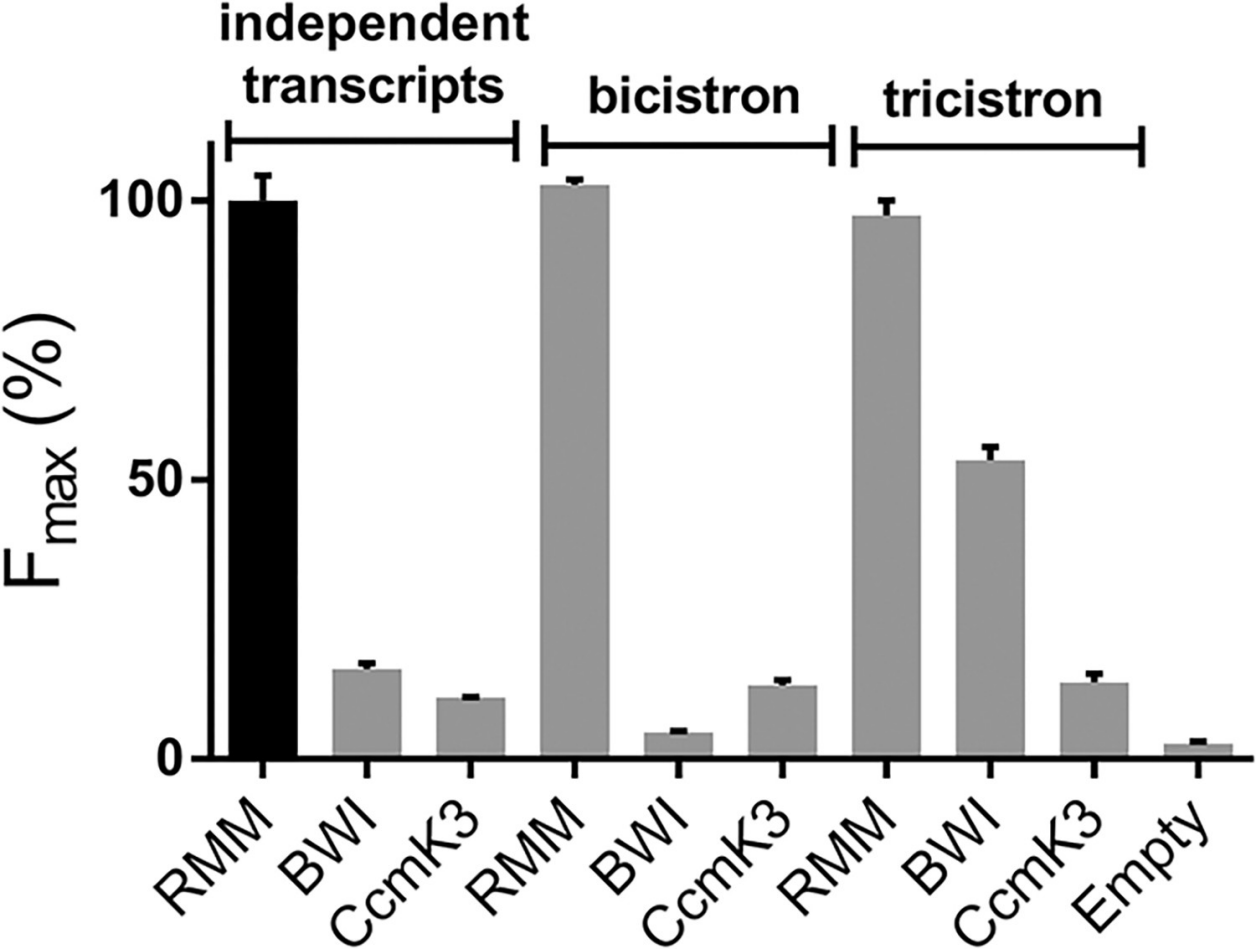
Effect of tGFP partner genetic organization on tGFP reconstitution. tGFP vectors were designed to give rise to three independent mRNAs encoding for POI-10, POI-11 and GFP1-9 (independent transcripts), to two distinct mRNAs, one coding for both POIs, the second for GFP1-9 (bicistron), or to a single mRNA encoding the 3 tGFP partners (tricistron). F_max_ are given as percentage of the value for the independently transcribed RMM pair (black bar). Other details are like in Fig 1.

A comparable strong fluorescence was recorded with all combinations implying RMM, irrespective of the genetic organization mode (Fig 2). Signals resulting from co-expression of CcmK3 monomers remained weak with the 3 organizations, slightly above cellular auto-fluorescence. However, while BWI fluorescence was low with vectors leading to independent or bicistronic transcripts, a neat increase of signal occurred when the three partners were transcribed together (tricistron), reaching approximately 50% of the signal induced by the RMM-coding tricistron. Consequently, the ratio of RMM versus BWI signals dropped from *ca*. 15 in the bicistronic construct to about 2 fold in the tricistron. The translation of the three partners in too close proximity seemed to boost the contribution of unspecific signals similar to those deriving from random encounters, suggesting that tGFP reconstitution might also be function of the spatial separation between biosynthetic *loci* of the different partners.

### Constitutive *versus* inductible expression

**Fig 3:**
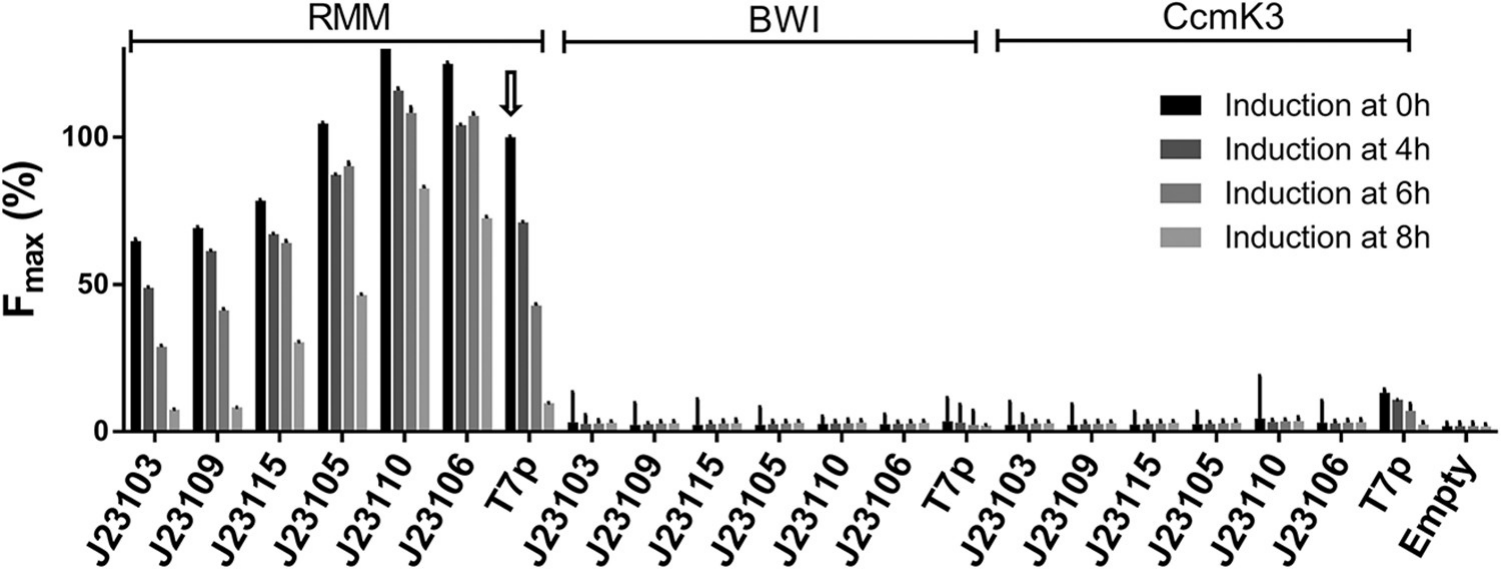
Promoter control over the transcription of the tGFP partners. Cells were transformed with plasmids permitting the transcription of the bicistronic segment coding for POI-10/11 under the control of either T7p or of indicated CPs. In all cases, the GFP1-9 coding segment was controlled with a T7 promoter (T7p). Cells were induced with IPTG at 0, 4, 6 or 8 hours of culture. As BWI and CcmK3 fluorescence values were too low, sigmoidal fit could not be performed to extract an F_max_ value. Their plotted values correspond to the highest fluorescence point over the course of the culture. F_max_ are given as the percentage of the T7p-controlled RMM pair value with induction from the beginning (indicated with an arrow).

Uncoupling the production of the two GFP10- and GFP11-tagged POIs from GFP1-9 expression might permit to reduce random encounter events and to limit interferences caused by the suspected precipitation of individual partners prematurely bound to GFP1-9. To achieve that, the two POIs of the abovementioned bicistronic constructs were placed under the control of a constitutive promoter (CP), while the GFP1-9 was maintained under the T7 promoter (T7p). In early experiments, sixteen different CPs were selected from the iGEM promoter catalog, and their impact on RMM signals was screened with IPTG added from the beginning of the culture (S4A Fig). Six CPs covering the fluorescence signal range were chosen to investigate the effect of inducing the GFP1-9 expression at different stages of bacterial growth (Fig 3). These CPs were also introduced in bicistron plasmids coding for BWI and CcmK3 negative controls, with the intention to detect possible improvements of signal ratios between positive PPI (RMM) and noise (BWI and CcmK3).

Promoters J23105, J23106 and J23110 CPs led to higher signals than the T7p reference when considering the RMM combination, whereas lower signals occurred with J23115, J23109, J23103 (Fig 3). Fitting of data to sigmoidal functions permitted to better quantify the impact of a postponed GFP1-9 induction. Thus, F_max_ decreased progressively when induction delays increased. As expected, the strongest effect was measured for the T7p reference. Surprisingly, strong declines also manifested with the weakest promoters. Indeed, F_max_ values after 0 h and 4h retarded induction were almost identical for J23103 and J23109, but dropped severely when postponing induction for longer times (see also S4B Fig), reaching for an 8 h delay barely 10% of values measured when IPTG was present from the beginning. Insufficient GFP1-9 production or decline of cellular resources were ruled out as arguments to explain such signal drop for weak CPs, as considerably higher fluorescence could be reconstituted with stronger CPs when inducing after 8 h of culture. Also noteworthy, postponing induction did not always cause the concomitant displacement of fluorescence emergence. This was clear for data collected after induction at 0 h or 4 h of culture, which resulted for instance in superimposed curves with J23103, J23109 and J23115 CPs. This certainly reflected the necessity to reach sufficient cellular densities for fluorescence detection, since 1.8 to 2.0 h displacements were calculated between the 4 h and 6 h-postinduction curves for these CPs. The difference declined to 1.5, 1.3 and 1.1 hours for the J23105, J23110 and J23106 CPs, respectively. These data, together with the fact that F_max_ diminished progressively when delaying induction, suggested that GFP1-9 was likely incapable of labeling a portion of the pre-formed RMM-10/11 cytosolic pool (discussed below).

### Changing linker length to monitor different associative phenomena

Linker length is known to impact the ability to detect PPIs. A large-scale study using a dihydrofolate reductase split assay proved that longest linkers allowed to capture a higher number of PPIs [22]. Yet, long linkers might also cause protein instability or interfere with protein association. Besides, the entropic benefit provided by the linker should theoretically decline above a certain length. On the other hand, too short linkers might perturb POI folding or render sterically incompatible PPI and/or reporter reconstitution. In this respect, β10 and β11-strands adopt an antiparallel orientation in the GFP structure. Sufficiently long linkers are thus required to cover the 36 Å distance that separates the alpha carbons of the two first residues of these strands (His199 and Asp216), when GFP10 and GFP11 are connected at the POI C-terminus.

**Fig 4:**
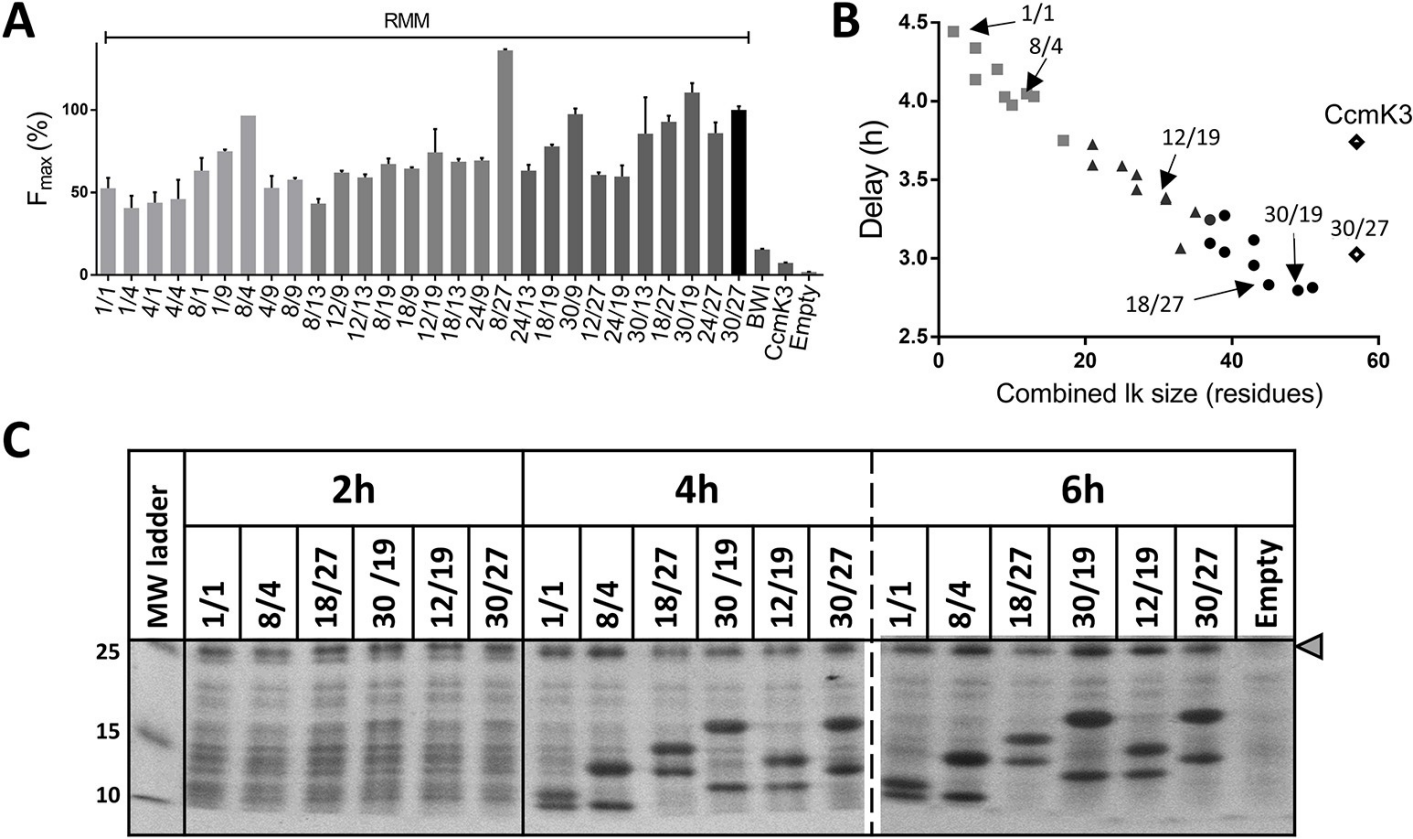
Influence of Linker length on tGFP reconstitution. RMM were connected to GFP10 or GFP11 peptides with flexible linkers (Lk, rich in glycine/serine) of varied sizes. For each construct, the residue number of the GFP10-connecting Lk is given first, separated by a slash from residue number of the GFP11- connecting one. **A, Maximal fluorescence signal according to Lk length**. The 100% reference case is RMM with Lk30/27 (black bar). Lk30/27 were also present in BWI, CcmK3 controls. Empty corresponds to BL21(DE3) transformed with pET26b. **B, Delay of fluorescence appearance.** The calculated time differences between the midpoints of fluorescence and cellular growth are presented according to the residues number sum of the two Lk in the construct. BWI value exceeded 6h in delay of fluorescence appearance and is not shown here. **C, Kinetics of tGFP partners expression depending on linker sizes.** Cells expressing GFP1-9 and RMM pairs connected by indicated Lk to the GFP tags (pointed by arrows in panel B) were collected 2, 4 or 6h after induction. After cellular lysis, soluble fractions were analyzed by SDS-PAGE. The dashed line indicates 2 independent gels. The grey triangle on the right indicates GFP1-9 migration position. Molecular weights (kDa) of protein ladder components are on the left.

The two linkers of the reference RMM construct were progressively shrunk from the original size (27 or 30 residues preceding GFP10 and GFP11 peptides, respectively) down to a single residue each (28 screened constructions). After transformation of BL21(DE3), cellular growth and fluorescence were monitored. The global trend was a decrease of F_max_ when reducing linker length, with approximately a 3 to 4-fold difference between extreme cases, depending on the experiment (Fig 4A). The combination of shortest linkers still fluoresced, although a single residue was initially considered structurally incompatible with correct tGFP reconstitution from monomers belonging to the same hexamer.

Interestingly, fluorescence not only decayed for the shortest linkers but was also delayed (Fig 4B). The time interval measured between midpoints of cell growth and of fluorescence increased from about 3 h with the longest linkers to *ca*. 4.5 h with the shortest. The delay was even longer (6 h) for the BWI control (with Lk30/27), something that we attributed to random collision events (or low-affinity PPI). To rule out differences of protein accumulation kinetics as cause of different delays, protein expression levels were monitored at different culture times (Fig 4C). Six combinations exhibiting extreme and intermediate lag times were analyzed: Lk1/1, Lk8/4, Lk12/19, Lk18/27, Lk30/19 and Lk30/27. Band intensities on SDS-PAGE gels demonstrated comparable protein accumulation rates.

Potential alterations of tGFP reconstitution capabilities induced by linker length were investigated with *in vitro* experiments. Supernatants containing the soluble protein fraction from cells over-expressing RMM-10/11 couples, either carrying Lk1/1 or Lk30/27 connectors, were reacted with His-tagged purified GFP1-9 following a described protocol [9]. SDS-PAGE band intensities proved to be almost identical for the two protein pairs. However, fluorescence signals with the shortest linkers barely reached 10–15% of values attained in reactions with Lk30/27 supernatant, therefore proving their considerably different intrinsic reactivity.

Collectively, these data suggested that tGFP fluorescence was reporting on different phenomena: signals deriving from intra-hexameric oligomerization would dominate with long linkers, while inter-hexamer labeling implying freely diffusing hexamers or hexamers juxtaposed in assemblies, would be the main source of fluorescence with short linkers.

### Contribution of BMC-H macrostructures

BMC-H often coalesce to form highly-ordered macromolecular objects when overexpressed inside cells, or *in vitro* from purified proteins [14, 15]. As exposed above, contacts between assembly subunit neighbors could contribute to fluorescence readings. We therefore applied several strategies to quantify such contributions to tGFP signals. First, as an attempt to prevent assembly formation, mutations were introduced in RMM peripheral residues shown to participate in hexamer-hexamer contacts [13, 14, 23–25]. The K26D RMM single mutant (sm-RMM) was selected based on published transmission electron microscopy (TEM) data that demonstrated an absence of assembly for the corresponding PduA mutant [24]. We also included the N29D/A53D double mutant (dm-RMM) and a combination of all three mutated positions: K26D/N29D/A53D (tm-RMM).

Before recording fluorescence signals, we sought to verify whether introduced mutations perturbed assembly behavior. Oligohistidine C-ter tagged proteins were first analyzed by TEM, after overexpression *in E*. *coli*. Well-defined bundles of nanotubes were visible for the wild-type (WT) RMM (S5A Fig). Although agreeing with the literature, nanotube diameters measured in transversal (21 ± 2 nm) or longitudinal views (19 ± 3 nm) were significantly bigger than reported 14.1 nm values [26]. Surprisingly, similar structures still formed with dm-RMM (21 ± 2 nm, transversal view) (S5B Fig). Nanotubes were not evidenced for any of the two mutants incorporating the K26D mutation (sm-RMM or tm-RMM). Yet, compact assemblies continued to form with the sm-RMM, which often looked like rolled 2D layers (11 ± 2 nm inter-spacing). Failure to fully disrupt assemblies prompted us to directly image the strains prepared for tGFP screens (S6 Fig). Nanotubes were still visible with the bicistronic reference WT RMM-10/RMM-11 case with Lk30/27 (also expressing GFP1-9). Yet, bundles were less evident than with His-tagged versions, being often interrupted in longitudinal cell views. Agglomerates were noticed close to cellular poles for tm-RMM. Less evident to interpret, sm-RMM-10/11 and dm-RMM-10/11 overexpression resulted in massive protein accumulation inside cells (S6B, S6C Fig). Although motifs reminiscent of spatial organization were not really identified, data did not permit to fully rule out assembly occurrence. Of note, mutations did not cause a significant deviation of protein levels, according to SDS-PAGE band intensities (S6E Fig), exception made of the tm-RMM-10/11 case that resulted in fainter band intensities.

**Fig 5:**
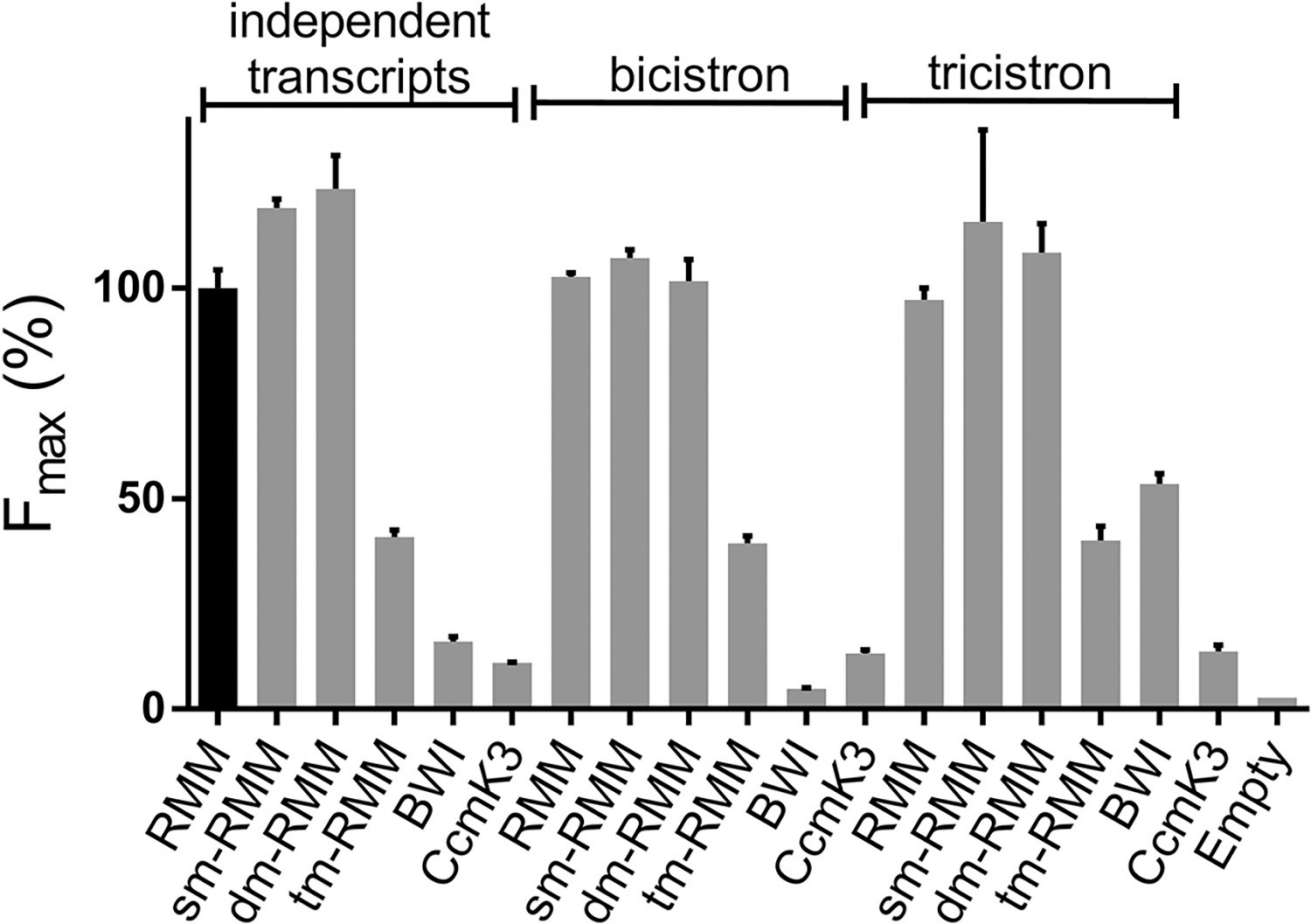
Constant tGFP reconstitution in the presence of mutations impeding BMC-H assembly. F_max_values were estimated from fits to sigmoidal curves of data collected over 16 h, with induction from the culture start. In the case of BWI, CcmK3 and other cases with too low fluorescence values, fits were not reliable, and the value plotted corresponded to the highest fluorescence point over the course of the culture. Plotted values are given as the percentage with regard to the reference case: RMM-10/11 with Lk30/27 in an independent-transcript organization mode (black bar).

When fluorescence signals were monitored, both sm-RMM and dm-RMM resulted in F_max_ slightly higher than the WT case (Fig 5). A significant drop was only noticed for tm-RMM. The trend remained the same independently of the precise genetic organization mode chosen (using single vectors). Moreover, similar delays were measured between midpoint of cellular growth and fluorescence curves: WT-RMM (3.4 h), sm-RMM (3.5 h) and dm-RMM (3.4 h). The value increased considerably for tm-RMM (5.6 h). Collectively, comparable data collected for WT-RMM and possibly non-assembling sm-RMM or dm-RMM would support that assembly-deriving events contributed weakly to recorded signals (for cases connected *via* long linkers).

**Fig 6:**
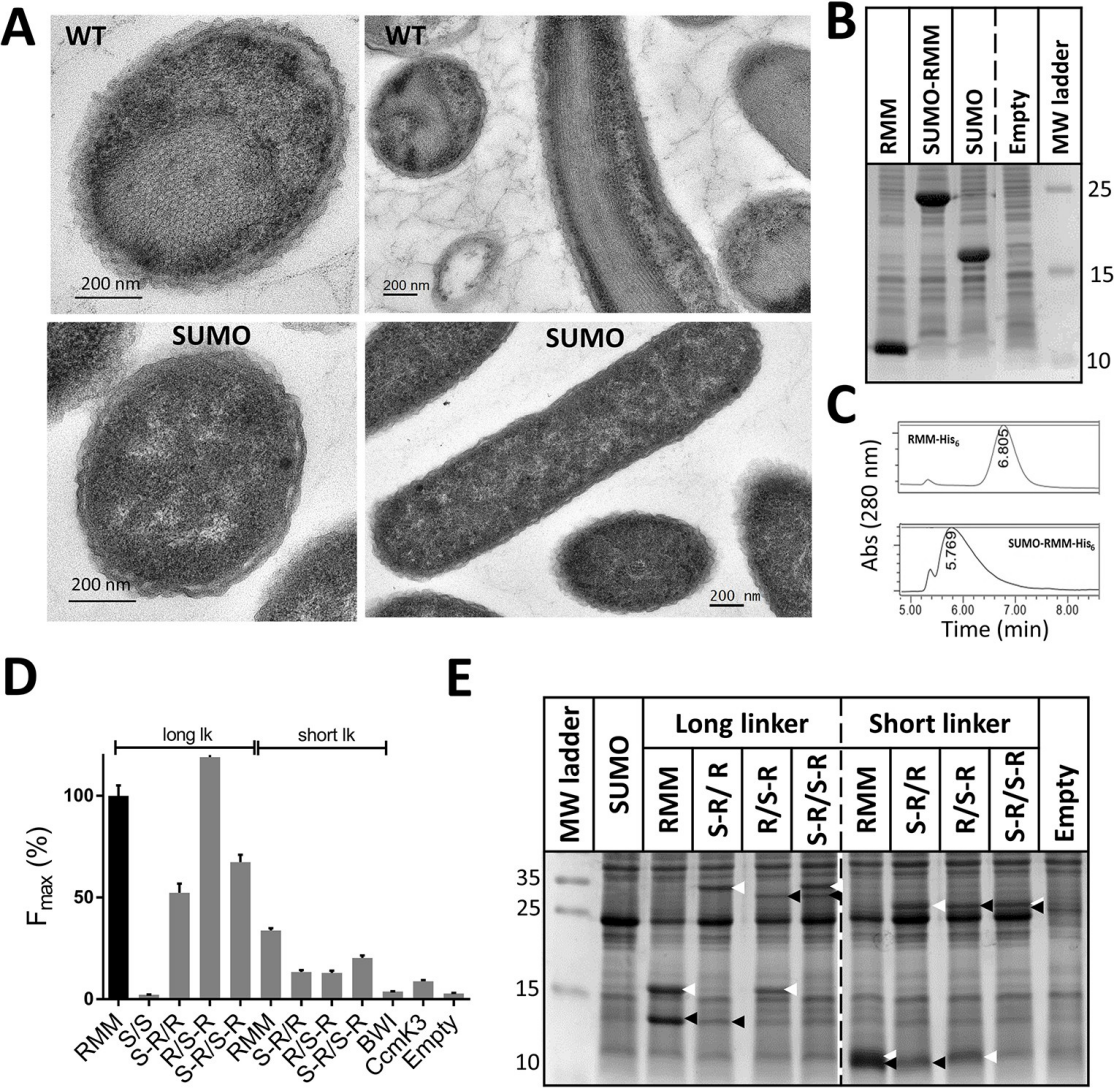
Fluorescence reconstitution with assembly-impaired RMM constructs. **A, Effect of SUMO domain on RMM ability to assemble.** Cells over-expressing RMM monomers in N-ter fusion to the SUMO domain or in its absence were imaged by TEM. **B, Protein expression levels.** Total cellular contents were analysed by SDS-PAGE. SUMO corresponds to expression of a C-ter His-tagged SUMO domain, and Empty is for cells transformed with pET26b **C, SUMO-RMM solubility.** TALON-purified samples were analysed by size-exclusion HPLC. Retention time for RMM was slightly above values expected for an hexamer (82 kDa estimated, 64 kDa theoretical). A 290 kDa size was estimated from the retention time of the sumoylated version, possibly suggesting a dimer of hexamers (theoretical 130 kDa). Aggregated species (> 2 MDa) elute within the 5.4 min peak. **D, Effect of SUMO presence on the tGFP signals.** Cells expressing the RMM pair or different combinations of SUMO-RMM with RMM (connected to GFP10/11 with either long lk30/27 or short lk1/1) were assayed for fluorescence reconstitution. F_max_ values are given as the percentage of the value of RMM reference with a lk30/27 (black bar). BWI and CcmK3 controls had the long linkers. **E, Protein expression.** Only the total cellular fractions were analysed on SDS-PAGE. R and S labels stand for RMM and SUMO, respectively. Other details are like in Fig 1.

Disruption of assemblies was also attempted with sterically-hindering SUMO domains. This approach was successfully exploited to reconstitute synthetic minimalist BMCs *in vitro*, starting from highly concentrated hexamers kept in solution in connection to the SUMO domain [27]. Insertion of the SUMO domain at the N-terminus of a His_6_-tagged RMM was confirmed to fully prevent nanotube formation in *E*. *coli* (Fig 6A), in spite of comparable expression levels to the non-modified version (Fig 6B). Subsequently, SUMO was fused similarly to RMM-10, RMM-11, or to both partners of the reference RMM case (with Lk30/27). Assembly absence was again confirmed for all 3 tGFP-screened cases by TEM (S7 Fig). When fluorescence was monitored, F_max_ for the RMM/SUMO-RMM (R/S-R) case corresponded to 115% of the value for RMM pair reference, whereas signals decreased to about 50–65% for the other two constructs (Fig 6D). Intensity of SDS-PAGE bands indicated, however, that partners in combinations with SUMOylated constructs were expressed with considerably lower yields than the unmodified RMM pair (Fig 6E). Accordingly, relatively similar F_max_ values would again suggest that a portion of the RMM-10/11 pool must remain unlabeled, not contributing to fluorescence reconstitution.

Contrasting with data for cases with long linkers, a more severe decline of fluorescence was induced by SUMO when RMM were connected to GFP10 and GFP11 with Lk1/1 (Fig 6D). For the three different constructs, F_max_ were barely above background levels, again supporting that the more delayed fluorescence signals with short linkers (Fig 4B) might derive from associative phenomena other than oligomerization.

### Oligomerization of BMC shell components

The tGFP technology was next evaluated as means to study the oligomerization of the three different families of proteins that compose BMC shells, namely BMC-H, BMC-T and BMC-P. The next well-described BMC-H were assayed with a single vector set-up, including POI-10/11 bicistronic organization and Lk30/27 connectors: PduA from *S*. *enterica*, CsoS1A from *H*. *neapolitanus*, *H*. *ochraceum* (HO) BMC-H, EutM from *E*. *coli* and CcmK4 from *Syn6803*. Preliminary results pointed out a deleterious effect of C-terminal tagging with CsoS1A and CcmK4 [6]. Hence, GFP tags were placed in N-terminus with these two cases. This was also done for RMM, with the intention to evaluate the impact of tag orientation on signals.

**Fig 7:**
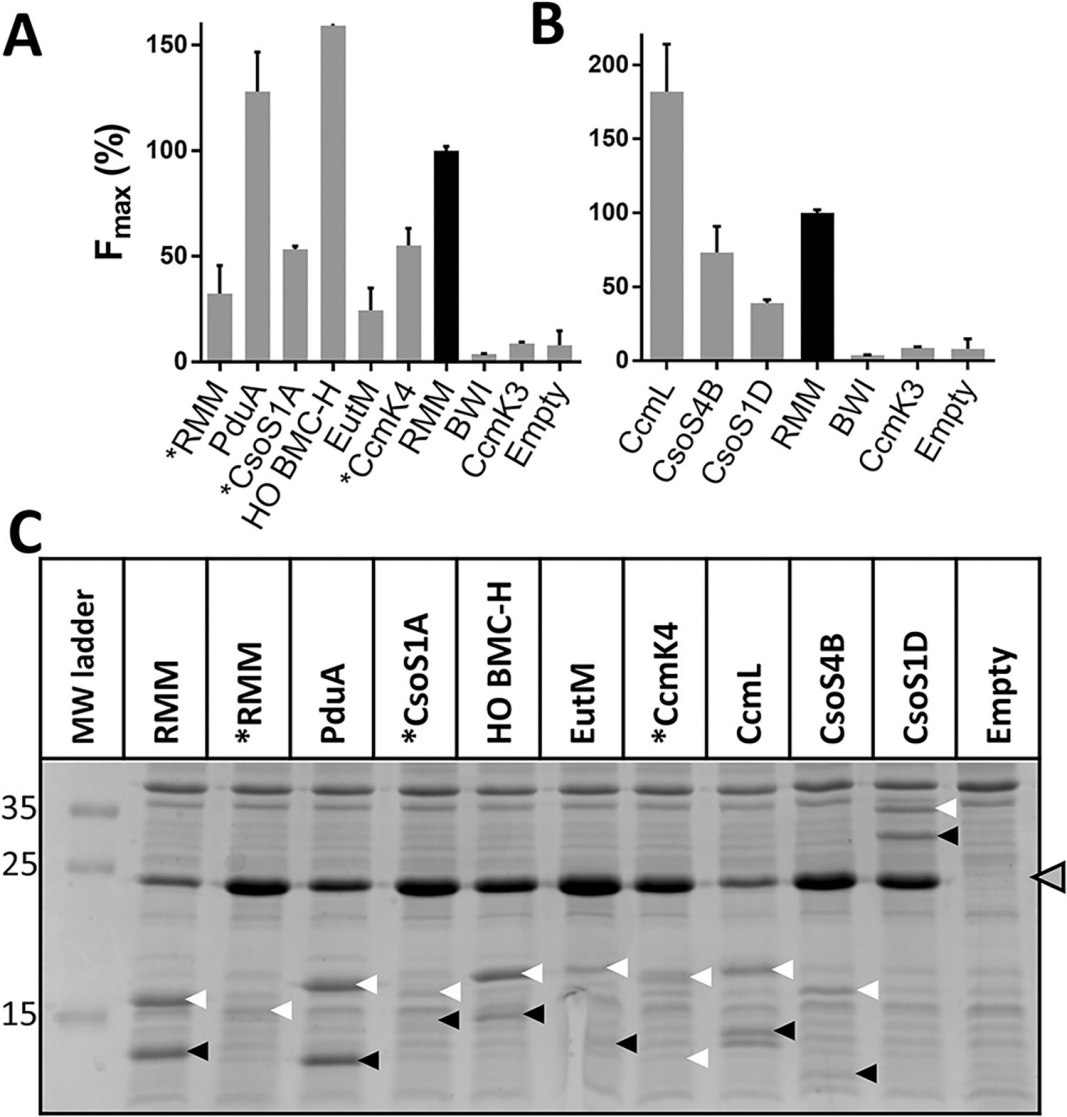
tGFP assay to monitor the oligomerization of BMC shell components. **A, tGFP reconstitution informs on homo-hexamer formation.** Fluorescence values with pairs of indicated proteins organized in a bicistronic mode with Lk30/27 connectors. **B, detection of association of BMC-P and BMC-T components.** tGFP assay was performed similarly but on CcmL and CsoS4B BMC-P and on the CsoS1D BMC-T. F_max_ presented in A and B panels are given as the percentage with regard to values measured for the reference RMM control (black bars). **C, Verification of protein expression.** Total protein contents extracted at late growth phases from panels A and B were analyzed by SDS-PAGE. White and black arrows point at presumable POI-10 and POI-11 bands, respectively. The asterisk notifies combinations of GFP10/11 N-ter-tagged monomers. See Fig 1 for other details.

tGFP reconstitution occurred with all BMC-H cases, in agreement with expected hexamer formation (Fig 7A). Signals were especially strong with PduA and HO BMC-H. On the contrary, fluorescence induced by EutM-10/11 overexpression remained low, albeit above threshold levels established by the BWI and CcmK3 negative controls or cellular auto-fluorescence measured in cells carrying an empty vector. Variations of the relative intensities of SDS-PAGE bands partially explained such signal disparities (Fig 7C). However, expression differences were too strong (e.g. > 10-fold when comparing CsoS1A or CcmK4 to PduA or RMM) as to correctly fit to fluorescence changes (2 to 2.5-fold between the same cases). Again, this suggested that a portion of the hexamer pool escaped labelling by the GFP1-9 when BMC-H is expressed strongly. A similar conclusion could be reached when considering N- and C-ter tagged RMM combinations. Switching tag orientation caused a 3-fold fluorescence drop, though bands from N-ter tagged RMM-10/11 species were almost imperceptible.

The tGFP was also found to be suitable for the study of BMC-T and BMC-P shell components. This was demonstrated with the CsoS1D BMC-T from *H*. *neapolitanus* and two BMC-P, the CsoS4B from the same organism and CcmL from *Syn6803*. Fluorescence readings were highest with the pentameric CcmL (Fig 7B), a stoichiometrically-minor component of cyanobacterial β-carboxysome shells [28, 29]. Substantial fluorescence also emerged with CsoS4B from α-carboxysomes. Signals with CsoS1D were lower but still superior to threshold values established by negative controls. SDS-PAGE analysis indicated that CsoS1D fluorescence drop could not be attributed to an eventual protein expression failure, since the two partners were correctly detected (Fig 7C).

### Hetero-oligomerization assessed with the tGFP technology

A genomic survey estimated the existence of an average per organism of about 3.5, 1.4 and 1.2 gene copies coding for monomers associating as BMC-H, BMC-T and BMC-P, respectively [3], genes being most often organized within the same operon. Two studies evidenced that the simultaneous co-expression of BMC-H from gene paralogs might conduct to hetero-hexamer formation [6, 7], but such possibility has not yet been fully investigated. The tGFP technology might be a valuable tool to explore such processes.

**Fig 8:**
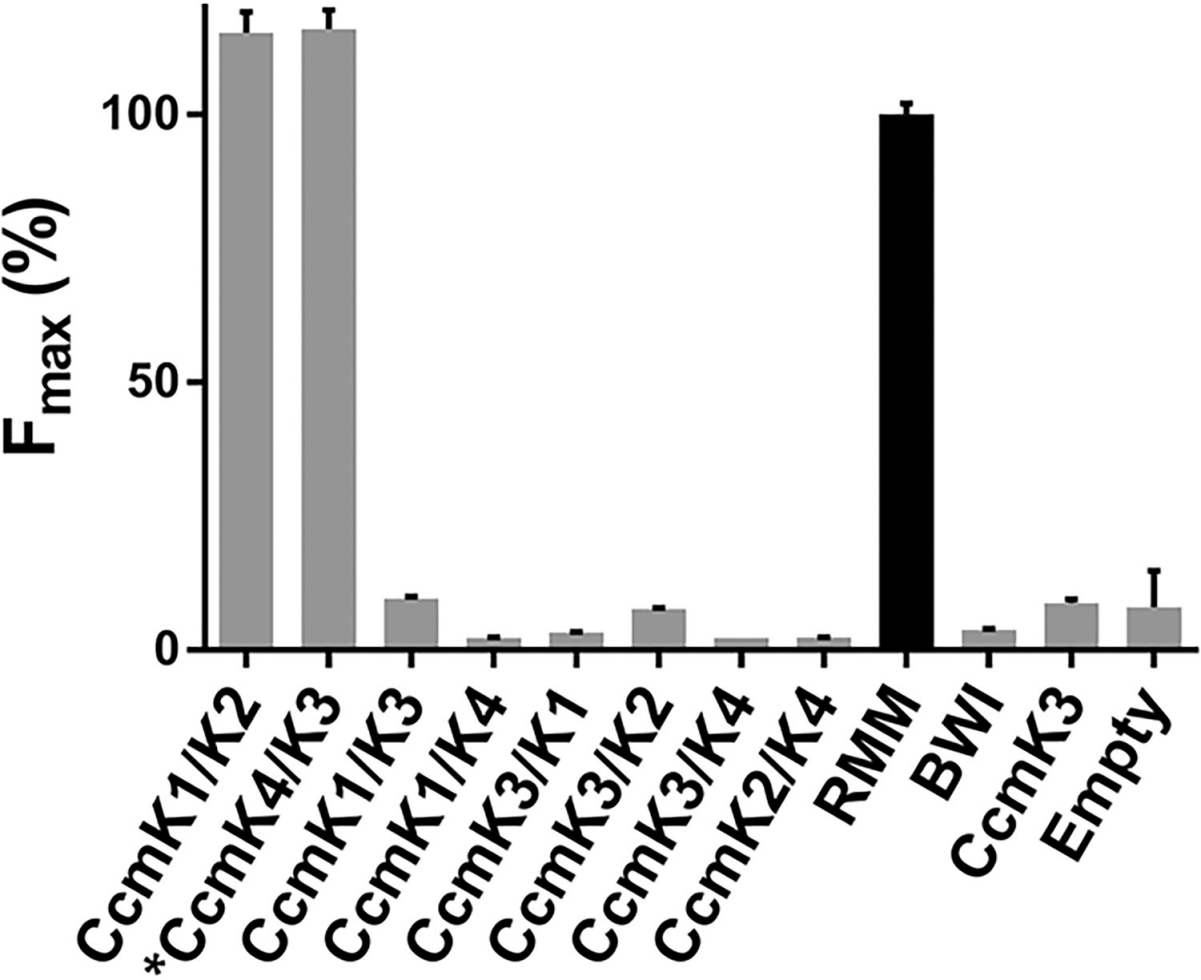
Hetero-hexamer formation followed with the tGFP assay. Fluorescence reconstitution occurring in cells expressing the indicated combinations of CcmK proteins from *Synechocystis sp*. *PCC6803* under a bicistronic scheme with Lk30/27 was monitored. Plotted values are give with regard to RMM reference case (black bar). The asterisk is to signify the presence of a GFP10 or GFP11 peptide connected on the monomer N-terminus. See Fig 1 for other details.

In this last section, we therefore sought to validate the screen as tool to study hetero-hexamer formation. Several GFP10/11-tagged pairs combining CcmK1, CcmK2, CcmK3 and CcmK4 genes from *Syn6803* were constructed in plasmids with a bicistronic organization and screened after transformation in BL21(DE3) cells, as before. Remarkably, results presented in Fig 8 reproduced published data [6], which were obtained following a Western blot (WB) approach. Namely, only the CcmK4/CcmK3 and CcmK1/CcmK2 combinations resulted in positive readouts, with estimated F_max_ that were even higher than those from the RMM homo-hexamer reference case. Moreover, as reported, CcmK4/CcmK3 signal depended on tagging orientation, the F_max_ dropping to background levels when the GFP10 peptide was transferred from N-terminus of CcmK4 towards its C-terminal side.

## Discussion

The increasing availability of genomic data permitted to localize BMC coding genes in approximately 20% of bacteria and at least 45 different phyla [1]. New BMC types are constantly discovered, summing up nowadays close to 70 different BMC types and subtypes. Moreover, around 20% of BMC-containing bacteria have 2 BMCs and 2% of them encode even more. The uttermost organism is the extremophile *Malediviba halophilus* that carries 6 BMC loci [1]. Human pathogens, such as *Salmonella enterica* or *Klebsiella pneumoniae*, also count with the possibility of expressing several compartments. BMC presence offers a competitive advantage to these organisms and is often correlated with strain virulence [30]. Despite this co-occurrence of BMC types in given organisms and the existence of multiple paralogs coding for shell components, often arranged in operons, no study has ever reported on hetero-oligomer formation other than the CcmK hetero-hexamers of β-carboxysomes from two cyanobacteria [6, 7]. This is probably justified by cumbersome work required to label each protein individually, before monitoring cross-associations by classic methods like fluorescence microscopy colocalization. In this study, we demonstrated that the tGFP technology, which is amenable to mid/high throughput screening, is an appropriate tool to fill this gap of knowledge. First, tGFP correctly informed on the oligomerization of 6 different BMC-H (PduA, RMM, CsoS1A, HO BMC-H, EutM and CcmK4), 1 BMC-T (CsoS1D) and 2 BMC-P (CcmL and CsoS4B). Most notably, the approach also faithfully corroborated the results reported on the hetero-hexamerization of pairs of CcmK1 to CcmK4 proteins from *Syn6803*, which had been generated using a WB detection experimental approach [6]. Only the 2 cases implying CcmK1/CcmK2 and CcmK3/CcmK4 combinations that were expected to cross-interact, according to previous results, were positive, among a total of 8 combinations screened. The sensitivity to tag orientation was also reproduced, a positivity cross-interaction being revealed with CcmK3 only when CcmK4 was labeled in N-ter.

Our study also revealed a pull-down effect exerted by GFP1-9 on co-expressed partners. Indeed, GFP1-9 solubility was already known to be poor [18]. The effect was first unveiled when combining independent plasmids, and confirmed for protein pairs that were mounted to evaluate the robustness of the tGFP assay (described in the Supplementary information, see also S2 Fig). Such GFP1-9 property is probably benefic for PPI studies, since the fluorescence caused by protein accumulation is reduced, hence decreasing false positives. However, deleterious consequences are the constraints imposed on the experimental design, for instance, or the difficulties to confirm correct partner expression, something required to better guarantee an absence of PPI. GFP1-9 pull-down effect on co-expressed POI-10 or POI-11 species was especially strong when the two POI partners did not associate with each other. We also noticed a recurrent band absence or signal decline on acrylamide gels (and concomitant F_max_ drop) for POIs encoded close to GFP1-9 when genetic information permitting production of the three partners was split in two separated compatible vectors (different ORI). On the contrary, both protein detection and F_max_ increased not only when a single-vector strategy was adopted, but also when the three coding elements were provided from two incompatible plasmids (same ORI). In that respect, it is known that plasmids with different replication origins may cluster separately in the bacterial cytoplasm [31, 32]. Translation could then happen separately, if generated transcripts stayed in proximity of their respective vectors. Signal improvements presented here with incompatible plasmids supported such scenario. However, it is presently unclear whether translation is spatially-coupled to transcription or instead happens from freely-diffusing mRNA [33].

Whatever the scenario, our data hinted at tGFP reconstitution being diffusion-limited. In early phases of the study, we assumed that the separated expression of each BMC-H partner might lead to homo-labeled hexamers (*e*.*g*. RMM carrying six GFP-10), reflecting the need to rapidly complete a full hexamer to prevent exposition to the aqueous milieu of extended hydrophobic surfaces involved in monomer-monomer contacts. However, the hypothesis was ruled out by similar results obtained with pairs of interacting partners that are soluble as monomers, such as combinations of Im9 and E9*, or of K1 and E1 coils. This prompted us to envision a different explanation: in the absence of one of the two POI-10/11 partners in the immediacies of the GFP1-9 biosynthetic site, due to the short lifetime and restricted volume accessed by GFP1-9 (or partially reconstituted species), the latter would cause precipitation of the most closely-expressed partner. We hypothesize that only highly soluble/stable proteins like TrxA might still be viable when attached to the GFP1-9 in the absence of the third partner.

Attempts to improve signals with CPs failed. We intended to let the necessary time to constitute a cytosolic pool of POI-10/11 species before triggering GFP1-9 expression, something that might reduce signal loss associated to the above-mentioned GFP1-9 insolubility. Instead, F_max_ declined in an unexpected manner (Fig 3 and S4B Fig): steadily but softly for strong CPs, in a sudden manner but only after 6h or longer delays with weakest CPs. This also occurred for the reference case with T7p, which seemed justified since it implied delayed expression of both GFP1-9 and POI-10/11. An insufficient GFP1-9 titer with regard to POI-10/11 species might be argued to explain F_max_ drops when retarding the induction with strong CPs. Yet, this possibility was neither supported by SDS-PAGE data presented throughout the study, which demonstrate GFP1-9 band intensities being often stronger than those of POI-10/11 partners, nor by the considerably higher fluorescence measured with other POIs (e.g. PduA, HO-BMC-H and CcmL pairs, Fig 7). Interestingly, only about 5–10% of the total reactive POI-10/11 pool contributed fluorescence when induction was postponed by 8 h with the weakest CPs (total pool being defined by signal levels attained when induction was triggered from the start). Levels reached with strong CPs ruled out an insufficient GFP1-9 expression or exhausted cellular resources. Rather, we interpreted such observations as indicative of the emergence of assemblies that would act as a molecular sink, absorbing freely-diffusing RMM hexamers without contributing significantly to fluorescence. Once absorbed, labeling of hexamers with GFP1-9 would be impeded or less efficient, either because GFP10/11 peptides would not be sufficiently exposed, or because such structures would nucleate in cellular regions distant from the GFP1-9 biosynthetic loci. Indeed, we could visualize nanotubes inside cells after only 4 h of induction of His_6_-tagged RMM expression (under T7p control, S8 Fig). A higher affinity for hexamer absorption, when compared to the association of GFP10/11 peptides with GFP1-9, might conduct to higher relative losses of signal with the weakest CPs. A slower biosynthetic yield with weak CPs would also retard assembly nucleation (probably occurring in the 4–6 h interval), explaining the superimposed fluorescence curves for 0 and 4 h induction times with these CPs. Nanotubes would happen inside cells before 4h with strongest CPs, also with the T7p reference, explaining the constant F_max_ decline.

Additional observations supported that part of the RMM-10/11 cytosolic pool, *i*.*e*. hexamers trapped within assemblies, remained inaccessible to reaction with GFP1-9. F_max_ values of assembly-forming proteins like RMM, PduA or HO BMC-H did not seem to correlate with their intense expression levels, as comparable signals were attained for cases displaying much lower expression levels, like CsoS1A or the combination with N-ter tagged RMM (Fig 7). Even stronger fluorescence occurred with non-assembling cases like K1c/E1 coils and CcmL, also with SUMO-modified RMM-10/11 (with long linkers), which again demonstrated significantly fainter SDS-PAGE bands than those of RMM pairs. Unproductive labeling might be justified in part by the small declines of GFP1-9 levels expected for strongly co-expressed POI-10/11. However, collectively, our results indicated that the formation of diffusion-constrained assemblies, coupled to the impossibility of GFP1-9 to travel far enough in the cytosol, would be the main cause. Steric hindrance of GFP10/11 peptides due to BMC-H embedding within assemblies or an impossibility to reconstitute tGFP if tags were oriented towards the nanotube lumen appeared less likely, in view of data reported for PduA [34]. This protein also readily forms nanotube bundles, which were successfully purified, less clearly labeled, using nickel-NTA chemistries that implied interactions with the PduA N-terminal His_6_ tags. Of note, an identical assembly bending orientation is predicted for the two BMC-H, according to molecular dynamics simulations [14].

The fluorescence signal with CsoS1D was also lower than expected from SDS-PAGE band intensities (Fig 7). This might point to the formation of assemblies, something described for other BMC-T [34]. Alternatively, homo-GFP10 or -GFP11 tagged trimers might be enriched by mechanisms involving the association of subunits emerging from adjacent ribosomes acting in *cis* on the same mRNA [21], if such events were facilitated by the bi-domain nature of BMC-T. Sterical prevention of tGFP reconstitution in sandwiched trimers, like those characterized in many BMC-T crystal structures [35], and in cryo-EM structures of several BMCs [5, 27, 36–38], seems unlikely. As indicate the 3D structure of *P*. *marinus* CsoS1D [39], tags attached to N- and C-terminal residues should be sufficiently exposed as a consequence of a secondary structure permutation. Nor stoichiometric considerations would suffice to justify the observed differences. Thus, an analysis of the different ways of concatenating two monomers A and B in a nascent oligomer indicates that 50% of A and B monomers would be fluorescent-unproductive in a trimer, compared to 30% in a hexamer. These proportions would correspond to homo-labeled oligomers and to stoichiometric imbalances between GFP10 and GFP11 tags in hetero-labeled species.

A significant retard of emergence of fluorescence, with regard to culture growth, was noticed for RMM-10/11 with short linkers, when compared to long linkers. This contrasted with expression profiles that presented almost identical kinetics. *In vitro* reconstitution confirmed the difficulties to reconstitute tGFP fluorescence when only 1 residue separated GFP10/11 tags from the RMM hexamer core. However, it remains unclear whether fluorescence with short linkers derived from random encounters of hexamers, which would be promoted by protein accumulation inside cells in later growth phases, or it corresponded to residual slow labeling of assemblies. Nor should be ruled out effects of linker size on fluorophore maturation kinetics [40], which were not investigated here.

The tGFP technology might also be appealing to monitor more complex interactions established during formation of BMC-H or BMC-T assemblies *in vivo*, or even for the study of BMC biosynthesis. Up to now, the experimental characterization of BMC assembly is being addressed with fusions of individual components to GFP variants [41–43]. GFP barrels are relatively bulky, with molecular weights that approximately double those of BMC-H or -P monomers. In light of that, and the demonstrated compactness of many BMC, caution should be taken when interpreting such data, since potential alterations of the mechanistic pathway induced by the experimental design cannot be ruled out. tGFP might be a more appropriate technique, as the small size of GFP10 and GFP11 peptides should perturb assembly to a lower extent. We believe, however, that the problematic related to the insolubility of GFP1-9 needs to be corrected before exploiting the technology for these purposes, otherwise assemblies might be unreachable for labeling. Similarly, the technology might be chosen to investigate hetero-oligomer occurrence in native BMC. Yet, an inspection of structural models of PduA and RMM assemblies highlighted the difficulties to select optimal GFP10/11 tag-anchoring residues that permitted to discern reconstitution deriving from intra-hexamer monomers or monomers from neighbor hexamers of the assembly.

## Materials and methods

All restriction enzymes and other molecular biology tools were from Thermo Fisher Scientific, unless otherwise indicated. Culturing media were prepared in doubly-osmosed tap water.

### Plasmid preparation

A receptor-1 vector (List S1 in S1 File) carrying coding segments necessary for ulterior expression from 3 independent transcripts of GFP10 (first cassette) and GFP11 (second cassette) preceded of Lk30/27 linkers, together with GFP1-9 (C-ter His_6_-tagged, third cassette), was prepared from a fragment provided by Genecust that was integrated between BglII and BlpI sites of pET26b (Supplementary information). After digestion with XbaI/XhoI, the first two cassettes were independently cloned in pET15b or pET26b previously opened with the same enzymes. POI-10 and POI-11 sequences were prepared from them after exchanging POI (Twist Biosciences, List S1 in S1 File) with NdeI/NotI digestions. These constructs served for studies with combinations of single plasmids. Besides, they permitted transfer to the first or second cassette of Receptor-1 of corresponding DNA stretches recovered with SwaI/BamHI or MunI/SalI digestions, respectively. Ligations were carried out with the T4 DNA ligase. POI combinations involving BMC-P and BMC-T, as well as BMC-H other than RMM-10/11 or CcmK3-10/11, together with combinations for studies of hetero-hexamerization, were prepared following Gibson assembly protocols (see SUMOylated cases presented below, List S2 in S1 File). After transformation of chemically competent TOP10, plasmids were recovered from selected clones using Miniprep protocols.

Bicistronic vectors were prepared from 3-transcribed vectors by digestions with BamHI/MunI to remove T7 terminator (T7t) and T7p sequences between the first and second cassettes. Overhangs were completed by Klenow fragment before plasmid circularization. A similar procedure was applied to prepare tricistronic cases from bicistronic ones, using SalI/PacI to eliminate T7t and T7p between the second and third cassettes.

Studies with combinations of vectors were performed with pET26b and pET15b constructs prepared above. For preparation of pACYC vectors, we first removed an XbaI site present in the p15A replication origin of pACYC_Duet1 (Novagen) using primers PrXb_For and PrXb_Rev (List S3 in S1 File). This vector was opened with NcoI/BlpI enzymes and ligated with similarly digested DNA fragments (Twist) comprising GFP1-9(His_6_) and either RMM-10 or RMM-11 coding sequences. POIs-10/11 sequences were exchanged as for preparation of pET26b or pET15b, by means of XbaI/XhoI restriction sites. pET26b vectors permitting coexpression of GFP1-9(His_6_) and POI-10 were prepared from bicistronic vectors with MunI/SalI treatments and circularization of blunt ends generated with Klenow fragment.

Linker length was modified by PCR with Phusion polymerase (provider protocol) using different combinations of primers (IDT), as indicated in List S3 in S1 File, and taking as template vectors carrying Lk30/27. PCR products were purified on gel, circularized and amplified in TOP10 bacteria.

A total of 16 constitutive promoters were selected from the iGEM part repertoire (sequences indicated in List S4 in S1 File). To replace the first T7p in the bicistronic case combining RMM-10/11 by CPs, the vector was digested with BglII/SwaI and ligated with 5- phosphorylated oligonucleotide pairs (IDT), previously annealed following a protocol that brought the mixture from 95 to 30°C in 5°C steps lasting 30 seconds each. In a second phase, the same protocol was applied to BWI-10/11 and CcmK3-10/11 bicistronic vectors using only the six selected promoters BBa_J23103, BBa_J23105, BBa_J23106, BBa_J23109, BBa_J23110 and BBa_J23115.

SUMO-RMM-His_6_ was ordered from Twist Bioscience cloned in a kanamycin (kan)-resistance carrying pET29b. Fragments coding for SUMO-RMM-GFP10 and SUMO-RMM-GFP11 with either long or short linkers were ordered from TWIST (List S2 in S1 File). These fragments incorporated 15–22 nucleotide-long flanking segments to permit the one-step integration by Gibson assembly (NEBuilder HiFi DNA Assembly Master Mix) of the two POIs in an NdeI/SalI-opened receptor2 vector. The latter was identical to the bicistronic and T7-transcribed RMM-10/11 case deriving from receptor1, which had been modified to remove NdeI and NotI sites that directly flanked the GFP1-9 sequence.

For every case, final vectors were verified by both enzymatic restriction and sequencing (Eurofins) with primers Pr_For and Pr_Rev (List S3 in S1 File), depending on the cassette of interest.

### Monitoring culture growth and fluorescence

After transformation of chemically-competent BL21(DE3) cells, pre-cultures of at least three clones per case were launched overnight (ON) at 37°C and 200 rpm orbital-shaking until stationary phase/saturation. Precultures (2 μL) were seeded in Luria-Bertani broth (LB, 200 μL) supplemented with corresponding antibiotics in a 96-well glass-bottomed black plate (Greiner). IPTG (10 μM, final conc.) was added from the start, unless otherwise indicated in the text. Cells were incubated in a Clariostar Plus (BMG Labtech) at 37°C with shaking. Optical density (600 nm) and GFP fluorescence (excitation wavelength 470±15nm, emission at 515±20nm) were recorded every 10 min approximately for typically 16 h. Data were analyzed and fitted to a sigmoidal function with PRISM 6 (GraphPad). Plotted averages and standard deviations derived from at least two independent experiments, each one including several replicates per case (typically 3 clones, S9 Fig). Presented data were normalized with regard to the RMM reference values, in view that absolute fluorescence signals could vary considerably among independent experiments

### Protein expression

After transformation of chemically-competent BL21(DE3) of different constructs cloned in pET15b, pET26b, pET29b or pACYC vectors, single clones were pre-cultured ON in LB containing ampicillin (Amp, 100 μg/mL), Kan (40 μg/mL) or chloramphenicol (Chl, 40 μg/mL), respectively. In strategies combining two vectors, the next mixtures of antibiotics were present (concentration in μg/mL): Amp/Kan (50/25), Amp/Chl (50/25) or Kan/Chl (25/25). Pre-cultures were then diluted 100 fold in LB (2 to 10 mL, depending on experiment) implemented with due antibiotics at mentioned concentrations. IPTG induction (10 μM) was effected from the beginning and cultures were kept for 16 h at 37°C, 200 rpm (orbital shaker), unless otherwise indicated.

Cells were recovered by centrifugation at 6000g. After discarding the supernatant (SN), the pellet was lysed using Bugbuster Extraction Reagent (1/20 of culture volume, amine-free, Merck), supplemented with lysozyme (0.03 mg/mL final conc.), EDTA (1mM), Benzonase Nuclease (Merck, 268 U/mL final) and the protease-inhibitor phenylmethylsulfonyl fluoride (1mM). After incubation at room temperature (RT) for 10 min, solutions were stored on ice. Aliquots (40 μL) of total cellular contents were withdrawn, mixed with an equal volume of 2X loading dye before denaturation at 95°C for 10 min. The remaining volume was centrifuged at 21000 g for 10 min, at 4°C. The supernatant permitted to prepare aliquots of soluble fraction in the same manner as for total cellular contents.

Samples (1 to 3 μL) were analyzed by SDS-PAGE in gels (15 to 18%, 1:29 reticulation). Gel staining was either performed ON with Coomassie Brilliant Blue R-250 (Bio-Rad) or for 30 min with Instant Blue Coomassie Protein Stain (Abcam).

### Transmission electron microscopy

After ON induction as indicated for expression experiments, cell cultures (8 mL) were pre-fixed with an equivalent volume of glutaraldehyde (GA, 5%) and para-formaldehyde (PFA, 4%) in cacodylate buffer (100 mM, pH 7.2). After 15min at room temperature (RT), the cells were pelleted (6000 g) and resuspended in GA (2.5%)/PFA (2%) in the same cacodylate buffer. The solutions were kept for 2 h at RT, followed by 3 washings with the cacodylate buffer. Post-fixation was performed with 1% osmium tetroxide in the same buffer (1 h, RT). The cells were washed again 3 times with buffer and inlayed in low melting point agarose (2%) before uranyl acetate (1%) treatment (1 h, RT). Sample dehydration was performed using a gradient of ethanol: incubation in 25, 50, 70 and 90% ethanol for 15 min, plus 3 additional 30 min steps in 100% ethanol. Samples were transferred in Epon resin (EMS, Embed 812) with increasing concentration baths (25, 50, 75% in ethanol for 1 h at RT, then twice 2 h in 100% Epon at 37°C) and were finally embedded in Epon resin by a 48 h polymerization at 60°C.

80nm-thin sections prepared with the Ultramicrotome UCT (Leica) were mounted onto 200 mesh Formvar-carbon-coated copper grids and stained with Uranyless (EM-grade.com) and Reynolds lead citrate 3% (EM-grande.com). TEM acquisitions were made using a JEOL JEM-1400 at 80 kV and a digital camera Gatan Orius.

### *In vitro* tGFP reconstitution experiments

GFP1-9 was purified from BL21(DE3) cells transformed with the Receptor1 vector, where BMC-H coding sequences are missing in the first and second cassettes, and only expression of GFP1-9 can take place. A 500 mL of a culture was induced at mid-exponential growth with 200 μM IPTG, and left for 4 additional hours at 37°C. After lysis and centrifugation as indicated above, the supernatant was rapidly flowed through a TALON column (5 mL, Vivascience) that was previously equilibrated with a mixture of 300 mM NaCl, 10 mM sodium phosphate and 10 mM imidazole at pH 8.2 (Sol A). The resin was washed 3 times with 10 mL of Sol A conditioned at 4°C before elution with 3 mL of 300 mM NaCl, 10 mM sodium phosphate and 300 mM imidazole at pH 7.8 (Sol B), also kept at 4°C. EDTA (1 mM final) was added to the purified fraction immediately after elution. Purified proteins were dialyzed in 3500 Da MWCO Pur-A-Lyzer devices (Sigma-Aldrich) against 10 mM Tris, 200 mM NaCl, 1 mM EDTA buffer at pH 8 (300-fold volume exchange).

Soluble fractions containing GFP10/11-tagged RMM with either a long or short linker (in the absence of GFP1-9) were prepared from cell cultures (10 mL) as indicated above. IPTG induction (10 μM) was effected from the beginning and lasted ON. Pelleted cells were resuspended in SolA (2 mL) supplemented with lysozyme (0.05 mg/mL, final). This was followed by six cycles of 30 sec sonication at 25% amplitude, 4°C, were performed, which were spaced by 1 min lags (SO-VCX130 equipped with a 630–0422 probe, Sonics). After centrifugation (16000 g), the supernatant was collected. These soluble fractions (20 μL) were reacted for 2h at RT with the purified GFP1-9 (180 μL). Fluorescence signals were measured as indicated above after 2-fold dilutions with Sol A of the reaction mixtures.

### AlphaFold2 structural predictions

Query sequences were fed following the instructions provided online (ColabFold v1.5.2-patch: AlphaFold2 using MMseqs2), without permitting searches for structural templates. Five best ranked models were recovered for ulterior analysis. Structural images, analysis and RMSD calculations (S1 Fig and S1 Table) was performed with Pymol.

## Supporting information

S1 Table Prediction of protein oligomerization by AlphaFold 2. (PDF)

S1 Fig Evaluation of POI oligomerization potential. **A, Oligomeric structures predicted by Alphafold 2.** For each indicated POI, five models were generated based only on sequence information. For proteins presumed to be monomeric (left side), the input consisted of two concatenated identical amino acid sequences. For expected oligomers (right side), number of sequence repetitions was adapted to the expected oligomerization state. K1c/K1c and K1c/E1c coil-coil pairs were not considered, in virtue of difficulties to select an experimental structural reference. For each POI, the left image presents a view after superimposition of the AF2 top ranked model with a representative experimental structure, indicated in S1 Table. Only main-chain atoms from chains A were considered (cartoons, black color for the crystal structure, grey for rank1 model). The backbone of other chains are drawn as lines with indicated colors. Two spheres indicate the emplacement of N- (greys sphere) and C-terminal (blue) residues in the AF2 model. Models ranked second to fifth by AF2 are shown on the right view for each POI, after superimposing chain A backbone atoms to those from the best AF2 model. The latter is shown with chain A in grey, chain B in blue. Chains B from models 2 to 5 are colored as follows, respectively: green, cyan, magenta and limegreen. Please notice that, although the chains A were also colored with varied grey tonalities, their structures are too similar as to be discerned visually. Please see S1 Table for further details, also for estimations of RMSD values between pairs of structures. **B, Predicted alignment error (PAE) associated to AF2 models**. Shown are the plots provided by AF2 for the best ranked five models. Low confident interfaces are therefore predicted for all PIH-N, VHH and Smt3 generated dimers.
(TIF)

S2 Fig Evaluation of tGFP robustness in detecting PPIs with controls. **A, Fluorescence signals resulting from negative or positive PPI controls.** Cells were transformed with a single pET26b-based vector coding for indicated protein pairs and assayed for fluorescence reconstitution. Reported values are those at culturing times of 16 h, and are given as the percentage with regard to the value for the RMM pair reference (black bar). A single POI name is given when the same POI is fused to GFP10 and GFP11 tags, otherwise the two components are separated by a slash. The identity of the different protein pairs and PPI expectancy are detailed in the supplementary information file. **B, Pool-down effect of GFP1-9 on POI-10/11 partners.** After transformation of cells with pET26b-based plasmids permitting POI-10/11 co-expression in the presence (top) or absence (bottom) of GFP1-9, total cellular fractions after 16h of culture were analyzed by SDS-PAGE. White arrows point at bands from POI-10 partners, whereas black arrows identify POI-11 bands. The theoretical MW (kDa) of POI-10/POI-11 species are indicated below. The grey arrow notifies the GFP1-9 band.
(TIF)

S3 Fig Diminished detection of protein bands when POI-10 or POI-11 are co-expressed with GFP1-9. Protein expression levels were monitored by SDS-PAGE from BL21(DE3) transformed with single vectors, coding for separate tGFP partners as follows: pET26b coding for POI-10 on top, pACYC (11) on top for both GFP1-9 and POI-11, pET15b for POI-11, both in middle an, and pACYC (10) for GFP1-9 plus POI-10 in the middle. Shown are total contents of cells pelleted after 16 h cultures.
(TIF)

S4 Fig Impact of constitutive promoters with delayed GFP1-9 expression. **A, Fluorescence changes induced from replacement of T7p by constitutive promoters to control POI-10/11 bicistron transcription.** IPTG induction was effected from the beginning of the cultures. **B, Effect of delaying induction of GFP1-9**. Three cases are shown, representative of a strong CP promoter (J23106, left), of a weak CP promoter (J23103, middle) as well as the bicistron reference case with two T7p (right). Curve discontinuities are due to recomposition from separated datasets for technical reasons related to the need to stop monitoring to add the inductor at different times. The grey curve corresponds to background auto-fluorescence measured with cells carrying an empty pET26b vector.
(TIF)

S5 Fig His-tagged RMM with mutations on key peripheral residues still assemble. **A,** When expressed recombinantly in *E*. *coli*, wild-type His-tagged RMM hexamers form nanotubes, visible by TEM as honeycombs in transversal views (left and middle) and bundles in longitudinal sections (right). **B,** His-tagged RMM carrying mutations of hexamer peripheral residues still assembles giving rise to rolled layers with the sm-RMM (K26D) or even nanotubes with the dm-RMM (N29D/A53D). Two piled images are presented for each mutant.
(TIF)

S6 Fig TEM imaging of RMM-10/11 pairs co-expressed with GFP1-9. **A,** Wild-type RMM-10/11 pairs forms nanotubes when co-expressed recombinantly in *E*. *coli* with GFP1-9. Structures are however less well-defined than when studying the His-tagged RMM version. Honeycomb bundles reminiscent of those occurring with WT RMM are indicated with arrows. **B & C,** Assemblies might still occur in cells overexpressing combinations of sm-RMM-10/11 or dm-RMM-10/11 constructs, respectively, though structuration do not match to nanotubes or 2D-layers. **D,** Overexpression of tGFP partners based on the RMM triple mutant resulted in accumulation of proteins around cell poles, suggesting potential aggregation. Two images are presented for each mutant. **E, Analysis of soluble protein fractions by SDS-PAGE.** Gel-loaded material corresponds to soluble supernatants after centrifugation 21000 g of lyzed cells overexpressing WT RMM or mutant versions, in late phases of growth. Constructs carrying RMM in WT or mutants versions in fusion to His_6_ or GFP10/11 tags were analysed. All constructs studied in panels A-D, and on the right side of panel E, correspond to bicistronic single vectors with 30/27 linkers.
(TIF)

S7 Fig SUMO domain prevents macrostructure formation in tGFP screens. Observations by TEM of cells over-expressing different RMM-10/11 combinations carrying fusions to SUMO domains in N-terminus. The GFP10 and GFP11 are connected in the different constructs with Lk30/27 linkers. The GFP1-9 partner is also expressed with all cases.
(TIF)

S8 Fig Kinetics of nanotube formation in *E*. *coli*. Observations by TEM of cells over-expressing His_6_-tagged RMM at different culturing moments after induction, indicated on the left. Longitudinal and transverse views are provided for each time point. An increase of nanotube density is evidenced at longer growth times.
(TIF)

S9 Fig Example of fluorescence data acquisition and fits to a sigmoidal function. **A, Fluorescence raw data.** The RMM reference case is monitored under a bicistronic single vector configuration. Curves for each one of the 3 replicates (clones) are shown. **B, Average values and standard deviations**. Shown are averages of data from panel A for RMM, or similar data for other indicated cases. **C, Data fitting to sigmoidal function.** Non-linear regression to indicated function (shown at the bottom) permitted to extract the different parameters, namely F_max_ and the midpoint of fluorescence [t (half F_max_)]. **D, Comparison of raw data and best sigmoidal fits.**
(TIF)

S1 File Compilation of information pertaining molecular biology experimentation (including List S1-S4). (DOCX)

S2 File Complementary data supporting GFP1-9 pool down effect. (DOCX)

S3 File Original uncropped SDS-PAGE images that served to mount the figures of the manuscript. (PDF)
