# Supplementary material for: Assessment of oligomerization of bacterial micro-compartment shell components with the tripartite GFP reporter technology
Source: PLoS One. 2023 Nov 27;18(11):e0294760. doi: 10.1371/journal.pone.0294760 (PMC10681173; doi:10.1371/journal.pone.0294760)
Supplement: S1 Table — (PDF) [file pone.0294760.s001.pdf]

S1 Table - Prediction of protein oligomerization by AlphaFold 2.

POI	PDB	RMSD_A/RMSD_B (# atoms) after superposing A on A_rank1								
		Rank1[a]	Rank2		Rank3		Rank4		Rank5	
		ChA	ChA [b]	ChB [c]	ChA [b]	ChB [c]	ChA [b]	ChB [c]	ChA [b]	ChB [c]
**PIH-N**	4PSF	1.1 (536)	0.4	1.8	1.4	50	0.7	10	0.6	12
**VHH**	1XFP	0.9 (464)	1.3	15	0.6	5.5	0.4	12	0.3	20
**SUMO**	3QHT	0.8 (292)	0.4	49	0.4	19	0.4	15	0.4	8
**TrxA**	2TRX	0.4 (404)	0.1	1.2	0.1	0.9	0.1	1.6	0.1	2.2
**BwI**	3RDI	0.4 (268)	0.1	0.6	0.1	1.2	0.1	1.4	0.2	2.2
**Im9/E9**	2K5X	0.9 (848)	0.1	0.3	0.1	0.2	0.1	0.2	0.1	0.4
**ChorMut[d]**	1UFY	0.4 (452)	0.1	0.1	0.1	0.2	0.1	0.1	0.1	0.3
**CobTr[d]**	1WY1	1.0 (556)	0.2	0.3	0.2	0.2	0.2	0.3	0.1	0.2
**CutA[d]**	1NZA	0.5 (400)	0.1	0.1	0.1	0.1	0.1	0.1	0.1	0.1
**RMM[e]**	5L38	0.5 (324)	0.1	0.2	0.1	0.2	0.2	0.3	0.1	0.3

Five structural models were generated taking as input the repetition of two identical POI sequences, with exception of heterodimers, which consisted in one copy of each component. Template searches were not allowed. Generated models were relaxed using AMBER routines implemented within the AF2 algorithm. [a] The structure ranked first by AF2 was superimposed on the indicated crystal structure. Only main-chain atoms of residues belonging to chain A were considered, both for the superimposition and for the measurement of RMSD values presented in this column. Flexible portions in N- or C-terminus were excluded. Indicated within the parenthesis are the number of compared atom positions. [b] RMSD values after superimposition of each AF2-generated model with the structure ranked first by AF2, based on comparison of only main-chain atom positions of the same chain A core residues from the two compared structures. Again, residues on flexible or unstructured elements present at the N- or C-terminus were not taken into consideration. Compared core residues were: PIH-N (residues 1 to 140, 560 atoms); VHH (1 to 134, 536 atoms); SUMO (21 to 93, 292 atoms); TrxA (6 tp 106, 404 atoms); BwI (11 to 71, 244 atoms); Im9/E9 (residues 12 to 77 from Im9, 264 atoms; 8 to 132 from E9, 500 atoms); ChorMut (1 to 113, 452 atoms); CobTr (11 to 164, 616 atoms); CutA (1 to 101, 404 atoms); and RMM (4 to 89, 344 atoms). [C] After the superimposition based on chains A, the RMSD was estimated between core main-chain atoms of chains B, without attempting any structural alignment. [d] Repetitions of three identical sequences submitted to AF2, thus generating a trimer. [e] A combination of six monomers submitted to AF2. For the trimers and the RMM hexamer, the RMSD between atoms from chains other than A and B are not presented.
