# Supplementary figures and images for: Assessment of oligomerization of bacterial micro-compartment shell components with the tripartite GFP reporter technology
Source: PLoS One. 2023 Nov 27;18(11):e0294760. doi: 10.1371/journal.pone.0294760 (PMC10681173; doi:10.1371/journal.pone.0294760)

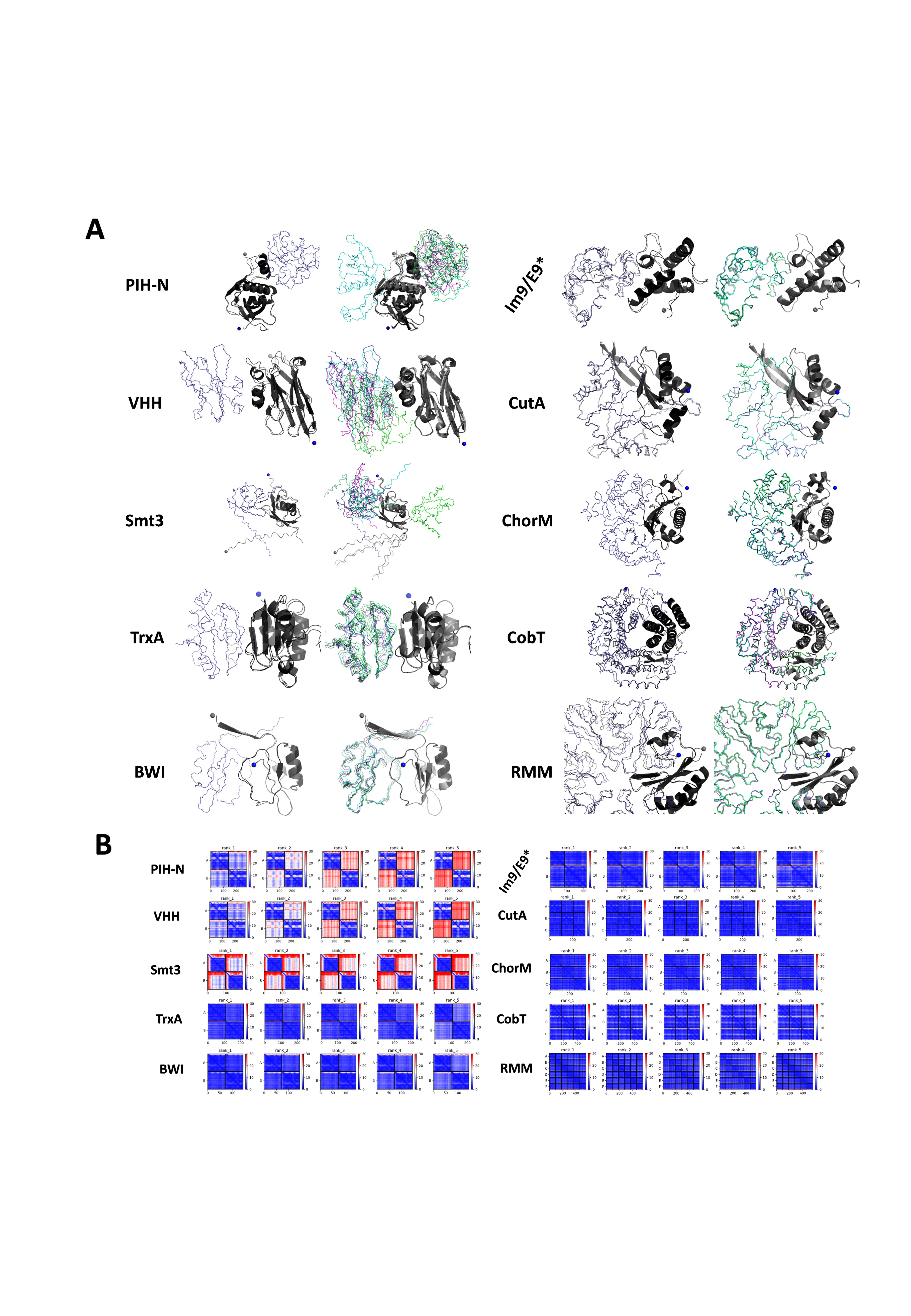

Supplement: S1 Fig — A, Oligomeric structures predicted by Alphafold 2. For each indicated POI, five models were generated based only on sequence information. For proteins presumed to be monomeric (left side), the input consisted of two concatenated identical amino acid sequences. For expected oligomers (right side), number of sequence repetitions was adapted to the expected oligomerization state. K1c/K1c and K1c/E1c coil-coil pairs were not considered, in virtue of difficulties to select an experimental structural reference. For each POI, the left image presents a view after superimposition of the AF2 top ranked model with a representative experimental structure, indicated in S1 Table. Only main-chain atoms from chains A were considered (cartoons, black color for the crystal structure, grey for rank1 model). The backbone of other chains are drawn as lines with indicated colors. Two spheres indicate the emplacement of N- (greys sphere) and C-terminal (blue) residues in the AF2 model. Models ranked second to fifth by AF2 are shown on the right view for each POI, after superimposing chain A backbone atoms to those from the best AF2 model. The latter is shown with chain A in grey, chain B in blue. Chains B from models 2 to 5 are colored as follows, respectively: green, cyan, magenta and limegreen. Please notice that, although the chains A were also colored with varied grey tonalities, their structures are too similar as to be discerned visually. Please see S1 Table for further details, also for estimations of RMSD values between pairs of structures. B, Predicted alignment error (PAE) associated to AF2 models. Shown are the plots provided by AF2 for the best ranked five models. Low confident interfaces are therefore predicted for all PIH-N, VHH and Smt3 generated dimers. (TIF) [file pone.0294760.s002.tif]

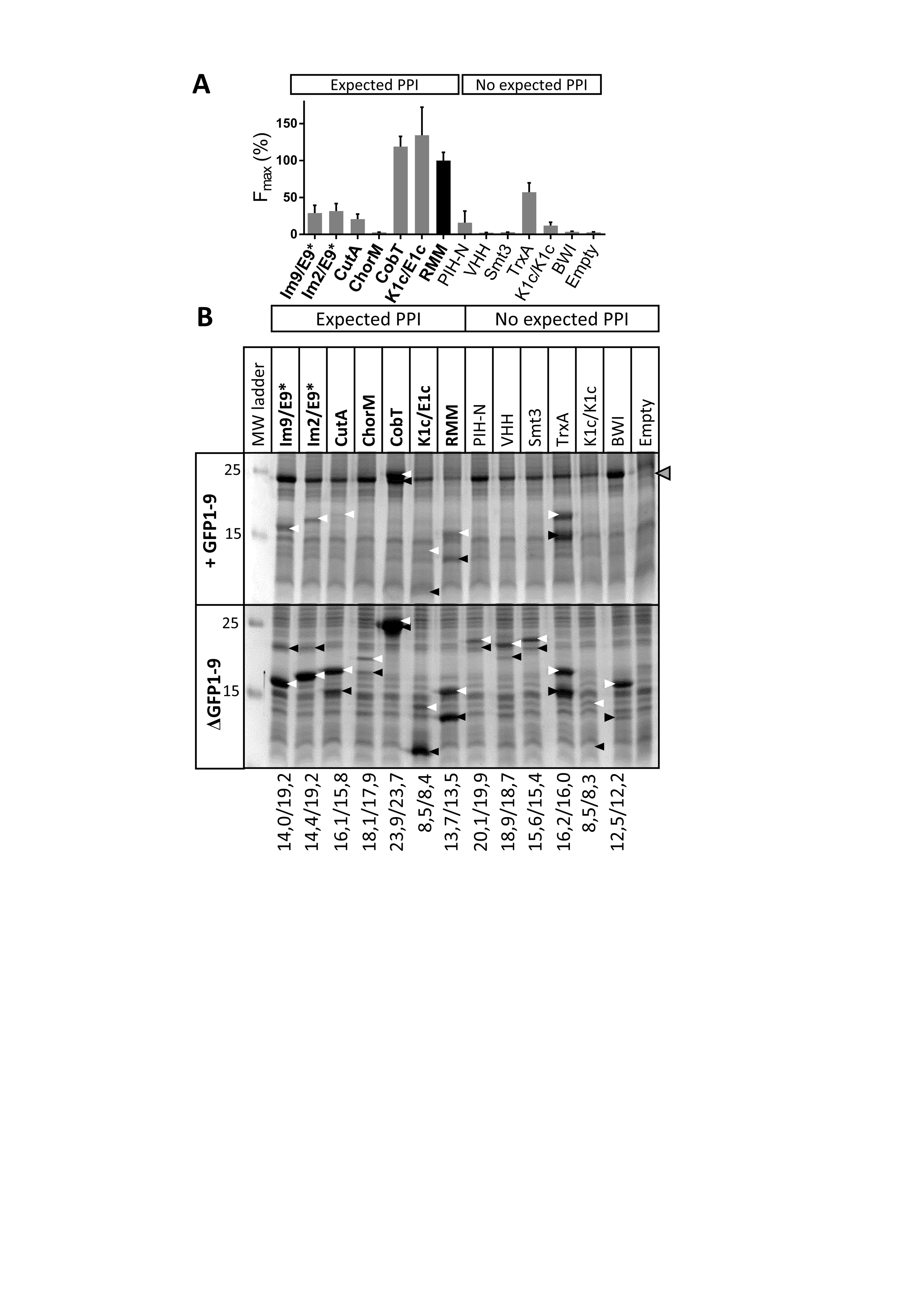

Supplement: S2 Fig — A, Fluorescence signals resulting from negative or positive PPI controls. Cells were transformed with a single pET26b-based vector coding for indicated protein pairs and assayed for fluorescence reconstitution. Reported values are those at culturing times of 16 h, and are given as the percentage with regard to the value for the RMM pair reference (black bar). A single POI name is given when the same POI is fused to GFP10 and GFP11 tags, otherwise the two components are separated by a slash. The identity of the different protein pairs and PPI expectancy are detailed in the supplementary information file. B, Pool-down effect of GFP1-9 on POI-10/11 partners. After transformation of cells with pET26b-based plasmids permitting POI-10/11 co-expression in the presence (top) or absence (bottom) of GFP1-9, total cellular fractions after 16h of culture were analyzed by SDS-PAGE. White arrows point at bands from POI-10 partners, whereas black arrows identify POI-11 bands. The theoretical MW (kDa) of POI-10/POI-11 species are indicated below. The grey arrow notifies the GFP1-9 band. (TIF) [file pone.0294760.s003.tif]

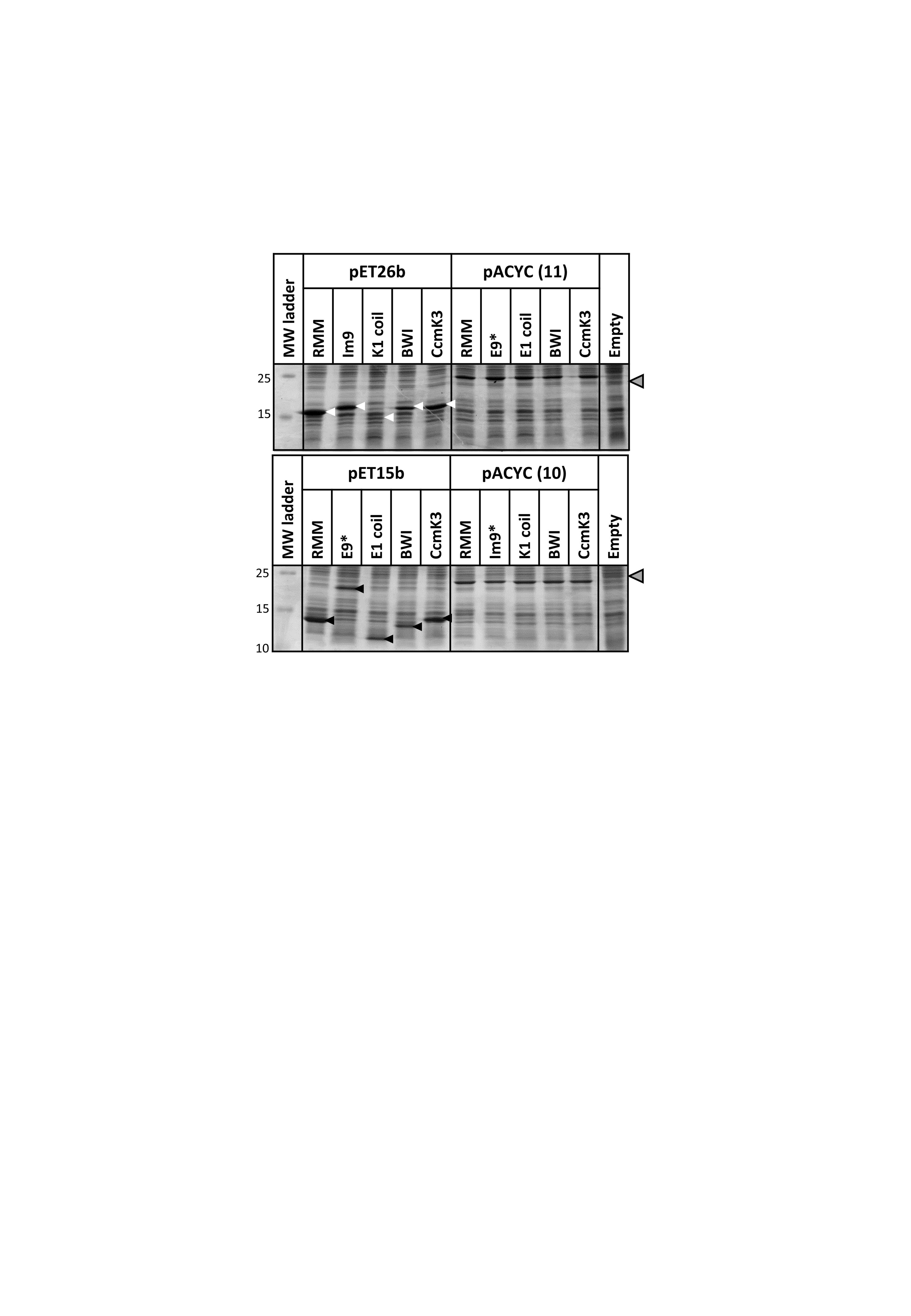

Supplement: S3 Fig — Protein expression levels were monitored by SDS-PAGE from BL21(DE3) transformed with single vectors, coding for separate tGFP partners as follows: pET26b coding for POI-10 on top, pACYC (11) on top for both GFP1-9 and POI-11, pET15b for POI-11, both in middle an, and pACYC (10) for GFP1-9 plus POI-10 in the middle. Shown are total contents of cells pelleted after 16 h cultures. (TIF) [file pone.0294760.s004.tif]

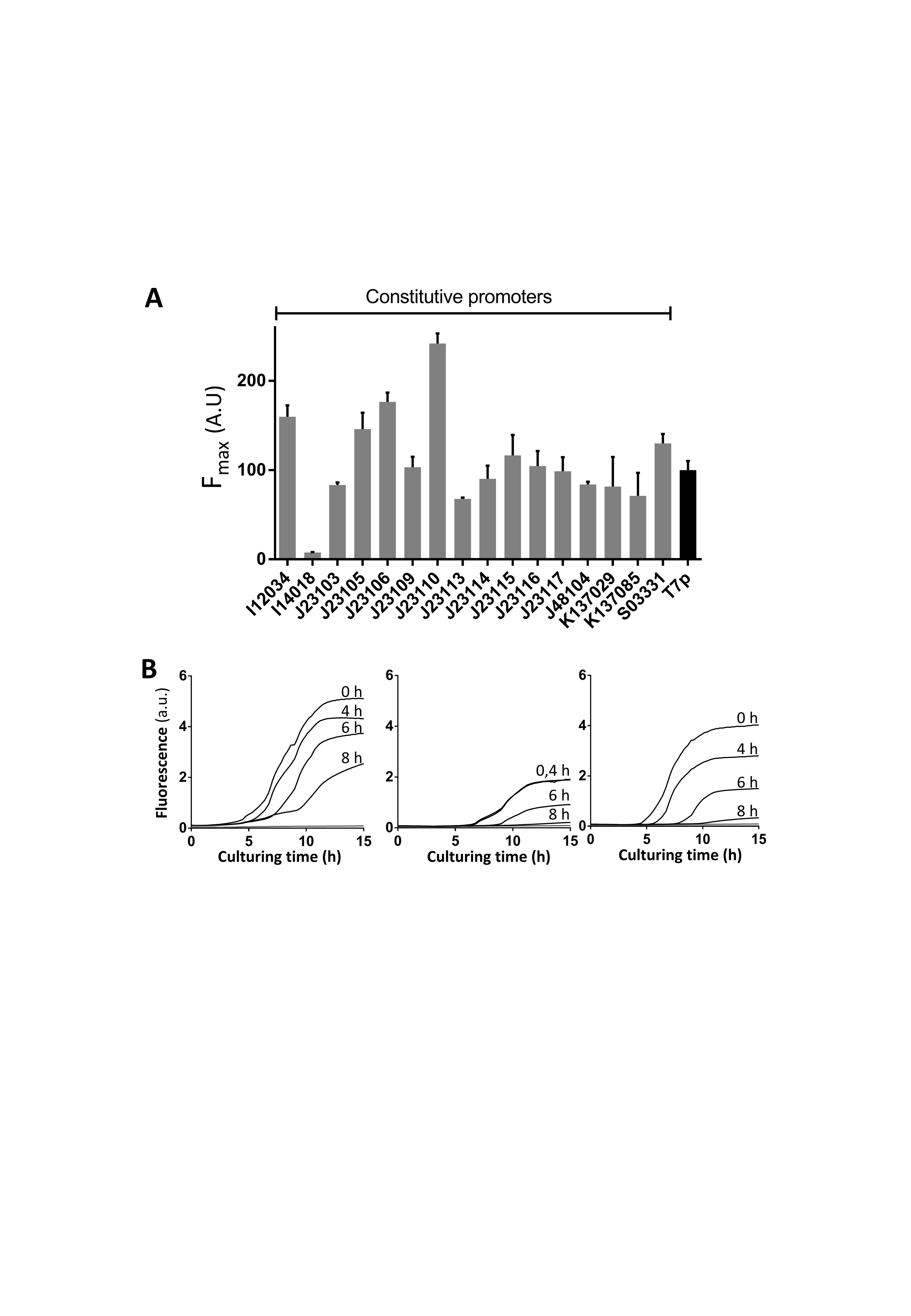

Supplement: S4 Fig — A, Fluorescence changes induced from replacement of T7p by constitutive promoters to control POI-10/11 bicistron transcription. IPTG induction was effected from the beginning of the cultures. B, Effect of delaying induction of GFP1-9. Three cases are shown, representative of a strong CP promoter (J23106, left), of a weak CP promoter (J23103, middle) as well as the bicistron reference case with two T7p (right). Curve discontinuities are due to recomposition from separated datasets for technical reasons related to the need to stop monitoring to add the inductor at different times. The grey curve corresponds to background auto-fluorescence measured with cells carrying an empty pET26b vector. (TIF) [file pone.0294760.s005.tif]

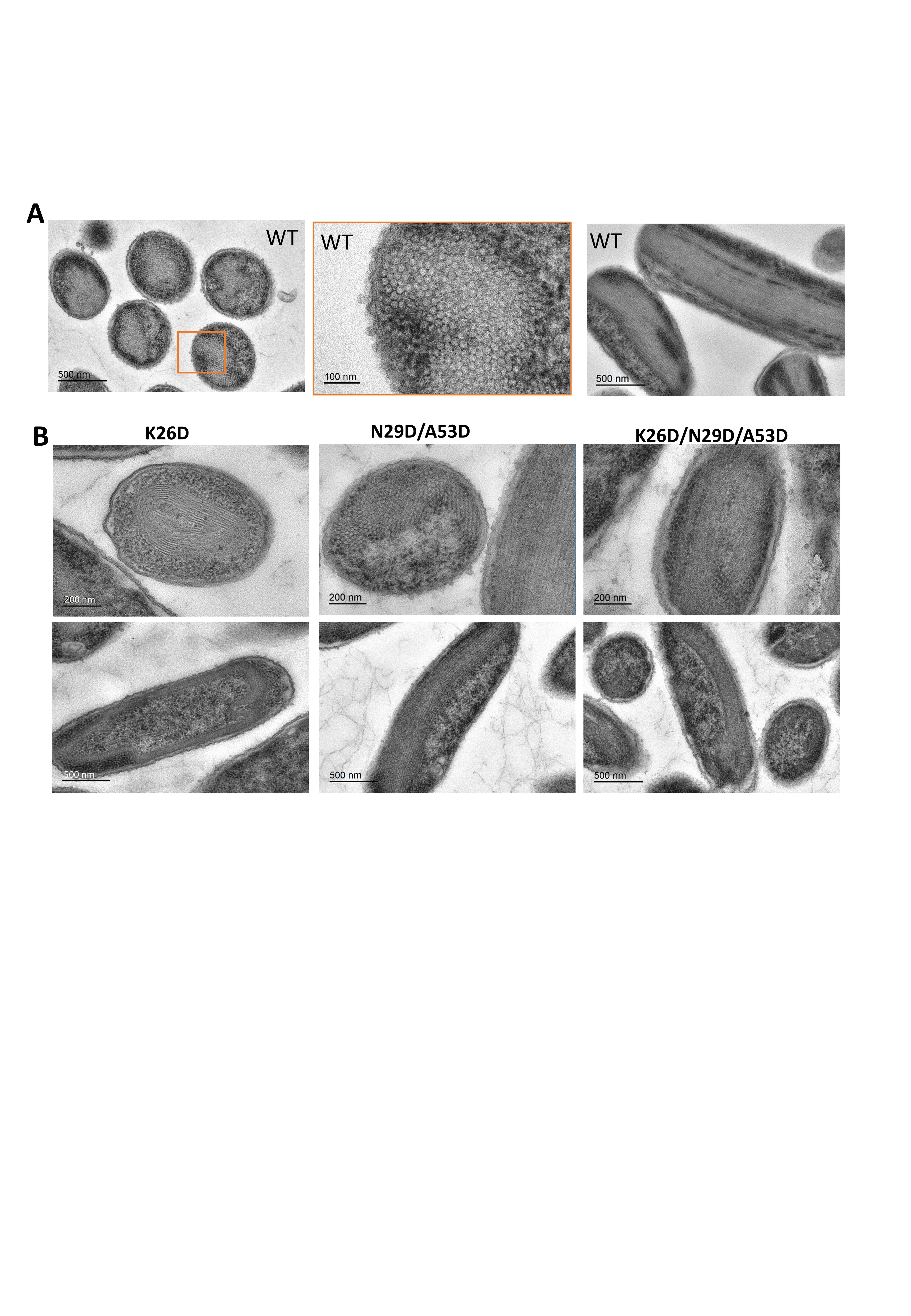

Supplement: S5 Fig — A, When expressed recombinantly in E. coli, wild-type His-tagged RMM hexamers form nanotubes, visible by TEM as honeycombs in transversal views (left and middle) and bundles in longitudinal sections (right). B, His-tagged RMM carrying mutations of hexamer peripheral residues still assembles giving rise to rolled layers with the sm-RMM (K26D) or even nanotubes with the dm-RMM (N29D/A53D). Two piled images are presented for each mutant. (TIF) [file pone.0294760.s006.tif]

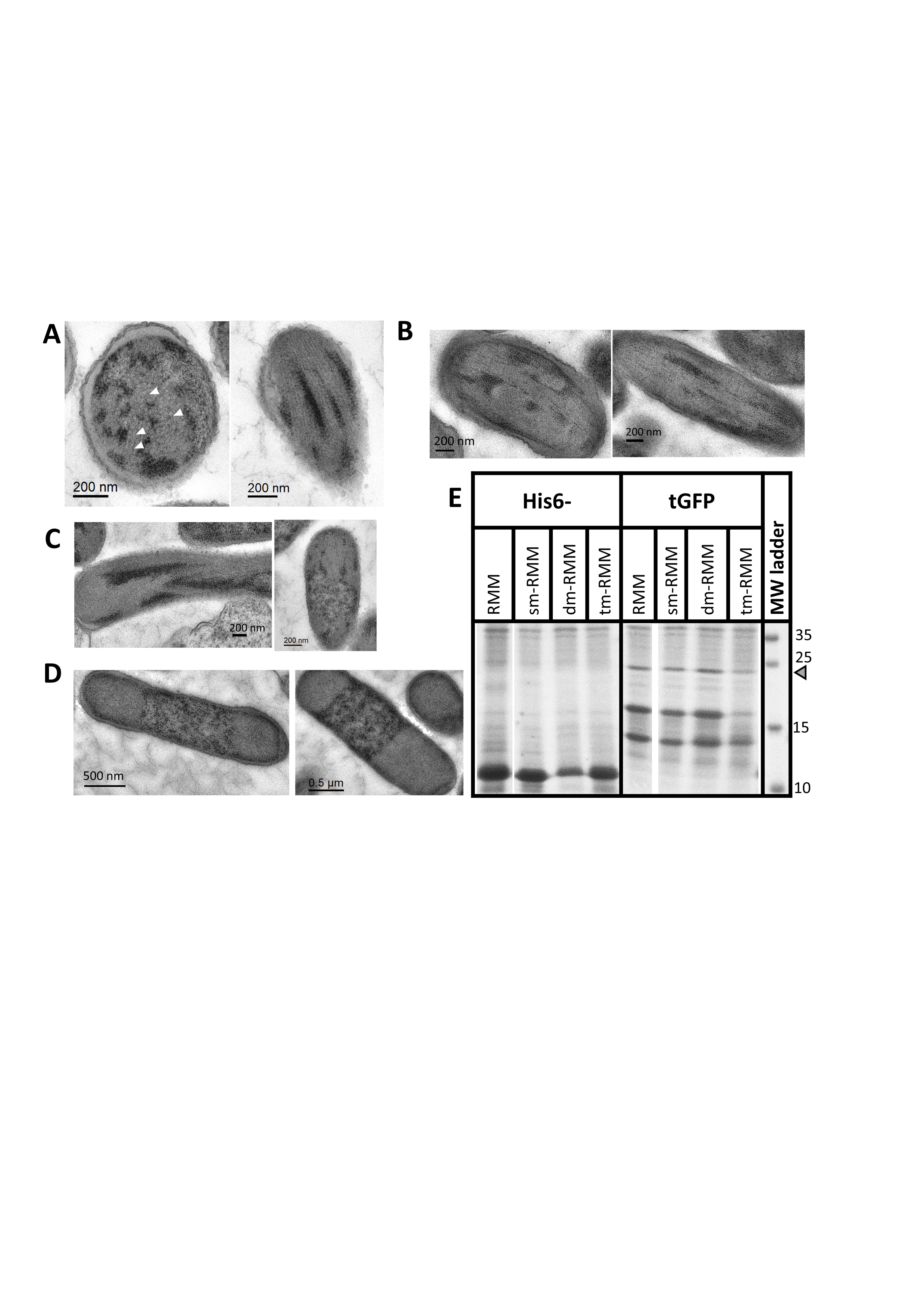

Supplement: S6 Fig — A, Wild-type RMM-10/11 pairs forms nanotubes when co-expressed recombinantly in E. coli with GFP1-9. Structures are however less well-defined than when studying the His-tagged RMM version. Honeycomb bundles reminiscent of those occurring with WT RMM are indicated with arrows. B & C, Assemblies might still occur in cells overexpressing combinations of sm-RMM-10/11 or dm-RMM-10/11 constructs, respectively, though structuration do not match to nanotubes or 2D-layers. D, Overexpression of tGFP partners based on the RMM triple mutant resulted in accumulation of proteins around cell poles, suggesting potential aggregation. Two images are presented for each mutant. E, Analysis of soluble protein fractions by SDS-PAGE. Gel-loaded material corresponds to soluble supernatants after centrifugation 21000 g of lyzed cells overexpressing WT RMM or mutant versions, in late phases of growth. Constructs carrying RMM in WT or mutants versions in fusion to His6 or GFP10/11 tags were analysed. All constructs studied in panels A-D, and on the right side of panel E, correspond to bicistronic single vectors with 30/27 linkers. (TIF) [file pone.0294760.s007.tif]

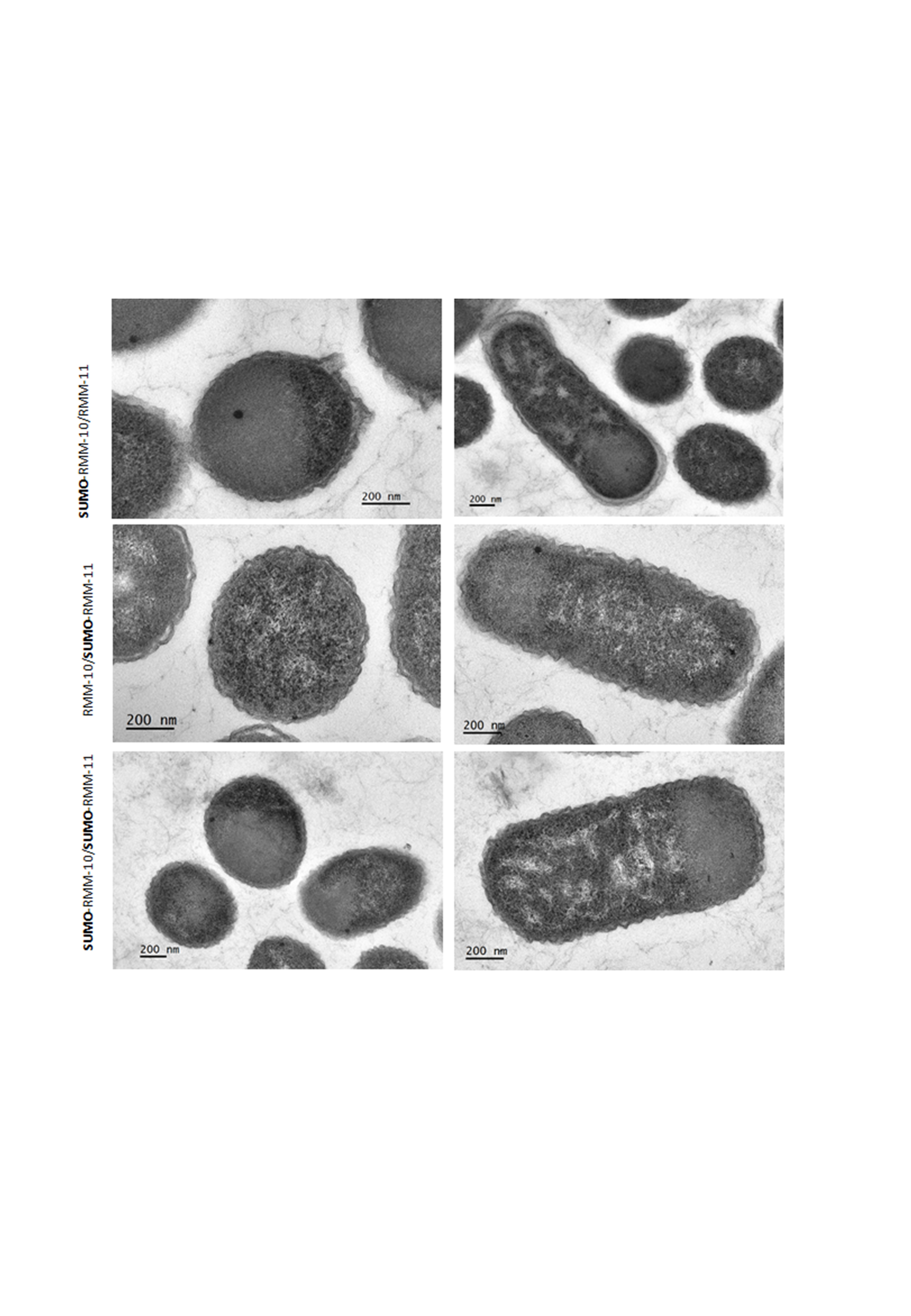

Supplement: S7 Fig — Observations by TEM of cells over-expressing different RMM-10/11 combinations carrying fusions to SUMO domains in N-terminus. The GFP10 and GFP11 are connected in the different constructs with Lk30/27 linkers. The GFP1-9 partner is also expressed with all cases. (TIF) [file pone.0294760.s008.tif]

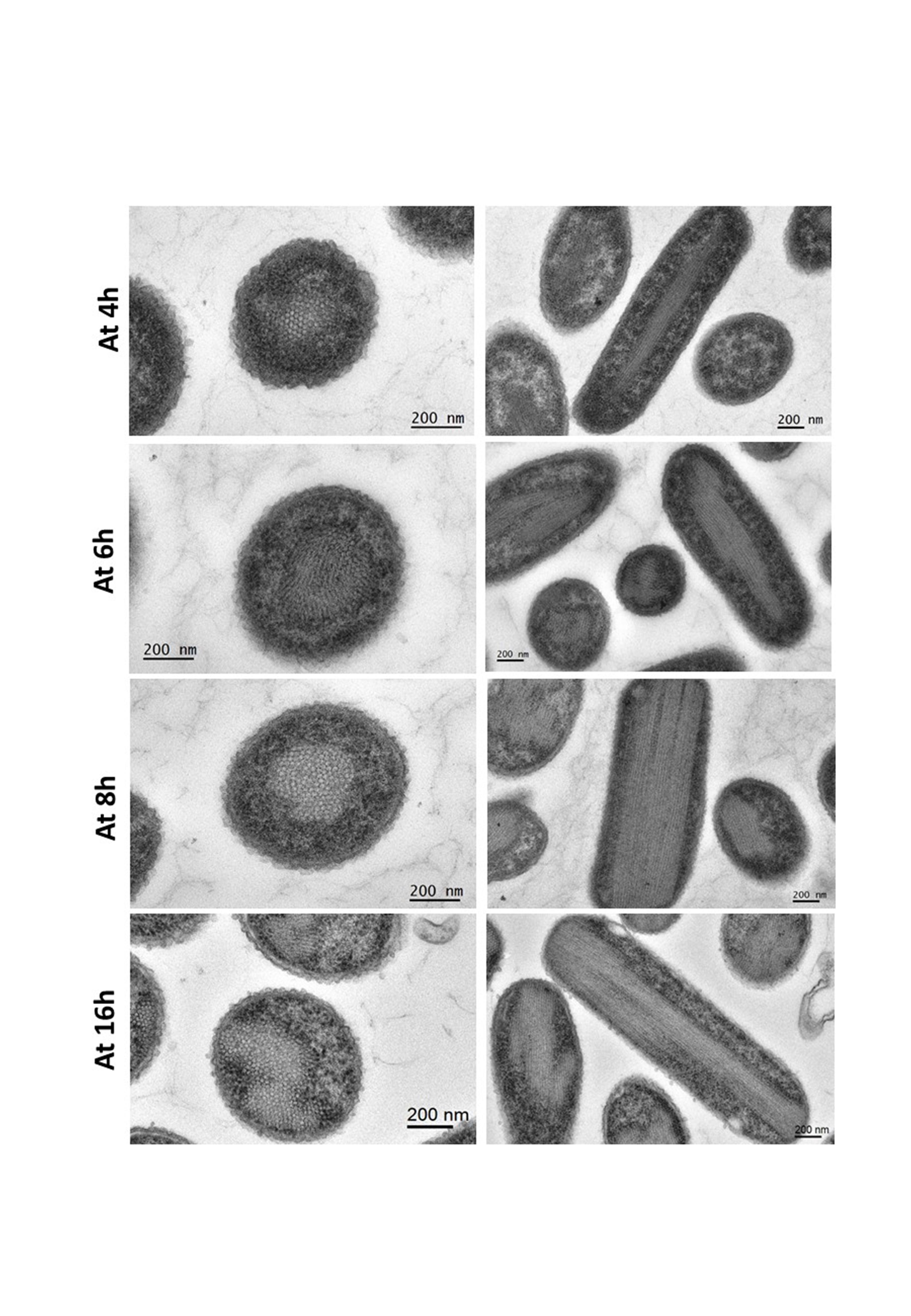

Supplement: S8 Fig — Observations by TEM of cells over-expressing His6-tagged RMM at different culturing moments after induction, indicated on the left. Longitudinal and transverse views are provided for each time point. An increase of nanotube density is evidenced at longer growth times. (TIF) [file pone.0294760.s009.tif]

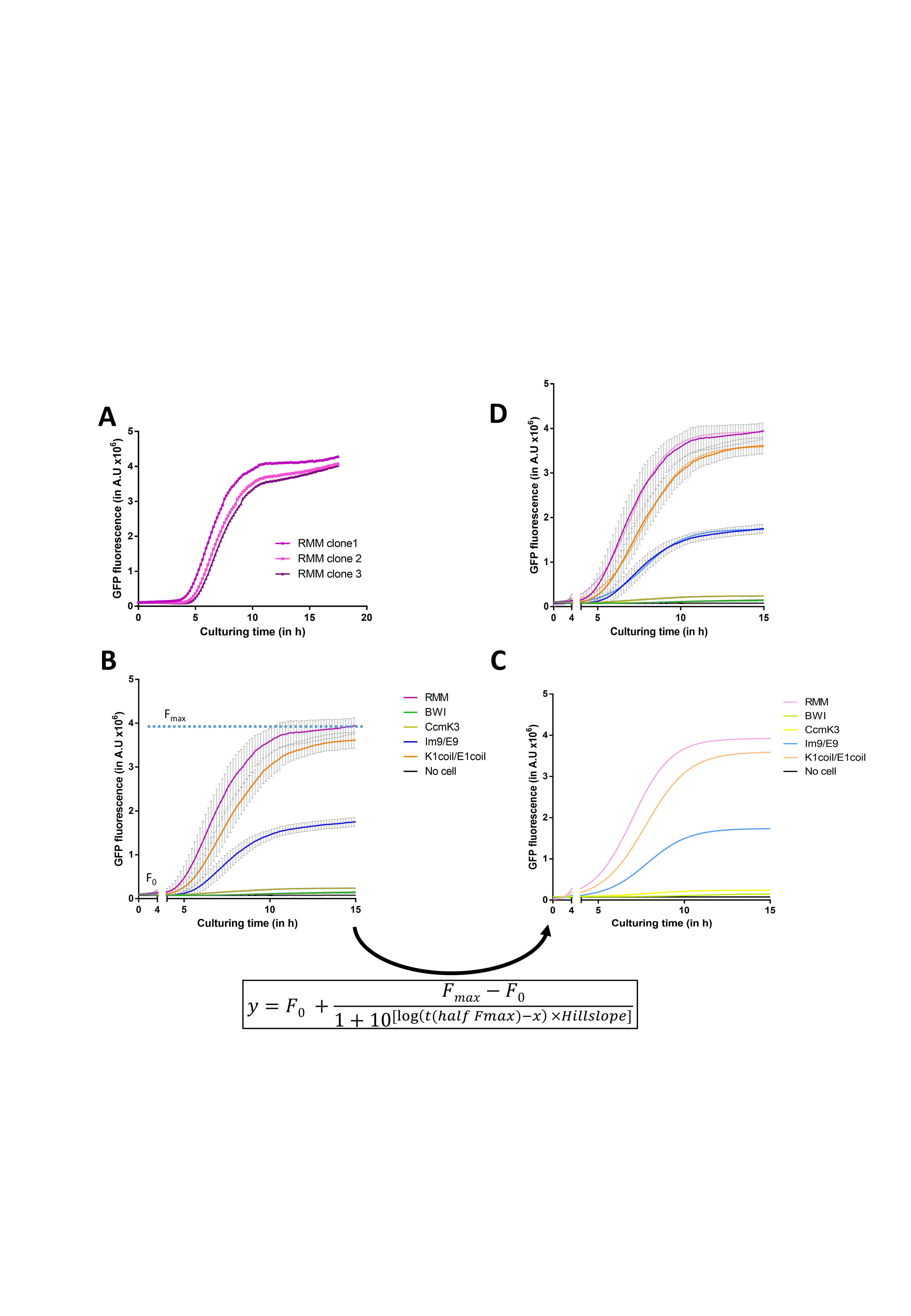

Supplement: S9 Fig — A, Fluorescence raw data. The RMM reference case is monitored under a bicistronic single vector configuration. Curves for each one of the 3 replicates (clones) are shown. B, Average values and standard deviations. Shown are averages of data from panel A for RMM, or similar data for other indicated cases. C, Data fitting to sigmoidal function. Non-linear regression to indicated function (shown at the bottom) permitted to extract the different parameters, namely Fmax and the midpoint of fluorescence [t (half Fmax)]. D, Comparison of raw data and best sigmoidal fits. (TIF) [file pone.0294760.s010.tif]
