# Supplementary material for: Assessment of oligomerization of bacterial micro-compartment shell components with the tripartite GFP reporter technology
Source: PLoS One. 2023 Nov 27;18(11):e0294760. doi: 10.1371/journal.pone.0294760 (PMC10681173; doi:10.1371/journal.pone.0294760)
Supplement: S1 File — (DOCX) [file pone.0294760.s011.docx]

**MATERIALS AND METHODS – Supplementary information:**

**Preparation of plasmid constructs :** The next picture schematizes the structure of DNA cassettes integrated in pET26b for single-vector strategies presented in the manuscript. Each partner of the tGFP is flanked by T7 promoters and terminators, giving rise to independent mRNA transcripts.


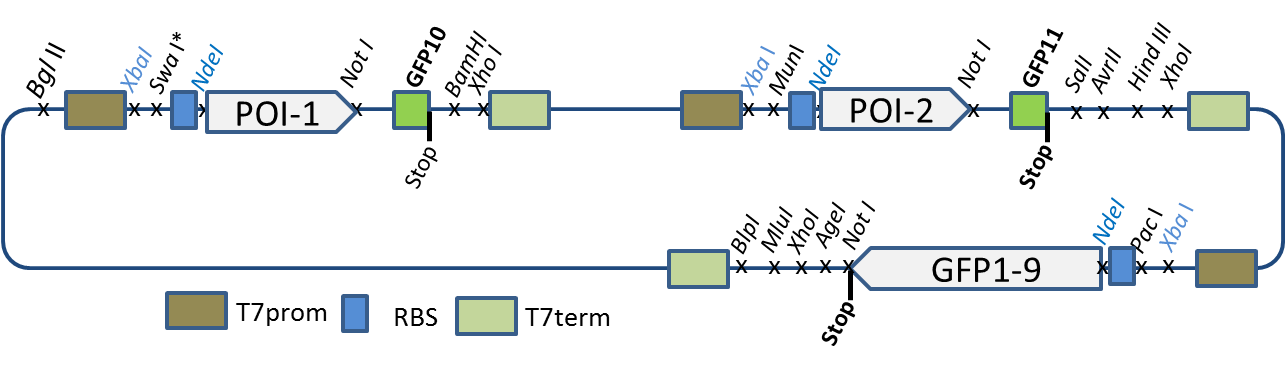


**List S1.** Next DNA sequences were integrated between indicated restriction sites to obtain different constructs studied in the main text. Important restriction sites are underlined, coding sequences in bold, GFP10 and GFP11 peptides in green and His-tags in red. Bicistronic and tricistronic versions were prepared from these tri-transcribed cases with BamHI/MunI and HindIII/PacI digestions as indicated in Materials and Methods.

| **Name** | **Nucleotide sequence** | **Organism** |
| --- | --- | --- |
| **Receptor1**  No POIs  +GFP1-9(His_6_)  (BglII/BlpI) | agatctcgatcccgcgaaattaatacgactcactataggggaattgtgagcggataacaattcccctctagaaataagatttaaatactttaagaaggagatatacatatggcttctggagtgcggccgcatcagaaggaggcggtagcgggggccctggttcgggaggggaaggttctgctgggggagggagcgctggcggggggtct**gatttaccagacgatcattacctgagcacacaaacgatcctttcgaaagacctgaac**gcaagctgataaggatccacttctcgagttaactcgtgagcaataactagcataaccccttggggcctctaaacgggtcttgaggggttttttgctgaaagtacacggccgcataatcgaaattaatacgactcactataggggaattgtgagcggataacaattcccctctagaaataattttacaattgtttaagaaggagatatacatatggattacagcggccgcaggcagcggtggcagcccgggcggcggcagcggcggcagcggcagcagcgcgagcggcggcagcaccagc**gaaaaacgcgatcacatggtgctgctggaatatgtgaccgcggcgggcattaccgatgcgagc**taatgacaagtatgtcgactcctaggaaagctttctcgagttaactcgtgagcaataactagcataaccccttggggcctctaaacgggtcttgaggggttttttgctgaaagtacacggccgcataatcgaaattaatacgactcactataggggaattgtgagcggataacaattcccctctagaattaattaagtttaactttaagaaggagatatacat**atgcgcaaaggcgaagaactgtttaccggcgtggtgccgattctgattgaactggatggcgatgtgaacggccataaattttttgtgcgcggcgaaggcgaaggcgatgcgaccattggcaaactgagcctgaaatttatttgcaccaccggcaaactgccggtgccgtggccgaccctggtgaccaccctgacctatggcgtgcagtgctttagccgctatccggatcacatgaaacgccatgatttttttaaaagcgcgatgccggaaggctatgtgcaggaacgcaccatttattttaaagatgatggcacctataaaacccgcgcggaagtgaaatttgaaggcgataccctggtgaaccgcattgaactgaaaggcattgattttaaagaagatggcaacattctgggccataaactggaatataactttaacagccataaagtgtatattaccgcggataaacagaacaacggcattaaagcgaactttaccattcgccataacgtggaagatggcagcgtgcagctggcggatcattatcagcagaacaccccgattggcgatggcccggtgctgctGCCGGATAACGGCAGCTCTGGTGCACATCACCATCACCATCAT**TAAGCGGCCGCACTTGTTACCggtcacctctcgagaaaacgcgtcgagagctgagc |  |
| Next POI sequences were ordered with indicated NdeI and NotI flanking sites. *In fine*, they were integrated in between NdeI/NotI sites of each shadowed box (cassette 1 or 2) from previous construct. Please notice, however, that the 1^st^ and 2^nd^ cassettes including GFP10 and GFP11 peptides, respectively, were prepared in intermediate single-ORF vectors, which were then transferred to the Receptor vector by treatments with SwaI/BamHI or MunI/SalI, as indicated in Materials and Methods. | | |
| RMM | CAT**aTGAGTAGTAACGCGATTGGTTTAATTGAAACGAAAGGATACGTCGCCGCACTGGCTGCTGCAGATGCTATGGTAAAAGCTGCAAATGTGACCATCACCGACCGGCAGCAGGTTGGCGATGGCTTAGTGGCAGTGATCGTAACGGGTGAGGTTGGGGCCGTAAAAGCTGCCACTGAAGCAGGCGCTGAAACTGCGTCGCAGGTTGGCGAGCTGGTTAGCGTGCATGTTATCCCACGTCCCCATTCGGAACTCGGCGCACATTTTAGCGTTAGCTCAAAAGGTGCggccGC** | *Rhodococ.* and *Mycobact.* |
| BWI | CAt**aTGGGATCTCTGCGTCAGTGCTCCGGTAAACAAGAATGGCCAGAGCTCGTTGGAGAGAGAGGGTCCAAGGCTGCCAAGATCATCGAAAACGAGAACGAAGACGTGCGAGCTATCGTCTTGCCTGAGGGTAGCGCGGTGCCTAGAGACCTCCGATGTGACCGTGTGTGGGTTTTCGTAGACGAGCGAGGAGTTGTTGTTGATACTCCTGTTGTTATGGGTGCggccGC** | *Fagopyrum esculentum* |
| sm-RMM | CAt**ATGAGTAGTAACGCGATTGGTTTAATTGAAACGAAAGGATACGTCGCCGCACTGGCTGCTGCAGATGCTATGGTAGATGCTGCAAATGTGACCATCACCGACCGGCAGCAGGTTGGCGATGGCTTAGTGGCAGTGATCGTAACGGGTGAGGTTGGGGCCGTAAAAGCTGCCACTGAAGCAGGCGCTGAAACTGCGTCGCAGGTTGGCGAGCTGGTTAGCGTGCATGTTATCCCACGTCCCCATTCGGAACTCGGCGCACATTTTAGCGTTAGCTCAAAAGGTGCGGCCGC** |  |
| dm-RMM | CAt**ATGAGTAGTAACGCGATTGGTTTAATTGAAACGAAAGGATACGTCGCCGCACTGGCTGCTGCAGATGCTATGGTAAAAGCTGCAGATGTGACCATCACCGACCGGCAGCAGGTTGGCGATGGCTTAGTGGCAGTGATCGTAACGGGTGAGGTTGGGGATGTAAAAGCTGCCACTGAAGCAGGCGCTGAAACTGCGTCGCAGGTTGGCGAGCTGGTTAGCGTGCATGTTATCCCACGTCCCCATTCGGAACTCGGCGCACATTTTAGCGTTAGCTCAAAAGGTGCGGCCGC** |  |
| tm-RMM | CAt**ATGAGTAGTAACGCGATTGGTTTAATTGAAACGAAAGGATACGTCGCCGCACTGGCTGCTGCAGATGCTATGGTAGATGCTGCAGATGTGACCATCACCGACCGGCAGCAGGTTGGCGATGGCTTAGTGGCAGTGATCGTAACGGGTGAGGTTGGGGATGTAAAAGCTGCCACTGAAGCAGGCGCTGAAACTGCGTCGCAGGTTGGCGAGCTGGTTAGCGTGCATGTTATCCCACGTCCCCATTCGGAACTCGGCGCACATTTTAGCGTTAGCTCAAAAGGTGCGGCCGC** |  |
| CcmK1 | CAt**ATGAGCATCGCTGTAGGTATGATCGAAACTCTGGGGTTTCCGGCTGTTGTGGAAGCAGCCGATAGCATGGTAAAAGCGGCGCGCGTGACCTTAGTGGGCTATGAAAAGATTGGCAGCGGTCGTGTCACCGTTATTGTTCGCGGGGATGTCAGCGAGGTGCAAGCGTCAGTGACGGCGGGTATCGAAAATATCCGTCGTGTAAACGGTGGAGAAGTACTGTCAAACCATATCATCGCACGCCCACATGAAAATCTGGAGTATGTTTTACCGATTCGCTATACGGAAGCTGTGGAGCAGTTTCGTGGTGCGGCCGC** | *Syn6803* |
| CcmK2 | cat**ATGAGTATCGCTGTGGGTATGATCGAAACACGCGGGTTTCCAGCGGTTGTGGAGGCGGCGGATTCAATGGTAAAAGCAGCGCGCGTTACCTTAGTGGGCTATGAAAAGATTGGCAGCGGTCGTGTAACCGTTATTGTGCGTGGGGATGTTAGCGAAGTCCAGGCAAGCGTCAGCGCCGGCATCGAGGCGGCAAATCGTGTGAATGGTGGGGAAGTACTGTCAACGCATATCATCGCACGCCCACATGAAAATCTGGAGTATGTTTTACCGATCCGTTATACgGGTGCGGCCGC** | *Syn6803* |
| CcmK3 | CAt**aTGGCACAAGCGGTGGGAGTGATTCAAACCTTGGGCTTTCCGAGCGTGTTAGCGGCGGCGGATGCGATGCTAAAAGGGGGCCGGGTGACGCTGGTGTATTATGACCTGGCTGAACGAGGCAACTTTGTAGTAGCAATCCGAGGTCCCGTATCAGAGGTTAACCTTTCGATGAAGATGGGATTAGCAGCGGTAAACGAGTCCGTCATGGGAGGTGAAATCGTTAGCCATTATATTGTGCCGAACCCGCCCGAAAATGTGCTGGCGGTTCTGCCAGTGGAGTATACCGAAAAGGTTGCTCGTTTCCGGACGGGTGCggccGC** | *Syn6803* |
| CcmK4 | cat**ATGTCCGCCCAGAGCGCCGTGGGCAGCATTGAAACCATTGGCTTTCCGGGCATTCTTGCCGCCGCGGATGCGATGGTAAAAGCTGGTCGCATTACCATTGTGGGCTATATTCGTGCGGGCTCTGCGCGCTTTACGCTGAACATTCGTGGGGATGTGCAGGAAGTTAAAACGGCGATGGCTGCGGGCATCGATGCCATCAACCGTACAGAAGGAGCCGATGTGAAAACCTGGGTCATTATTCCGCGCCCACATGAAAATGTCGTTGCGGTTCTGCCGATCGATTTTAGCGGTGCGGCCGC** | *Syn6803* |
| The next sequence was provided by Twist Biosciences directly integrated between NdeI to XhoI sites of pET29b (Kan). | | |
| SUMO-RMM | cat**ATGGGCGATTCAGAAGTGAACCAGGAGGCGAAACCAGAAGTTAAGCCGGAGGTGAAGCCGGAGACCCACATCAATCTAAAAGTAAGCGACGGCTCGTCGGAGATTTTCTTTAAGATTAAGAAAACAACCCCCCTGCGGCGTCTTATGGAGGCGTTTGCGAAGCGCCAAGGCAAGGAAATGGACTCACTTCGTTTCCTGTACGATGGTATTCGGATTCAGGCCGACCAGACACCGGAGGATTTGGATATGGAGGATAATGATATCATCGAGGCGCATCGTGAGCAGATTGGATCCATGAGTAGTAACGCGATTGGTTTAATTGAAACGAAAGGATACGTCGCCGCACTGGCTGCTGCAGATGCTATGGTAAAAGCTGCAAATGTGACCATCACCGACCGGCAGCAGGTTGGCGATGGCTTAGTGGCAGTGATCGTAACGGGTGAGGTTGGGGCCGTAAAAGCTGCCACTGAAGCAGGCGCTGAAACTGCGTCGCAGGTTGGCGAGCTGGTTAGCGTGCATGTTATCCCACGTCCCCATTCGGAACTCGGCGCACATTTTAGCGTTAGCTCAAAAGGATCCCTCGAG** |  |
| Next sequences from Twist Biosciences were cloned in a modified pACYC_Duet vector (see M&M) and served for the preparation of pACYC constructs, after exchanging 2^nd^ cassette POIs. | | |
| GFP1-9(His_6_)  +  RMM-10  (NcoI/BlpI) | cc**atgGCGCGCAAAGGCGAAGAACTGTTTACCGGCGTGGTGCCGATTCTGATTGAACTGGATGGCGATGTGAACGGCCATAAATTTTTTGTGCGCGGCGAAGGCGAAGGCGATGCGACCATTGGCAAACTGAGCCTGAAATTTATTTGCACCACCGGCAAACTGCCGGTGCCGTGGCCGACCCTGGTGACCACCCTGACCTATGGCGTGCAGTGCTTTAGCCGCTATCCGGATCACATGAAACGCCATGATTTTTTTAAAAGCGCGATGCCGGAAGGCTATGTGCAGGAACGCACCATTTATTTTAAAGATGATGGCACCTATAAAACCCGCGCGGAAGTGAAATTTGAAGGCGATACCCTGGTGAACCGCATTGAACTGAAAGGCATTGATTTTAAAGAAGATGGCAACATTCTGGGCCATAAACTGGAATATAACTTTAACAGCCATAAAGTGTATATTACCGCGGATAAACAGAACAACGGCATTAAAGCGAACTTTACCATTCGCCATAACGTGGAAGATGGCAGCGTGCAGCTGGCGGATCATTATCAGCAGAACACCCCGATTGGCGATGGCCCGGTGCTGCTGCCGGATAACGGCAGCTCTGGTGCACATCACCATCACCATCAT**TAAGCGTCGGCACTTGTTACCggtcacctctggagTTAACTCGTGAGCAATAACTAGCATAACCCCTTGGGGCCTCTAAACGGGTCTTGAGGGGTTTTTTGCTGAAAGTACACGGCCGCATAATCGAAATTAATACGACTCACTATAGGGGAATTGTGAGCGGATAACAATTCCCCTctagaaataagatttAAATACTTTAAGAAGGAGATATACAt**aTGAGTAGTAACGCGATTGGTTTAATTGAAACGAAAGGATACGTCGCCGCACTGGCTGCTGCAGATGCTATGGTAAAAGCTGCAAATGTGACCATCACCGACCGGCAGCAGGTTGGCGATGGCTTAGTGGCAGTGATCGTAACGGGTGAGGTTGGGGCCGTAAAAGCTGCCACTGAAGCAGGCGCTGAAACTGCGTCGCAGGTTGGCGAGCTGGTTAGCGTGCATGTTATCCCACGTCCCCATTCGGAACTCGGCGCACATTTTAGCGTTAGCTCAAAAGGTGCggccGCATCAGAAGGAGGCGGTAGCGGGGGCCCTGGTTCGGGAGGGGAAGGTTCTGCTGGGGGAGGGAGCGCTGGCGGGGGGTCTGATTTACCAGACGATCATTACCTGAGCACACAAACGATCCTTTCGAAAGACCTGAACGCAAGC**TGATAAGgatccacttctcgaGTTAACTAGCTGAGC |  |
| GFP1-9(His_6_)  +  RMM-11  (NcoI/BlpI) | cc**atgGCGCGCAAAGGCGAAGAACTGTTTACCGGCGTGGTGCCGATTCTGATTGAACTGGATGGCGATGTGAACGGCCATAAATTTTTTGTGCGCGGCGAAGGCGAAGGCGATGCGACCATTGGCAAACTGAGCCTGAAATTTATTTGCACCACCGGCAAACTGCCGGTGCCGTGGCCGACCCTGGTGACCACCCTGACCTATGGCGTGCAGTGCTTTAGCCGCTATCCGGATCACATGAAACGCCATGATTTTTTTAAAAGCGCGATGCCGGAAGGCTATGTGCAGGAACGCACCATTTATTTTAAAGATGATGGCACCTATAAAACCCGCGCGGAAGTGAAATTTGAAGGCGATACCCTGGTGAACCGCATTGAACTGAAAGGCATTGATTTTAAAGAAGATGGCAACATTCTGGGCCATAAACTGGAATATAACTTTAACAGCCATAAAGTGTATATTACCGCGGATAAACAGAACAACGGCATTAAAGCGAACTTTACCATTCGCCATAACGTGGAAGATGGCAGCGTGCAGCTGGCGGATCATTATCAGCAGAACACCCCGATTGGCGATGGCCCGGTGCTGCTGCCGGATAACGGCAGCTCTGGTGCACATCACCATCACCATCAT**TAAGCGTCGGCACTTGTTACCggtcacctctggagTTAACTCGTGAGCAATAACTAGCATAACCCCTTGGGGCCTCTAAACGGGTCTTGAGGGGTTTTTTGCTGAAAGTACACGGCCGCATAATCGAAATTAATACGACTCACTATAGGGGAATTGTGAGCGGATAACAATTCCCCTctagaaataattttacaattgtttaagaaggagatatacat**aTGAGTAGTAACGCGATTGGTTTAATTGAAACGAAAGGATACGTCGCCGCACTGGCTGCTGCAGATGCTATGGTAAAAGCTGCAAATGTGACCATCACCGACCGGCAGCAGGTTGGCGATGGCTTAGTGGCAGTGATCGTAACGGGTGAGGTTGGGGCCGTAAAAGCTGCCACTGAAGCAGGCGCTGAAACTGCGTCGCAGGTTGGCGAGCTGGTTAGCGTGCATGTTATCCCACGTCCCCATTCGGAACTCGGCGCACATTTTAGCGTTAGCTCAAAAGGTGCggccgcaggcagcggtggcagcccgggcggcggcagcggcggcagcggcagcagcgcgagcggcggcagcaccagcgaaaaacgcgatcacatggtgctgctggaatatgtgaccgcggcgggcattaccgatgcgagc**taatgacaagtatgtcgactcctaggaaagctttctcgaGTTAACTAGCtgagc |  |

**List S2.** Next sequences were assembled (Gibson) on a NdeI/SalI-opened **Receptor2** vector, identical to **Receptor1** but deprived of the NdeI (CATATG becomes CCTATG) and NotI sites (CGGGCCGC replaced by GCGGCAGC) that directly flank GFP1-9 sequence. Shadowed sequences represent the homology regions permitting assembly.

| SUMO-RMM-lk30-GFP10 | tactttaagaaggagatatacatATGGGCGATTCAGAAGTGAACCAGGAGGCGAAACCAGAAGTTAAGCCGGAGGTGAAGCCGGAGACCCACATCAATCTAAAAGTAAGCGACGGCTCGTCGGAGATTTTCTTTAAGATTAAGAAAACAACCCCCCTGCGGCGTCTTATGGAGGCGTTTGCGAAGCGCCAAGGCAAGGAAATGGACTCACTTCGTTTCCTGTACGATGGTATTCGGATTCAGGCCGACCAGACACCGGAGGATTTGGATATGGAGGATAATGATATCATCGAGGCGCATCGTGAGCAGATTGGATCCATGAGTAGTAACGCGATTGGTTTAATTGAAACGAAAGGATACGTCGCCGCACTGGCTGCTGCAGATGCTATGGTAAAAGCTGCAAATGTGACCATCACCGACCGGCAGCAGGTTGGCGATGGCTTAGTGGCAGTGATCGTAACGGGTGAGGTTGGGGCCGTAAAAGCTGCCACTGAAGCAGGCGCTGAAACTGCGTCGCAGGTTGGCGAGCTGGTTAGCGTGCATGTTATCCCACGTCCCCATTCGGAACTCGGCGCACATTTTAGCGTTAGCTCAAAAGGTGCGGCCGCATCAGAAGGAGGCGGTAGCGGGGGCCCTGGTTCGGGAGGGGAAGGTTCTGCTGGGGGAGGGAGCGCTGGCGGGGGGTCTGATTTACCAGACGATCATTACCTGAGCACACAAACGATCCTTTCGAAAGACCTGAACGCAAGCTGATAAggatcaattgtttaa |  |
| --- | --- | --- |
| SUMO-RMM-lk27-GFP11 | TAAggatcaattgtttaaGAAGGAGATATACCATGGGCGATTCAGAAGTGAACCAGGAGGCGAAACCAGAAGTTAAGCCGGAGGTGAAGCCGGAGACCCACATCAATCTAAAAGTAAGCGACGGCTCGTCGGAGATTTTCTTTAAGATTAAGAAAACAACCCCCCTGCGGCGTCTTATGGAGGCGTTTGCGAAGCGCCAAGGCAAGGAAATGGACTCACTTCGTTTCCTGTACGATGGTATTCGGATTCAGGCCGACCAGACACCGGAGGATTTGGATATGGAGGATAATGATATCATCGAGGCGCATCGTGAGCAGATTGGATCCATGAGTAGTAACGCGATTGGTTTAATTGAAACGAAAGGATACGTCGCCGCACTGGCTGCTGCAGATGCTATGGTAAAAGCTGCAAATGTGACCATCACCGACCGGCAGCAGGTTGGCGATGGCTTAGTGGCAGTGATCGTAACGGGTGAGGTTGGGGCCGTAAAAGCTGCCACTGAAGCAGGCGCTGAAACTGCGTCGCAGGTTGGCGAGCTGGTTAGCGTGCATGTTATCCCACGTCCCCATTCGGAACTCGGCGCACATTTTAGCGTTAGCTCAAAAGGATCCGCAGGCAGCGGTGGAAGTCCGGGTGGCGGTTCAGGCGGTAGCGGCAGCTCTGCGAGCGGCGGCAGCACCAGCGAAAAACGCGATCACATGGTGCTGCTGGAATATGTGACCGCGGCGGGCATTACCGATGCGAGCTAATGACAAGTATGtcgactcctaggaaagcttt |  |
| SUMO-RMM-lk1-GFP10 | tactttaagaaggagatatagCCATGGGCGATTCAGAAGTGAACCAGGAGGCGAAACCAGAAGTTAAGCCGGAGGTGAAGCCGGAGACCCACATCAATCTAAAAGTAAGCGACGGCTCGTCGGAGATTTTCTTTAAGATTAAGAAAACAACCCCCCTGCGGCGTCTTATGGAGGCGTTTGCGAAGCGCCAAGGCAAGGAAATGGACTCACTTCGTTTCCTGTACGATGGTATTCGGATTCAGGCCGACCAGACACCGGAGGATTTGGATATGGAGGATAATGATATCATCGAGGCGCATCGTGAGCAGATTGGtTCCaTGAGTAGTAACGCGATTGGTTTAATTGAAACGAAAGGATACGTCGCCGCACTGGCTGCTGCAGATGCTATGGTAAAAGCTGCAAATGTGACCATCACCGACCGGCAGCAGGTTGGCGATGGCTTAGTGGCAGTGATCGTAACGGGTGAGGTTGGGGCCGTAAAAGCTGCCACTGAAGCAGGCGCTGAAACTGCGTCGCAGGTTGGCGAGCTGGTTAGCGTGCATGTTATCCCACGTCCCCATTCGGAACTCGGCGCACATTTTAGCGTTAGCTCAAAAGGTGATTTACCAGACGATCATTACCTGAGCACACAAACGATCCTTTCGAAAGACCTGAACGCAAGCTGATAAggatcaattgtttaa |  |
| SUMO-RMM-lk1-GFP11 | TAAggatcaattgtttaagaaggagatatagCCATGGGCGATTCAGAAGTGAACCAGGAGGCGAAACCAGAAGTTAAGCCGGAGGTGAAGCCGGAGACCCACATCAATCTAAAAGTAAGCGACGGCTCGTCGGAGATTTTCTTTAAGATTAAGAAAACAACCCCCCTGCGGCGTCTTATGGAGGCGTTTGCGAAGCGCCAAGGCAAGGAAATGGACTCACTTCGTTTCCTGTACGATGGTATTCGGATTCAGGCCGACCAGACACCGGAGGATTTGGATATGGAGGATAATGATATCATCGAGGCGCATCGTGAGCAGATTGGATCCaTGAGTAGTAACGCGATTGGTTTAATTGAAACGAAAGGATACGTCGCCGCACTGGCTGCTGCAGATGCTATGGTAAAAGCTGCAAATGTGACCATCACCGACCGGCAGCAGGTTGGCGATGGCTTAGTGGCAGTGATCGTAACGGGTGAGGTTGGGGCCGTAAAAGCTGCCACTGAAGCAGGCGCTGAAACTGCGTCGCAGGTTGGCGAGCTGGTTAGCGTGCATGTTATCCCACGTCCCCATTCGGAACTCGGCGCACATTTTAGCGTTAGCTCAAAAGGTGAAAAACGCGATCACATGGTGCTgctggaatatgtgaccgcggcgggcattaccgatgcgagcTAATGACAAGTATGtcgactcctaggaaagcttt |  |
| EutM-GFP10 | tactttaagaaggagatatacatATGGAGGCCCTGGGAATGATCGAAACTCGCGGGCTGGTCGCCCTCATTGAGGCCTCAGACGCGATGGTAAAAGCAGCGCGGGTGAAGCTGGTTGGCGTTAAACAAATTGGTGGTGGtcTcTGCACAGCGATGGTACGTGGAGATGTAGCCGCATGCAAGGCGGCCACCGACGCGGGGGCGGCAGCGGCACAGCGGATTGGGGAATTAGTGAGCGTGCATGTTATTCCACGCCCTCATGGTGACCTGGAGGAAGTGTTTCCAATCGGTCTGAAGGGCGATTCCAGCAATCTGGGTGCGGCCGCATCAGAAGGAGGCGGTAGCGGGGGCCCTGGTTCGGGAGGGGAAGGTTCTGCTGGGGGAGGGAGCGCTGGCGGGGGGTCTGATTTACCAGACGATCATTACCTGAGCACACAAACGATCCTTTCGAAAGACCTGAACGCAAGCTGATAAggatcaattgtttaa | *E. coli K12* |
| EutM-GFP11 | taaggatcaattgtttaagaaggagatatacatATGGAGGCCCTGGGAATGATCGAAACTCGCGGGCTGGTCGCCCTCATTGAGGCCTCAGACGCGATGGTAAAAGCAGCGCGGGTGAAGCTGGTTGGCGTTAAACAAATTGGTGGTGGtcTcTGCACAGCGATGGTACGTGGAGATGTAGCCGCATGCAAGGCGGCCACCGACGCGGGGGCGGCAGCGGCACAGCGGATTGGGGAATTAGTGAGCGTGCATGTTATTCCACGCCCTCATGGTGACCTGGAGGAAGTGTTTCCAATCGGTCTGAAGGGCGATTCCAGCAATCTGGGTGCGGCCGCAGGCAGCGGTGGCAGCCCGGGCGGCGGCAGCGGCGGCAGCGGCAGCAGCGCGAGCGGCGGCAGCACCAGCGAAAAACGCGATCACATGGTGCTGCTGGAATATGTGACCGCGGCGGGCATTACCGATGCGAGCTAATGAcaagtatgtcgactcctaggaaagcttt |  |
| GFP10-CcmK4 | tactttaagaaggagatatacatATGGATTTACCAGACGATCATTACCTGAGCACACAAACGATCCTTTCGAAAGACCTGAACGGTGGGTCCGGCTCAGAAGGAGGCGGTAGCGGGGGCCCTGGTTCGGGAGGGGAAGGTTCTGCTGGGGGAGGGAGCGCTAGCGGCGGGTCCTCCGCCCAGAGCGCCGTGGGCAGCATTGAAACCATTGGCTTTCCGGGCATTCTTGCCGCCGCGGATGCGATGGTAAAAGCTGGTCGCATTACCATTGTGGGCTATATTCGTGCGGGCTCTGCGCGCTTTACGCTGAACATTCGTGGGGATGTGCAGGAAGTTAAAACGGCGATGGCTGCGGGCATCGATGCCATCAACCGTACAGAAGGAGCCGATGTGAAAACCTGGGTCATTATTCCGCGCCCACATGAAAATGTCGTTGCGGTTCTGCCGATCGATTTTAGCTAATGAGCGGCCGCggatcaattgtttaa | *Syn6803* |
| GFP11-CcmK4 | ggatcaattgtttaagaaggagatatacatATGGAAAAACGCGATCACATGGTGCTGCTGGAATATGTGACCGCGGCGGGCATTACCGATGCGAGCGGTGGGTCCGGCTCAGAAGGAGGCGGTAGCGGGGGCCCTGGTTCGGGAGGGGAAGGTTCTGCTGGTGGAGGGAGCGCTAGCGGCGCCTCCGCCCAGAGCGCCGTGGGCAGCATTGAAACCATTGGCTTTCCGGGCATTCTTGCCGCCGCGGATGCGATGGTAAAAGCTGGTCGCATTACCATTGTGGGCTATATTCGTGCGGGCTCTGCGCGCTTTACGCTGAACATTCGTGGGGATGTGCAGGAAGTTAAAACGGCGATGGCTGCGGGCATCGATGCCATCAACCGTACAGAAGGAGCCGATGTGAAAACCTGGGTCATTATTCCGCGCCCACATGAAAATGTCGTTGCGGTTCTGCCGATCGATTTTAGCTAATGAcaagtatgtcgactcctaggaaagcttt |  |
| All other cases indicated below were prepared similarly. The final sequence of N-terminally tagged cases (noted with an *) can be generated by replacing the stretch between NheI site and the STOP codon of CcmK4 sequences (underscored). Similarly, sequences of other C-terminally tagged cases can be obtained by introducing the indicated sequence between NdeI and NotI sites of EutM (underscored sites). | | |
| *CsoS1A | GCTAGCGGCGGGTCCGCCGACGTGACCGGGATCGCACTGGGAATGATCGAGACTCGTGGACTGGTCCCGGCTATCGAGGCCGCAGATGCAATGACAAAAGCTGCGGAGGTGCGTCTGGTAGGCCGGCAGTTTGTGGGCGGTGGTTATGTCACGGTTTTAGTGCGGGGTGAGACCGGGGCGGTAAACGCAGCGGTCCGTGCTGGTGCAGATGCTTGCGAACGGGTGGGTGATGGGCTGGTAGCGGCACATATCATCGCTCGTGTCCATTCTGAAGTTGAAAACATTCTGCCGAAGGCGCCACAGTAATGA | *H. neap.* |
| *RMM | GCTAGCGGCGGGTCCAGTAGTAACGCGATTGGTcTcATTGAAACGAAAGGATACGTCGCCGCACTGGCTGCTGCAGATGCTATGGTAAAAGCTGCAAATGTGACCATCACCGACCGGCAGCAGGTTGGCGATGGCTTAGTGGCAGTGATCGTAACGGGTGAGGTTGGGGCCGTAAAAGCTGCCACTGAAGCAGGCGCTGAAACTGCGTCGCAGGTTGGCGAGCTGGTTAGCGTGCATGTTATCCCACGTCCCCATTCGGAACTCGGCGCACATTTTAGCGTTAGCTCAAAATAATGA | *Rhodococ.* and *Mycobact.* |
| PduA | catATGCAGCAAGAAGCACTGGGAATGGTAGAAACTAAAGGGCTGACAGCGGCCATCGAGGCAGCAGATGCTATGGTAAAGAGCGCAAATGTTATGCTAGTGGGCTATGAAAAGATTGGCAGCGGtcTcGTGACTGTGATCGTACGTGGAGATGTAGGCGCAGTGAAGGCGGCCACCGACGCGGGGGCGGCAGCCGCACGTAATGTTGGTGAAGTAAAAGCTGTGCATGTGATCCCTCGTCCTCATACGGATGTGGAAAAGATTCTGCCGAAGGGTATCAGCCAGGGTGCGGCCGC | *Sal. typh. LT2* |
| HO BMC-H | CAtaTGGCTGACGCACTGGGAATGATCGAAGTACGTGGATTCGTCGGGATGGTAGAGGCCGCCGACGCGATGGTAAAAGCCGCGAAGGTGGAGCTGATTGGCTATGAAAAGACCGGCGGCGGCTATGTGACGGCGGTTGTGCGAGGTGACGTTGCTGCCGTAAAAGCTGCAACTGAAGCAGGCCAACGCGCGGCGGAGCGCGTTGGCGAGGTGGTGGCGGTGCATGTGATCCCACGTCCTCATGTCAACGTTGATGCCGCGTTGCCGCTTGGCCGGACCCCCGGTATGGACAAATCAGCGGGTGCggccGC | *H. ochraceum* |
| CsoS1D | catATGAACAACATTGATTTGAGAGTTTACTCTTTCATTGACTCTTTGCAACCACAATTaGCCTCTTACTTGGCTACTTCTTCTCAAGGTTTCTTGCCAGTTCCAGGTGACGCTTGTTTGTGGATTGAAGTTGCTCCAGGTATGGCTGTTCACAGATTGTCTGATATTGCTTTGAAGGCTACCAACGTTCGGTTAGGTGAACAAGTTGTTGAAAGAGCTTTCGGATCTATGGAAATTCACTACAGAAACCAATCTGACGTCTTGGCTTCTGGTGAGGCCGTTTTGAGAGAAATCAACCATGCTCAAGAAGATAGATTACCATGTAGAATCGCTTGGAAGGAGATCATCAGAGCTATTACTCCAGATCATGCCACCTTGATTAACAGACAATTAAGAAAGGGTTCCATGTTATTGCCTGGTAAATCAATGTTCATCTTGGAgACCGAACCAGCTGGTTACATTGTTCAAGCTGCCAACGAAGCCGAAAAAGCTGCACATGTTACTTTGATCGATGTTAGAGCCTTTGGTAACTTCGGTAGATTGACTATGATGGGTTCTGAAGCTGAAACTGAAGAAGCTATGAGAGCTGCTGAGGCAACTATTGCCTCCATTAATGCTAGAGCAAGAAGAGCTGAAGGTTTTGGTGCGGCCGC | *H. neap.* |
| CcmL | catATGCAGTTAGCGAAAGTTCTGGGAACGGTgGTcTCTACGTCAAAGACGCCTAACCTTACGGGAGTCAAGTTACTACTGGTACAGTTCCTAGATACGAAAGGTCAGCCGCTGGAGCGTTATGAAGTCGCGGGTGATGTAGTTGGCGCGGGCTTGAACGAATGGGTCCTGGTGGCCCGCGGTAGCGCGGCGCGCAAGGAACGTGGTAACGGTGATCGCCCACTGGATGCGATGGTAGTCGGTATCATCGATACAGTGAATGTTGCAAGCGGGAGCCTTTACAATAAAAGGGACGATGGGCGGGGTGCGGCCGC | *Syn6803* |
| CsoS4B | catATGGAAGTTATGCGTGTTCGTAGCGATCTGATTGCAACCCGTCGTATTCCGGGTCTcAAAAACATTAGCCTGCGTGTTATGGAAGATGCAACCGGcAAAGTTAGCGTTGCATGTGATCCGATTGGTGTTCCGGAAGGTTGTTGGGTTTTTACCATTAGCGGTAGCGCAGCACGTTTTGGTGTTGGTGATTTTGAAATTCTGACCGATCTGACCATTGGTGGCATTATTGATCATTGGGTTACaGGTGCGGCCGC | *H. neap.* |
| Im9 | catatggaactgaagcatagcattagtgattatacagaagctgaatttttacaacttgtaacaacaatttgtaatgcggacacttccagtgaagaagaactggttaaattggttacacactttgaggaaatgactgagcaccctagtggtagtgatttaatatattacccaaaagaaggtgatgatgactcaccttcaggtattgtaaacacagtaaaacaatggcgagccgctaacggtaagtcaggatttaaacagggcgcggccgc | *E. coli* |
| E9* | catATGGAGAGTAAACGGAATAAGCCAGGGAAGGCGACAGGTAAAGGTAAACCAGTTGGTGATAAATGGCTGGATGATGCAGGTAAAGATTCAGGAGCGCCAATCCCAGATCGCATTGCTGATAAGTTGCGTGATAAAGAGTTCAAAAGTTTCGACGATTTTCGGAAGGCTGTATGGGAAGAGGTGTCGAAAGATCCTGAGCTGAGCAAAAACTTGAACCCAAGCAATAAGTCCAGTGTTTCAAAAGGTTATTCTCCGTTTACTCCAAAGAATCAACAGGTCGGAGGGAGAAAAGTCTATGAACTTCATCATGACAAGCCAATTAGTCAAGGTGGTGAGGTTTATGACATGGATAATATCCGAGTGACTACACCTAAGCGAGCGATCGATATTCACCGAGGTAAGGGTGCggccgc | *E. coli* |
| K1 coil | CATATGAGCAAAGTATCCGCTTTAAAGGAAAACGTTTCTGCTCTCAAAGAGAAGGTCAGTGCTCTGACCGAAAAAGTGTCAGCCTTGAAGGAAAAAGTATCAGCACTTAAAGAAGGTGCGGCCGC |  |
| E1 coil | CATATGTCCAAAGTTTCCGCTTTAGAGAATGAAGTTTCTGCTCTCGAAAAAGAGGTCAGTGTCCTGGAAAAAGAGGTGTCAGCCTTGGAAAAGGAAGTACGTGCACTTGAGAAGGGTGCGGCCGC |  |
| PIH-N | CAtATGGCGGCGCATAGCGCGGCGCTGGAAGTGCTGTTTCAAGGCCCGGGTCAGCCGGGCTTTTGCATTAAAACCAACAGCAGCGAAGGCAAAGTGTTTATTAACATTTGCCATAGCCCGAGCATTCCGCCGCCGGCGGATGTGACCGAAGAAGAACTGCTGCAGATGCTGGAAGAAGATCAAGCGGGCTTTCGCATTCCGATGAGCCTGGGCGAACCGCATGCGGAACTGGATGCGAAAGGCCAAGGCTGCACCGCGTATGATGTGGCGGTGAATAGTGATTTTTATCGCCGCATGCAGAATAGCGATTTTCTGCGCGAACTGGTGATTACCATTGCGCGCGAAGGCCTGGAAGATAAATATAACCTGCAGCTGAACCCGGAATGGCGCATGATGAAAAACCGCCCGTTTATGGGCAGCATTGGTGCggccGC | *Homo sapiens* |
| VHHg | catatggcagatgtgcagctgcaggagtctgggggaggctcggtgcaggctggagggtctctgagactctcctgtacagcctctgaatatacttatagtgacctctgcatgggctggtaccgccaggctccagggcaggagcgtgagggggtcgcagctattagccgtgctggtactagcacatactacgtcgactccgtgaagggccgattcaccatctcccaggacaacgccaagaacacggtgtatctgcaaatgaacagcctgaaacctgaggacacggccatctattactgtgcagcagatgaggggcaggggtgtgacgcatacccaagcgactatattcggatggccggcaatgggtataactactggggccaggggacccaggtcaccgtctcctcaggtgcggccgc | *Camelus spp.* |
| Smt3 | catatgtccgatagcgaagtgaaccaggaggcgaaaccagaagtaaaacccgaagtaaaacccgaaacacacattaatttgaaagtaagcgacggctcgagcgagattttttttaagattaagaaaacgaccccactgcggcgtctgatggaggcctttgccaaacgtcagggtaaagagatggacagcttgcgtttcctgtacgatggtatccgtatccaggctgatcagacgccggaggatctggatatggaggataatgacattatcgaagcacatcgtgaacaaatcgggggtgcggccgc | *S. cerevisiae* |
| TrxA | catatgagcgataaaattattcacctgactgacgacagttttgacacggatgtactcaaagcggacggggcgatcctcgtcgatttctgggcagagtggtgcggtccgtgcaaaatgatcgccccgattctggatgaaatcgctgacgaatatcagggcaaactgaccgttgcaaaactgaacatcgatcaaaaccctggcactgcgccgaaatatggcatccgtggtatcccgactctgctgctgttcaaaaacggtgaagtggcggcaaccaaagtgggtgcactgtctaaaggtcagttgaaagagttcctcgacgctaacctggcaggtgcggccgc | *E. coli* |
| CobT | catatgcgtattacaaccaaggttggtgacaaaggctcgacacgcctgtttggtggggaggaagtctggaaagattccccaatcattgaggcaaacggcaccctggatgaactcacgagttttattggggaagccaagcactacgttgacgaggagatgaaagggatcctggaggaaattcaaaacgacatttacaagatcatgggggaaattggcagtaagggtaagatcgaaggcatcagtgaggagcgtatcaagtggctggaagggctgatttctcgctatgaagaaatggtcaatctgaagtcttttgtactgccagggggtactctggaaagtgctaagctggatgtatgccgtaccattgctcgccgtgccgaacgcaaggttgctacagtattacgtgaatttggtatcggtaaggaggcgctggtttacttgaatcggctgagtgatctgctgttcttgctggcacgcgttattgaaatcgaaaagaacaaactgaaggaggtccgttcaggtgcggccgc | *Pyr.horikoshii OT-3* |
| CutA | catatggaagaggtcgtgctgatcacggtgccgagcgaggaggtggcgcgtaccatcgccaaggccctggtggaggagcgcttggccgcctgcgtgaacatcgtccccggcctgacctccatctaccgctggcagggggaggtggtggaagaccaggagctgctgttgctggtcaagaccaccacccacgccttccctaagctgaaggaacgtgtcaaggccctccacccctacaccgtgcccgagatcgtggccctgcccatcgccgaggggaaccgtgagtacctggactggcttcgtgagaacacgggaggtgcggccgc | *T. thermoph.* |
| ChorM | catatggtccgtggcatccgcggcgccatcaccgtggaagaggacaccccggaggccatccaccaggccacccgcgaactgctcctgaagatgctggaggcgaacggcatccagagctacgaggagctggccgccgtcatcttcaccgtcaccgaggacctgacctccgccttccccgccgaggccgcgcgccagatcggcatgcaccgtgtgcctttactgtccgcccgcgaagtgccggtgccgggaagcctgccccgtgtgatccgcgtcttagccctgtggaacacggacaccccccaggaccgcgtgcgccacgtctacctgcgcgaggcggtgcgtctgcgtcccgacctggaaagcgcccagggtgcggccgc | *T. thermoph.* |

**List S3. Primers used to shorten linker between RMM and the GFP tags.**

| **Objective** | **Primer sequences** (5’-phosphorylated) |
| --- | --- |
| Lk1-GFP10 | 5’ GATTTACCAGACGATCATTACCTGAG  5’ ACCTTTTGAGCTAACGCTAAAATGTG |
| Lk4-GFP10 | 5’ GATTTACCAGACGATCATTACCTGAG  5’ TGCGGCCGCACCTTTTGA |
| Lk8-GFP10 | 5’ TCTGATTTACCAGACGATCATTACC  5’ TCCTTCTGATGCGGCCG |
| Lk12-GFP10 | 5’ TCTGATTTACCAGACGATCATTACC  5’ CCCGCTACCGCCTCCTTC |
| Lk18-GFP10 | 5’ TCTGATTTACCAGACGATCATTACC  5’ CCCTCCCGAACCAGGGC |
| Lk24-GFP10 | 5’ TCTGATTTACCAGACGATCATTACC  5’ TCCCCCAGCAGAACCTTCC |
| Lk1-GFP11 | 5’ GAAAAACGCGATCACATGGTGCT  5’ ACCTTTTGAGCTAACGCTAAAATGTG |
| Lk4-GFP11 | 5’ GAAAAACGCGATCACATGGTGCT  5’ TGCGGCCGCACCTTTTGA |
| Lk9-GFP11 | 5’ ACCAGCGAAAAACGCGATC  5’ ACCGCTGCCTGCGGC |
| Lk13-GFP11 | 5’ ACCAGCGAAAAACGCGATC  5’ GCCCGGGCTGCCACCG |
| Lk19-GFP11 | 5’ GGTAGTTCTGGCACCAGCGAAAAACGCG  5’ ACCAGAGCTACCGCTGCCACCGCTGCCT |
| **Sequencing primers (non phosphorylated)** | |
| Pr_For | 5’ CGGCGTAGAGGATCGAG |
| Pr_Rev | 5’ GGTAAACAGTTCTTCGCCTTTGC |
| **Modification of pACYC (5’-phosphorylated)** | |
| PrXb_For | Ttttcagtgcaatttatctcttcaaatgtag |
| PrXb_Rev | CTAGAAATATTTTATCTGATTAATAAGATGATCTTC |

**List S4. Primers to integrate constitutive promoters on bicistronic tGFP vectors.**

| **Promoter name** | **Nucleotide sequences** (5’-phosphorylated) |
| --- | --- |
| BBa_I12034 | 5’GATCTcgaCATTATTGCAATTAATAAACAACTAACGGACAATTCTACCTAACAaATTT  5’AAATtTGTTAGGTAGAATTGTCCGTTAGTTGTTTATTAATTGCAATAATGtcgA |
| BBa_I14018 | 5’GATCTcgaTGTAAGTTTATACATAGGCGAGTACTCTGTTATGGaATTT  5’AAATtCCATAACAGAGTACTCGCCTATGTATAAACTTACAtcgA |
| BBa_J23103 | 5’GATCTcgaCTGATAgctagctcagtcctaggGATTATgctagcaATTT  5’AAATtGCTAGCATAATCCCTAGGACTGAGCTAGCTATCAGtcgA |
| BBa_J23105 | 5’GATCTcgaTTTACGGCTAGCTCAGTCCTAGGTACTATGCTAGCaATTT  5’AAATtGCTAGCATAGTACCTAGGACTGAGCTAGCCGTAAAtcgA |
| BBa_J23106 | 5’GATCTcgaTTTACGgctagctcagtcctaggTATAGTgctagcaATTT  5’AAATtGCTAGCACTATACCTAGGACTGAGCTAGCCGTAAAtcgA |
| BBa_J23109 | 5’GATCTcgaTTTACAgctagctcagtcctaggGACTGTgctagcaATTT  5’AAATtGCTAGCACAGTCCCTAGGACTGAGCTAGCTGTAAAtcgA |
| BBa_J23110 | 5’GATCTcgaTTTACGgctagctcagtcctaggTACAATgctagcaATTT  5’AAATtGCTAGCATTGTACCTAGGACTGAGCTAGCCGTAAAtcgA |
| BBa_J23113 | 5’GATCTcgaCTGATGGCTAGCTCAGTCCTAGGGATTATGCTAGCaATTT  5’AAATtGCTAGCATAATCCCTAGGACTGAGCTAGCCATCAGtcgA |
| BBa_J23114 | 5’GATCTcgaTTTATGgctagctcagtcctaggTACAATgctagcaATTT  5’AAATtGCTAGCATTGTACCTAGGACTGAGCTAGCCATAAAtcgA |
| BBa_J23115^à^ | 5’GATCTcgaTTTATAGCTAGCTCAGTCCTTGGTACAATGCTAGCaATTT  5’AAATtGCTAGCATTGTACCAAGGACTGAGCTAGCTATAAAtcgA |
| BBa_J23116 | 5’GATCTcgaTTGACAgctagctcagtcctaggGACTATgctagcaATTT  5’AAATtGCTAGCATAGTCCCTAGGACTGAGCTAGCTGTCAAtcgA |
| BBa_J23117 | 5’GATCTcgaTTGACAGCTAGCTCAGTCCTAGGGATTGTGCTAGCaATTT  5’AAATtGCTAGCACAATCCCTAGGACTGAGCTAGCTGTCAAtcgA |
| BBa_J48104 | 5’GATCTcgataatcagtATGACGaatacttaaaatcgTCATACttatttaATTT  5’AAATtAAATAAGTATGACGATTTTAAGTATTCGTCATACTGATTAtcgA |
| BBa_K137029 | 5’GATCTcgaTTTAATtatatatatatatataTATAATggaagcgttttaATTT  5’AAATtAAAACGCTTCCATTATATATATATATATATATAATTAAAtcgA |
| BBa_K137085 | 5’GATCTcgaTTGACAatatatatatataTATAATgctagcaATTT  5’AAATtGCTAGCATTATATATATATATATATTGTCAAtcgA |
| BBa_S03331^à^ | 5’GATCTcgaTTGACAagcatttcctcagctccgtaaactaATTT  5’AAATtAGTTTACGGAGCTGAGGAAATGCTTGTCAAtcgA |

^à^ These primers have a point mutation (in red) compared to original sequence.
