# Supplementary material for: Assessment of oligomerization of bacterial micro-compartment shell components with the tripartite GFP reporter technology
Source: PLoS One. 2023 Nov 27;18(11):e0294760. doi: 10.1371/journal.pone.0294760 (PMC10681173; doi:10.1371/journal.pone.0294760)
Supplement: S2 File — (DOCX) [file pone.0294760.s012.docx]

**SUPPLEMENTARY INFORMATION**

**Evaluation of tGFP robustness**

The tGFP screening technology was challenged as means to characterize PPI. We chose for that several unrelated proteins of 8-25 kDa sizes, which were reported to be correctly expressed in soluble form in *E. coli*, and for which 3D structure and oligomerization properties are known. Two groups of proteins were considered, depending on whether an interaction (oligomerization) was expected or not. As PPI negative cases, in addition to BWI presented throughout the main text, we also screened pairs of GFP10/GFP11 partners based on the N-terminal PIH-N domain of human PIH1D1 protein that served Schneider and collaborators as scaffold to engineer robust mutable surfaces to create novel binders [1], and a well-expressed camelid VHH antibody that is used in our laboratory to detect *Syn6803* CcmK4. The thioredoxin A (TrxA) from *E. coli* and the Smt3 SUMO domain from *S. cerevisiae* were also included, as they are often exploited as means to augment expression yields and increase solubility of proteins in fusion [2]. As additional negative PPI control, we combined a pair based on the same positively charged K-coil peptide (K1c/K1c), engineered by Tripet et al. [3]. As presented in the main text, a combination of the K1-coil with the anionic E1 coil (K1c/E1c) from the same study and the combinations of *E. coli* colicin E9* and its cognate Im9 partner or the Im2 binder [4] were taken as positive PPI cases. E9* corresponds to E9 DNase inactivated by mutation of the active site His575 into alanine to prevent potential toxicity caused by possible stoichiometric imbalance of E9/Im9 levels. Other positive PPI cases included were the trimeric copper tolerance protein (CutA) and chorismate mutase (ChorM) from *T. thermophilus [5]*, as well as the cobalamin adenosyltransferase (CobT) from *Pyrococcus horikoshii OT-3*. Results were compared to the RMM reference case.

For these studies, GFP10 or GFP11 strands were connected with 27 or 30 residue-long flexible linkers. After transformation of BL21(DE3) cells with plasmids permitting the co-expression of POI-10, POI-11 partners and GFP1-9 from independent cassettes, cellular growth (600 nm) and GFP fluorescence emission were monitored overnight, with 10 µM IPTG added from the beginning of the incubation. No significant differences of bacterial growth were noticed when comparing different POI cases. On the contrary, fluorescence curves varied significantly among studied cases (Fig. S2A). Globally, signals were close to background auto-fluorescence for expected negative PPI combinations BWI/BWI, PIH-N/PIH-N, VHH/VHH, K1c/K1c and Smt3/Smt3, whereas strong signals were reached for positive PPI interactors combining K1c/E1c, CobT or RMM pairs. The combinations with E9 or CutA were however less fluorescent than expected. The two clearer outliers were the combination based on the supposedly monomeric TrxA protein, which induced strong fluorescence, and the ChorM pair, which did not show any sign of trimerization.

**Reassessment of oligomerization trends with Alphafold2**

The attribution of oligomerization trends of screened POI was inspected with Alphafold2 (AF2), an algorithm that can be successfully applied to predict PPI occurrence [6]. Thus, 2 to 6 concatenated sequences were submitted to AF2 searches in the absence of structural templates. A comparison of protein structures of the top rank solution (out of the five generated models) with available experimental structures confirmed the excellent performance of AF2 algorithm (Fig. S1A, see also Table S1). The calculated root-mean-square-deviations (RMSD) for main-chain atom positions differed by only 0.4 to 1.1 Å. Next, the main-chain atom position of chain A from each proposed model were structurally aligned to the same atom positions from the top solution. The low RMSD calculated between these chain A atoms proved that the tertiary protein structures were practically identical among models. However, quaternary structures differed when comparing the five PIH-N, VHH and SUMO homo-dimer models, pointing to the absence of an evolved interface to sustain dimerization. This was also evidenced by the high values in the predicted alignment error (PAE) matrices between residues from different monomers (see. Fig. S1B). On the contrary, practically identical oligomers were generated for all other cases, including the presumed monomeric BWI or TrxA. As mentioned in the main text, the C-terminal atom of BWI occurred at the dimer interface, suggesting that the potential dimer might be perturbed by GFP10/11 tagging.

**Confirming GFP1-9 pull-down effect**

Protein expression was profiled by SDS-PAGE from total cells contents pelleted at late culture stages (Fig. S2B). Faint bands were noticed for most cases, with often at least one of the two POI partners being undetected. This was notorious with CutA and ChorM and cases expected to be negative. On the contrary, bands for the two TrxA-10/11 species were intense. A strong accumulation of this protein might therefore cause tGFP reconstitution following random collisions inside the cytoplasm.

Bearing in mind data presented in the main text that evidenced a pool-down effect caused by GFP1-9, we evaluated cellular POI accumulation in the absence of GFP1-9. As shown in Fig. S2B, deletion of the GFP1-9 coding sequence again caused a neat increase of SDS-PAGE band intensities for most cases, with notable improvements for several cases. Only TrxA and CobT expression remained unaltered, something that might be related to their known high solubility. These data therefore corroborated that GFP1-9 presence causes a deleterious effect on co-expressed potential partners.

**Pull-down effect is not related to the presence of water-exposed hydrophobic residues**

Here, we explored the possibility that POI-10 and especially POI-11 species could be less soluble as a consequence of the presence of a patch of four hydrophobic residues (Ile206, Val219, Leu221 and Tyr223) that would cluster in the water exposed side of reconstituted tGFP. The first and last three residues lye in the GFP10- and GFP11-strands, respectively. These residues are conserved among GFP variants, but not in other structurally-related fluorescent proteins. Therefore, we replaced them by more hydrophilic residues. Thus, single I206A and I206K GFP10 mutants, or the L221K, Y223H and L221E/Y223H mutants of GFP11 were mounted in combinations of RMM and BWI pairs.

The maximal fluorescence measured for RMM pairs declined in the following manner (in parenthesis the signal proportion with regard to the original tGFP setup): L221K (42%), Y223H (37%), L221E/Y223H (28%), I206K (17%) and I206A (9%). No improvement was noticed neither in terms of signal ratios with regard to BWI.

**REFERENCES**

1. Pham PN, Huličiak M, Biedermannová L, Černý J, Charnavets T, Fuertes G, et al. Protein Binder (ProBi) as a New Class of Structurally Robust Non-Antibody Protein Scaffold for Directed Evolution. Viruses. 2021;13(2). Epub 20210127. doi: 10.3390/v13020190. PubMed PMID: 33514045; PubMed Central PMCID: PMCPMC7911045.

2. Young CL, Britton ZT, Robinson AS. Recombinant protein expression and purification: a comprehensive review of affinity tags and microbial applications. Biotechnol J. 2012;7(5):620-34. Epub 20120110. doi: 10.1002/biot.201100155. PubMed PMID: 22442034.

3. Tripet B, Yu L, Bautista DL, Wong WY, Irvin RT, Hodges RS. Engineering a de novo-designed coiled-coil heterodimerization domain off the rapid detection, purification and characterization of recombinantly expressed peptides and proteins. Protein Eng. 1996;9(11):1029-42. doi: 10.1093/protein/9.11.1029. PubMed PMID: 8961356.

4. Garinot-Schneider C, Pommer AJ, Moore GR, Kleanthous C, James R. Identification of putative active-site residues in the DNase domain of colicin E9 by random mutagenesis. J Mol Biol. 1996;260(5):731-42. doi: 10.1006/jmbi.1996.0433. PubMed PMID: 8709151.

5. Bagautdinov B. The structures of the CutA1 proteins from Thermus thermophilus and Pyrococcus horikoshii: characterization of metal-binding sites and metal-induced assembly. Acta Crystallogr F Struct Biol Commun. 2014;70(Pt 4):404-13. Epub 20140325. doi: 10.1107/S2053230X14003422. PubMed PMID: 24699729; PubMed Central PMCID: PMCPMC3976053.

6. Akdel M, Pires DEV, Pardo EP, Jänes J, Zalevsky AO, Mészáros B, et al. A structural biology community assessment of AlphaFold2 applications. Nat Struct Mol Biol. 2022;29(11):1056-67. Epub 20221107. doi: 10.1038/s41594-022-00849-w. PubMed PMID: 36344848; PubMed Central PMCID: PMCPMC9663297.
