# Supplementary material for: Assessment of oligomerization of bacterial micro-compartment shell components with the tripartite GFP reporter technology
Source: PLoS One. 2023 Nov 27;18(11):e0294760. doi: 10.1371/journal.pone.0294760 (PMC10681173; doi:10.1371/journal.pone.0294760)
Supplement: S3 File — (PDF) [file pone.0294760.s013.pdf]

Mounted figure 1C: Variation of protein expression depending on whether partners are coded in separated vectors or not.

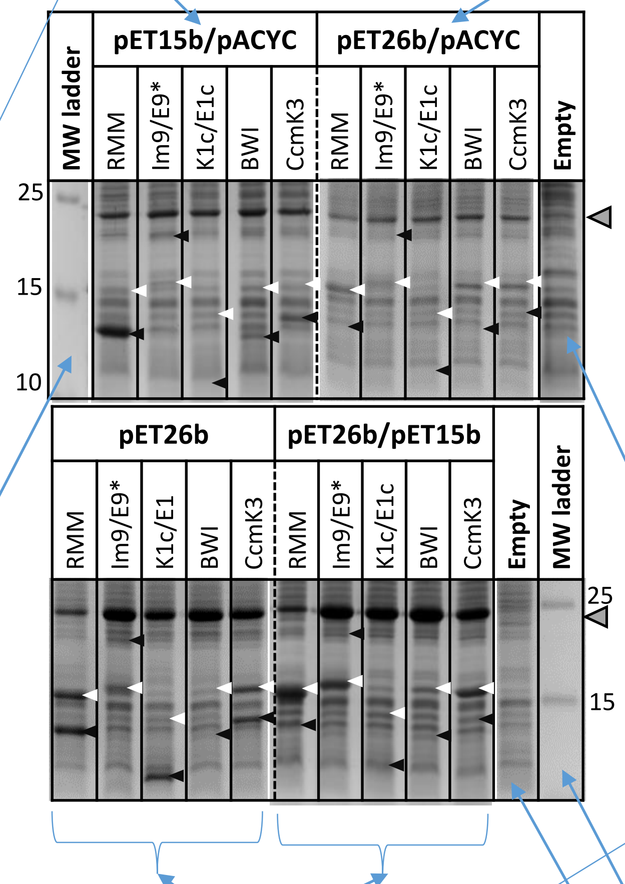

Original gels

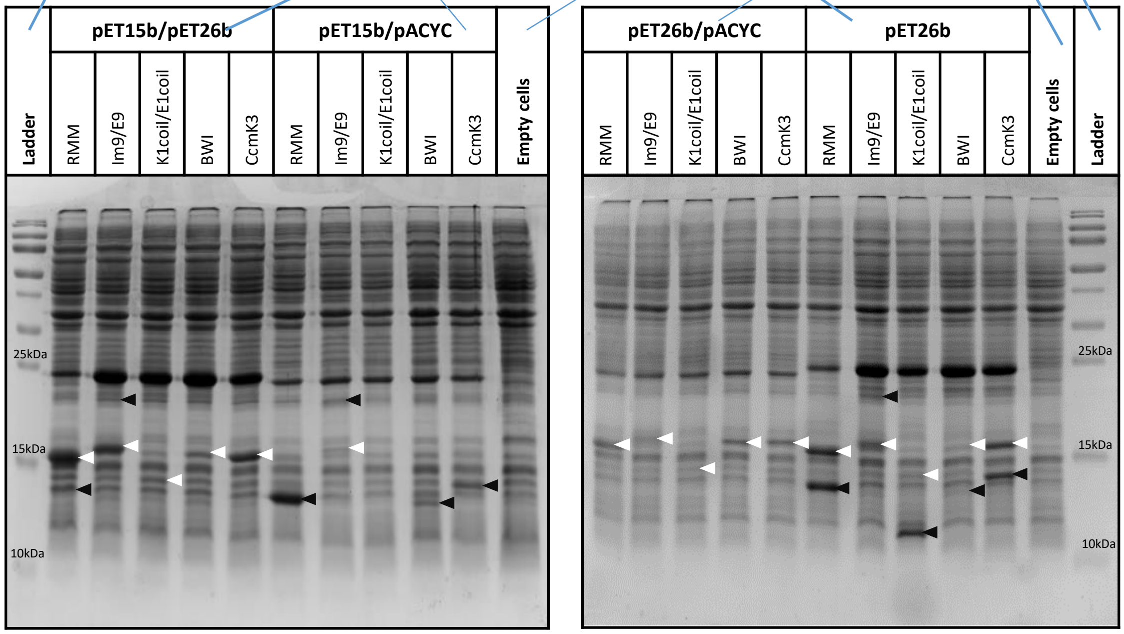

Mounted figure 4C: Kinetics of expression of RMM-10/11 and GFP1-9 depending on linker sizes.

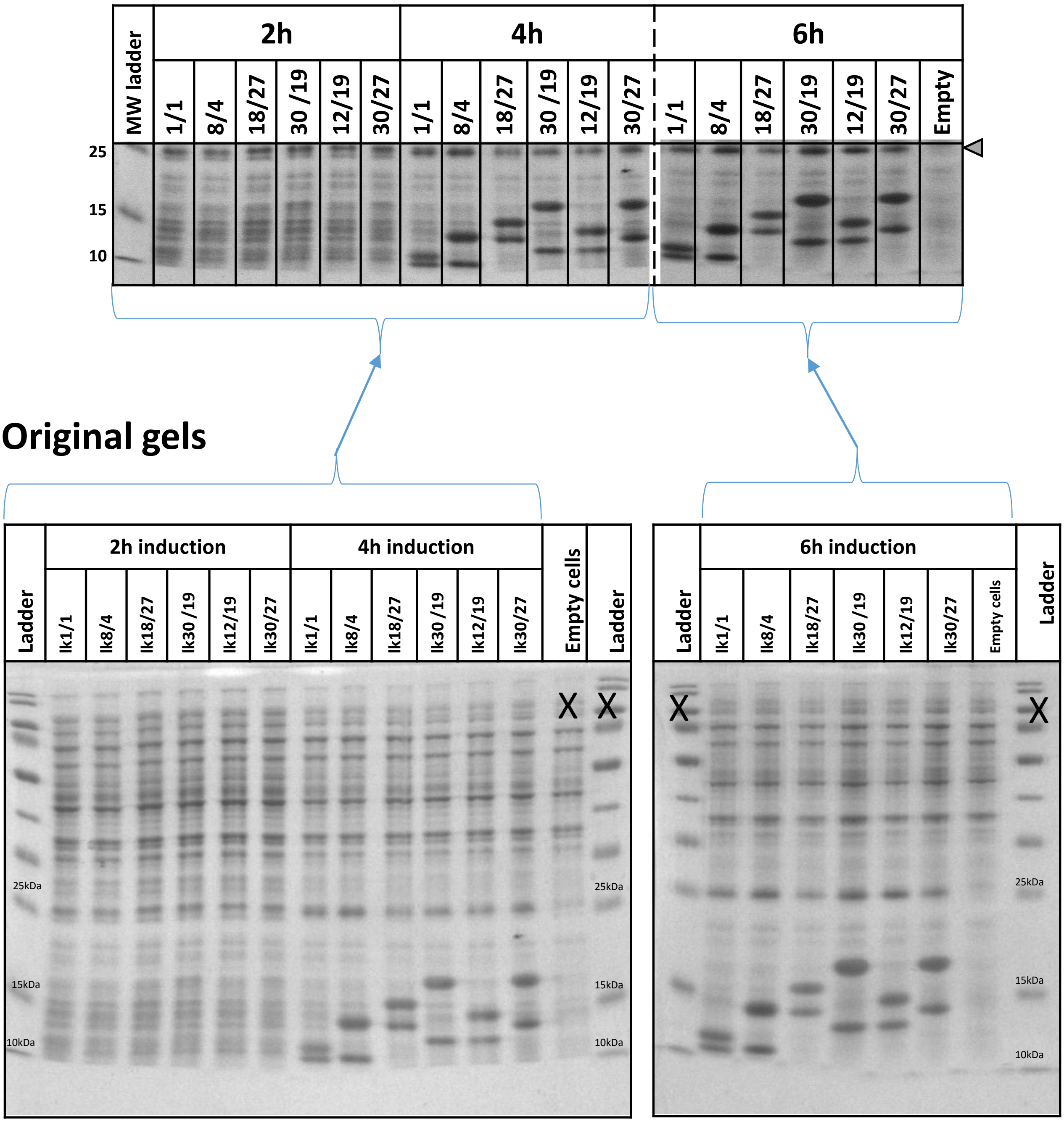

Mounted Figure 6B: Protein expression levels of SUMO-modified RMM (Histagged constructs)

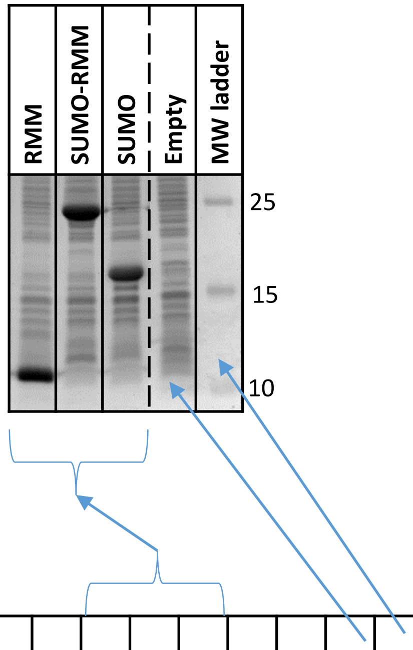

Original gel

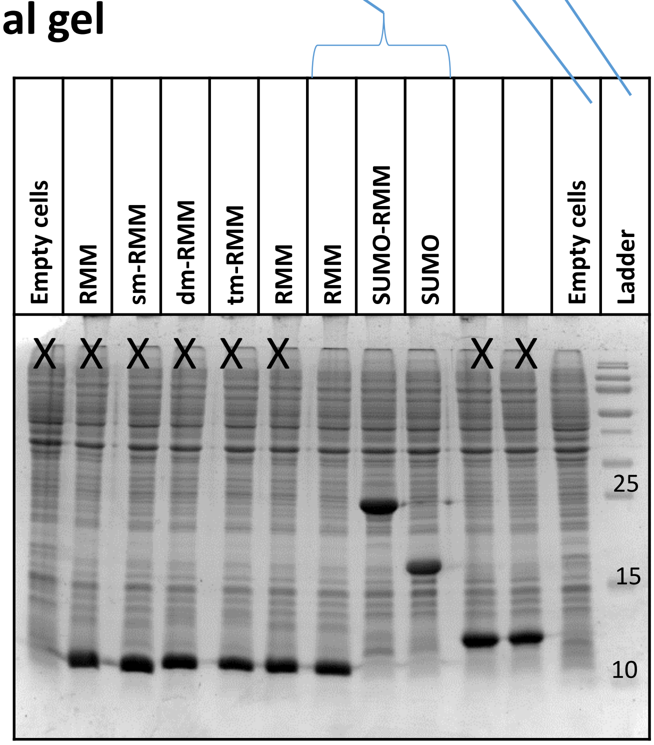

Mounted figure 6E: Protein expression of RMM fusions to SUMO and according to linker length

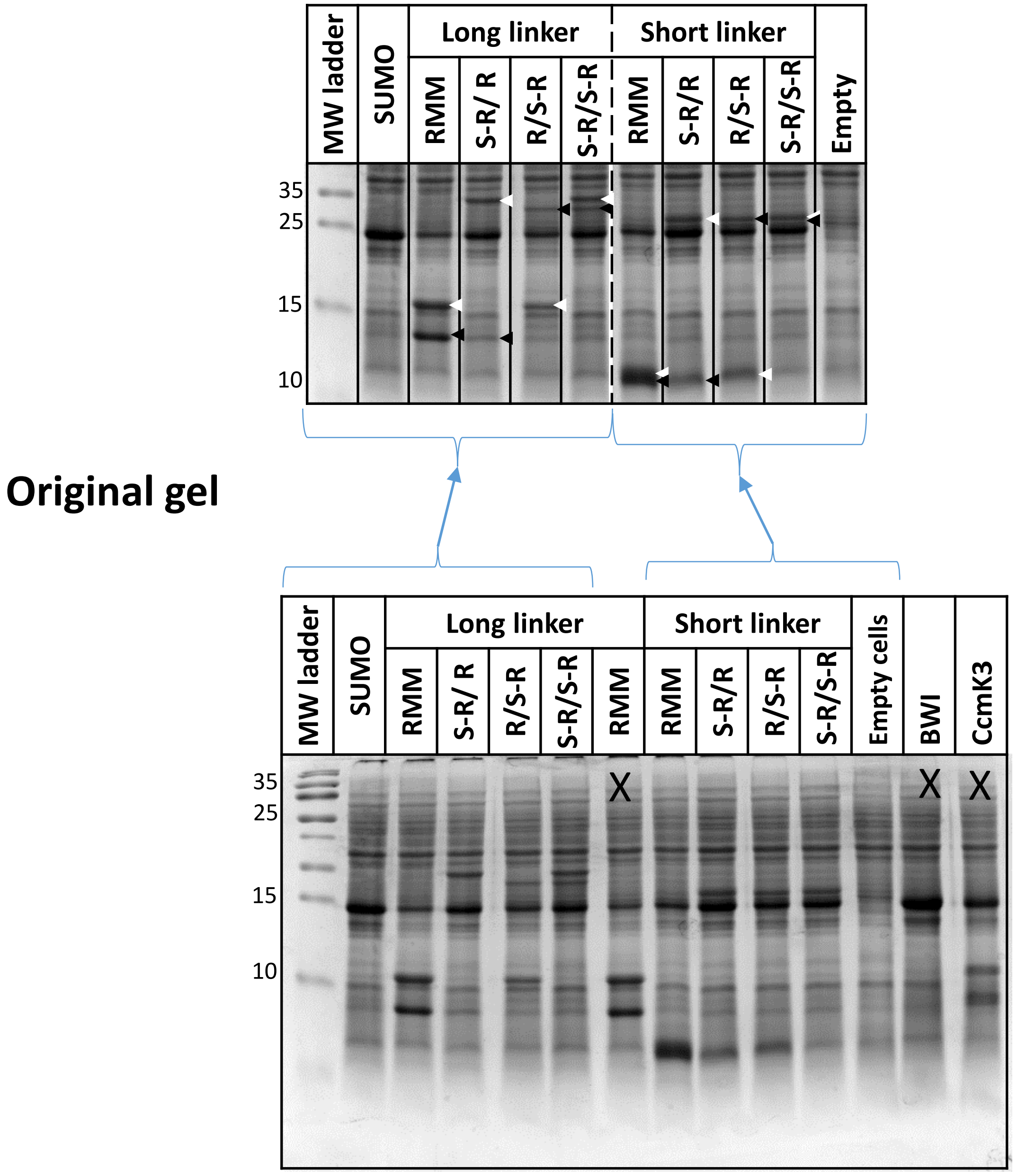

Mounted figure 7C: verification of expression of BMC-H, BMC-T and BMC-P shell components from different origins.

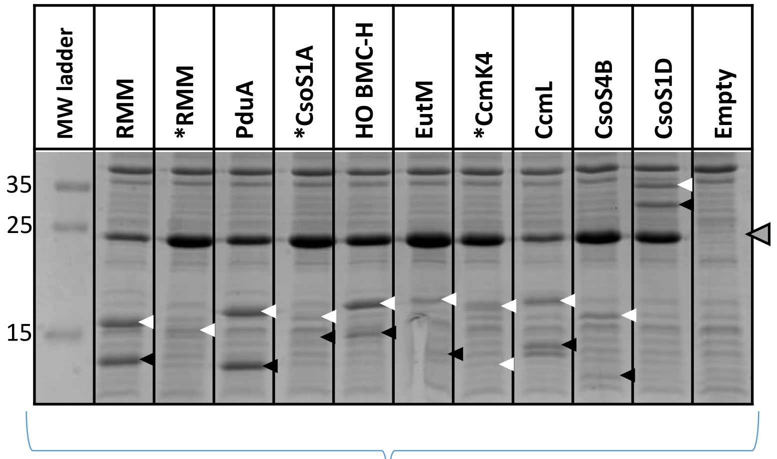

Original gel

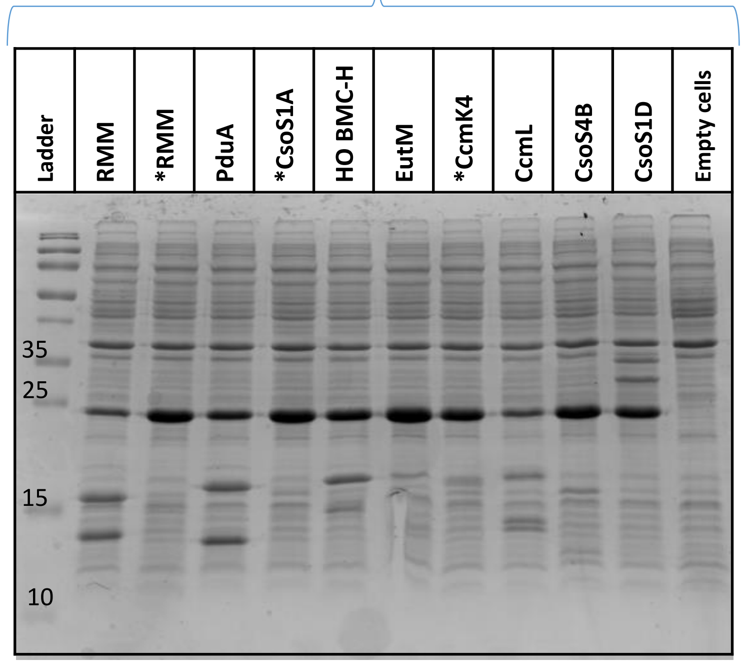

Mounted Figure S2B: Detection of POI-10 or POI-11 expression in the presence or absence of the GFP1-9

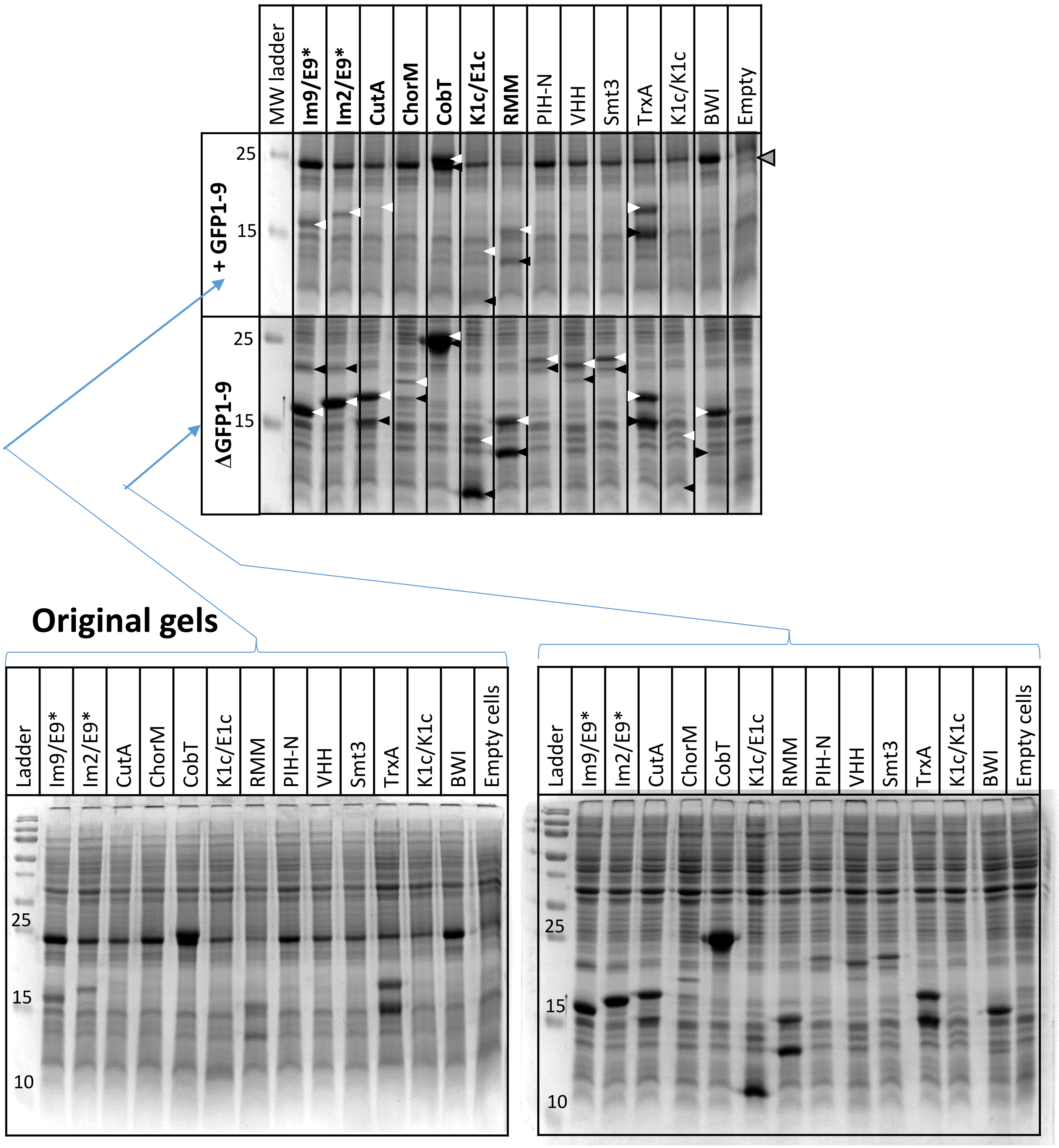

Mounted Figure S3: Diminished detection of protein bands when POI-10 or POI-11 are co-expressed with GFP1-9 in cells transformed with individual vectors (in the absence of the third tGFP partner).

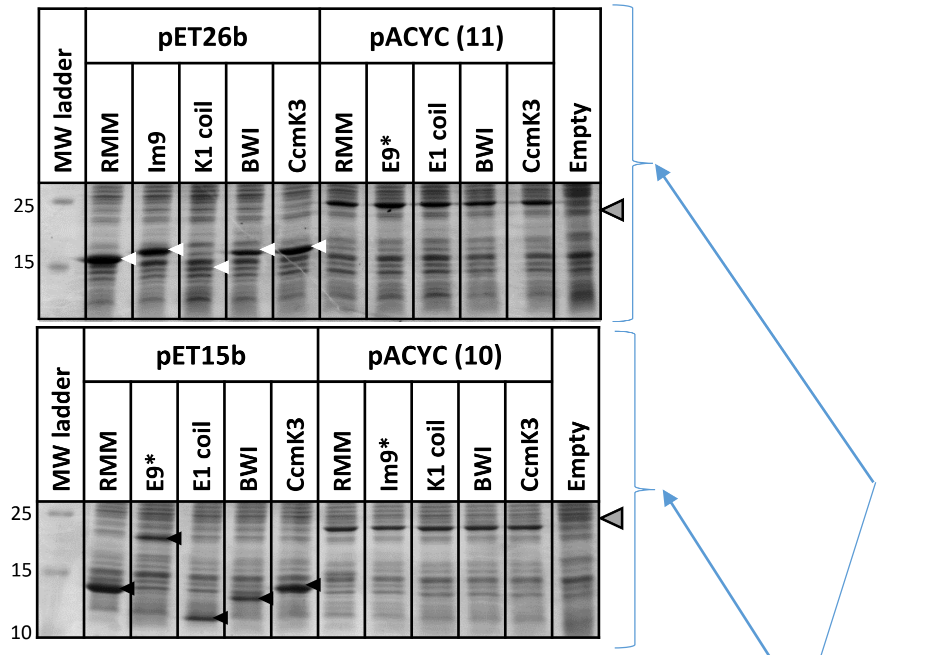

Original gels

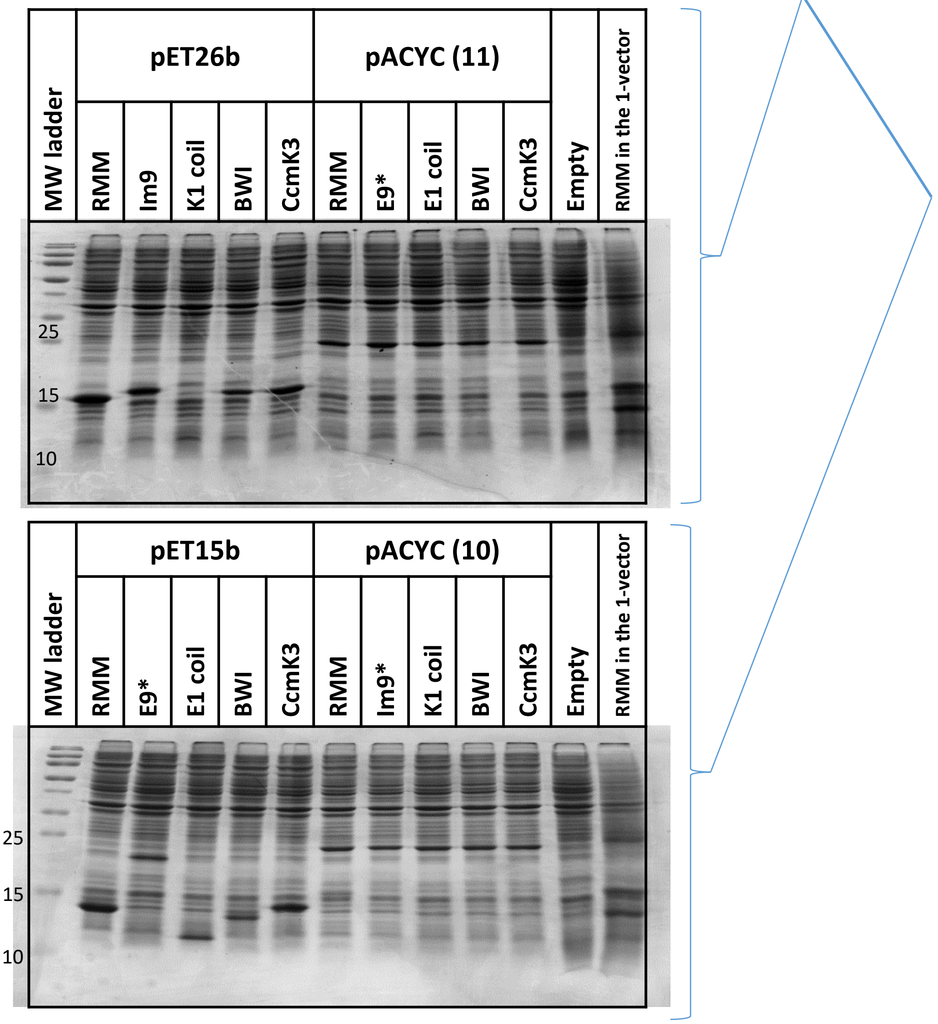

Mounted Figure S6E: SDS-PAGE gel to verify expression of RMM mutants, depending on type of tagging scheme [His6 or GFP10/11-tagged forms (tGFP)]

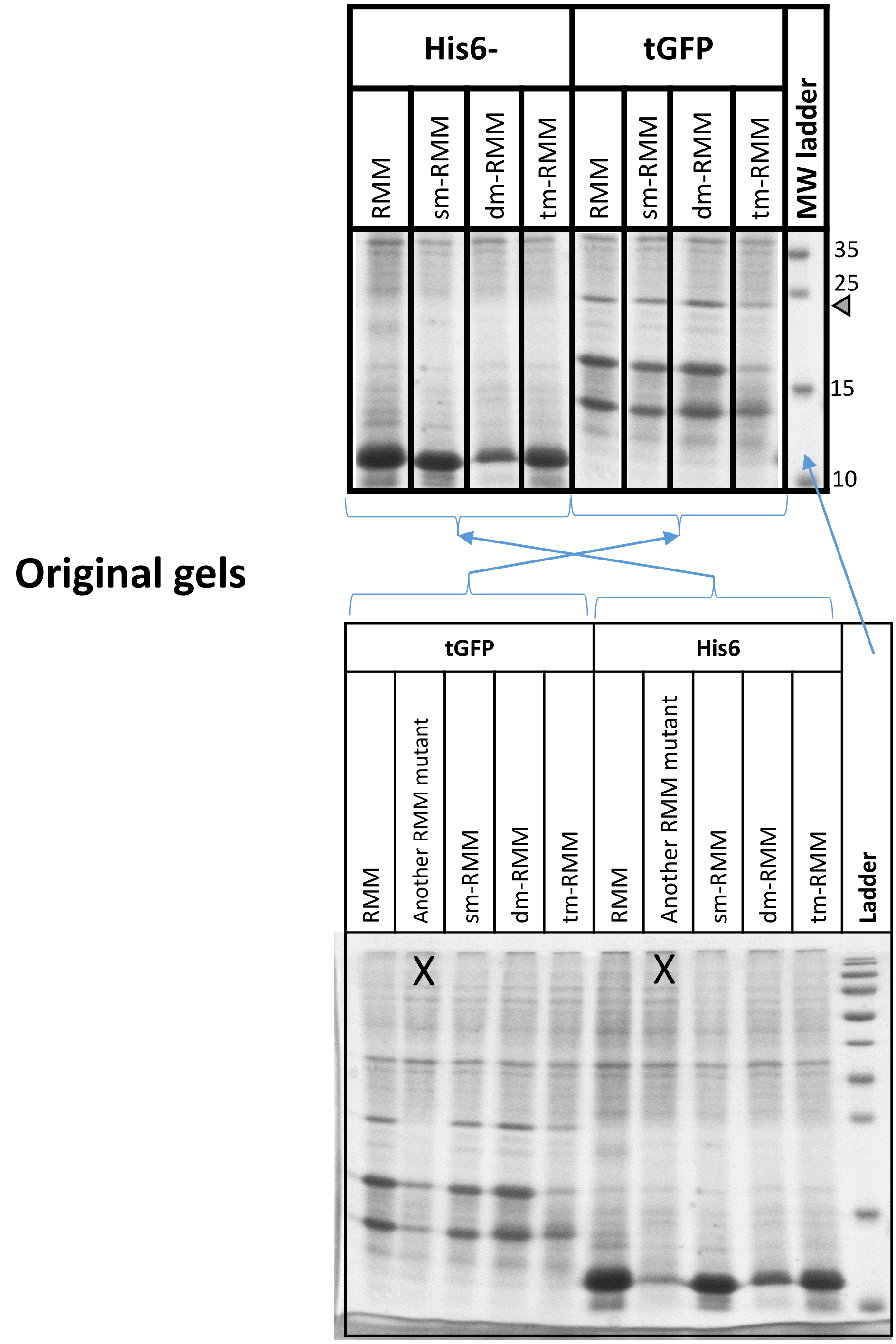

Figure S6E. SDS-PAGE gel to verify protein expression with RMM mutants according to tagging (His6 or GFP-tagged forms)
