# Supplementary material for: Assessment of oligomerization of bacterial micro-compartment shell components with the tripartite GFP reporter technology
Source: PLoS One. 2023 Nov 27;18(11):e0294760. doi: 10.1371/journal.pone.0294760 (PMC10681173; doi:10.1371/journal.pone.0294760)
Supplement: Attachment — Submitted filename: Response to reviewers.docx [file pone.0294760.s014.docx]

# Toulouse Biotechnology Institute

## UMR CNRS 5504 – UMR INRA 792

Toulouse, Wednesday, October 18, 2023

Please find below in black letters our reply to reviewers comments. Other minor changes were introduced, mainly with the intention to improve the discussion, which are mentioned throughout this letter and can also be easily visualized in the “Revised Manuscript with Track Changes” version.

Reviewer #1:

Just a few minor points/comments:
- For the shake of transparency, in Figs. 1B, 4C, 6B and E, 7C, S2B, S3 and S6E, the vertical lines drawn to delimit the boundaries of the tracks must be removed. The lettering at the top of each of them is sufficiently clear for their identification. At most, a small (e.g., 2-3 mm length) indentation line could be drawn to delimitate the wells, if desired. On the contrary, as the authors indicate, the dashed lines marking which pieces of the figure come from cropped gels seem appropriate.

We changed the mentioned figures to satisfy reviewer concerns.

- Page 6, line 124: “Technical replicates”. If, as stated, three distinct samples were explored, and not successive measurements of fluorescence reads from a single one, it rather should be termed “experimental replicates”.

In fact, as it is explained in more detail in the reply to Reviewer #2, this sentence was not correct. We now indicate that plotted data correspond to averages of independent repetitions of the experiment.

- P. 11, L. 241 and 243: “Apparition” should read better as “appearance”.

It has been modified. Thank you.

- P. 11, L. 265-268: This is a very insightful interpretation for the source of tGFP fluorescence in long vs. short linkers BMCs fusions: intramolecular vs. intermolecular assembly of pieces.

We thank the reviewer for the globaly positive commentaries.

Reviewer #2: 

In the materials and methods and figure legends, it is stated that reported fluorescence values correspond to fits of a sigmoidal model to the experimental data. I suggest including examples of raw data (with errors) and sigmoidal fits, at least in the supplementary information. Was the upper plateau of the sigmoidal curve retrieved from the fits the one represented in the graphs, after normalization?

Examples of such fluorescence raw data and of the fitting process are now included as supplementary figure S9. A comparative view of averaged raw data and best fit to the sigmoidal function is also depicted.

It is indicated that “data from a representative experiment are presented…. Averages and deviations could not be based on biological replicates, as absolute fluorescence values varied considerably among independent experiments” (Page 6). It is understandable that the fluorescence may vary between experiments. However, wouldn’t the performed normalization cancel these differences? In principle, normalized values should be consistent among biological replicates, supporting the robustness of the conclusions reached from the experiments. I strongly recommend showing averages of these normalized data and standard deviations, if possible. The error intervals would probably be wider than the ones currently shown, which are quite narrow, but they would better account for the intrinsic variability associated to the assays conducted.

The reviewer is right.

In fact, an updating error was introduced during the phases of preparation of the manuscript. For early versions of these figures, our intention was to present raw fluorescence values (averages from several independent experiments, each one including several clones for each case screened in the same microplate). But as mentioned in the manuscript, considerable variations of absolute signals occurred from experiment to experiment.

However, as reviewer says, normalized values did not differ as strongly among independent experiments. Several experiments could therefore be finally combined to calculate values plotted in the submitted figures. Unfortunately, we did not realize to update the sentence.

The sentence was replaced in the revised version of the manuscrip by "Data correspond to averages and standard deviations of at least two independent experiments, each one including several replicates per case (i.e. 3 clones), and are presented normalized because absolute fluorescence signals varied considerably among independent experiments*".*

.

Please, state how the reported errors of the fluorescence data were calculated. Do they correspond to the standard deviation?

Absolutely, they correspond to standard deviations. This is now explicitly mentioned in the figure legends and materials and methods.

I suggest revising the discussion and writing it more concisely, if possible.

In an attempt to be more concise, we removed a few sentences that were probably unnecessary in the discussion (lines 434-439 of the first submitted manuscript) and reorganized the neighbor paragraphs. We hope the reviewer will appreciate.

We were incapable of shrinking further the discussion without removing important considerations.

A reference to the relevant figure should be included in the paragraph corresponding to lines 258-263, page 12.

In fact, we did not plan to include any figure because the data are sufficiently simple as to be directly understood. In fact, such figure would consist of only two bars, reaching 100% and 15% levels.

We would prefer not to add such a figure to the long list of supplementary figures. But we would obviously do it if the reviewer insisted.

Are the inter-hexameric contacts between the BMC proteins considered artefactual? Could they be important to explain BMC assembly?

We are not sure to understand the questions.

There are two sources of inter-hexameric contacts: one deriving from random collisions of hexamers inside cells, the second one leading to higher-order BMC-H assembly (or BMC assembly when in the presence of other BMC components). The contribution to fluorescence signals of the first one is considered artefactual, as it does not inform on specific protein-protein interactions. The accumulation of such signals is likely a consequence of the irreversibility of GFP (and tGFP) reconstitution. On the other hand, contacts causing hexamer assembly are not considered artefactual. However, formation of these structures is supposed to start to occur above certain threshold concentrations.

**Enumeration of other changes introduced in the manuscript**:

Lines 87-89: we introduced a sentence and a reference to call the reader attention to the fact that GFP reconstitution is known to be an irreversible process (on classical split GFP schemes, not done for the tripartite), something that could contribute to the accumulation of fluorescence signals with negative PPI cases.

Lines 280-283: we added a measurement of nanotube size in longitudinal views, since we realized the values differed slightly from transversal views (estimations are more straightforward in transversal).

Lines 503-515: we reorganized this paragraph since we realized the arguments were not explained properly. We hope changes will help now to better follow the discussion on the possible reasons that could cause a decline of fluorescence with CsoS1D.
