# Supplementary material for: Assessment of oligomerization of bacterial micro-compartment shell components with the tripartite GFP reporter technology
Source: PLoS One. 2023 Nov 27;18(11):e0294760. doi: 10.1371/journal.pone.0294760 (PMC10681173; doi:10.1371/journal.pone.0294760)
Supplement: Peer review history

# Peer review: Decision Letter 0

**DOI:** 10.1371/journal.pone.0294760.r001

**Authors:** Maria Gasset

8 Oct 2023

PONE-D-23-29365Assessment of Oligomerization of Bacterial Micro-compartment Shell Components with the Tripartite GFP Reporter TechnologyPLOS ONE

Dear Dr. Garcia-Alles,

Thank you for submitting your manuscript to PLOS ONE, and personal apologies for the delay in getting back to you with the reeviwer´s comments. After careful consideration, we feel that your work needs several minor amendements before achieving PLOS ONE’s publication criteria . Therefore, we invite you to submit a revised version of the manuscript that addresses all the constructive points raised by both reviewers. Please submit your revised manuscript by Nov 22 2023 11:59PM. If you will need more time than this to complete your revisions, please reply to this message or contact the journal office at plosone@plos.org. When you're ready to submit your revision, log on to https://www.editorialmanager.com/pone/ and select the 'Submissions Needing Revision' folder to locate your manuscript file.

Please include the following items when submitting your revised manuscript:

- A rebuttal letter that responds to each point raised by the academic editor and reviewer(s). You should upload this letter as a separate file labeled 'Response to Reviewers'.
- A marked-up copy of your manuscript that highlights changes made to the original version. You should upload this as a separate file labeled 'Revised Manuscript with Track Changes'.
- An unmarked version of your revised paper without tracked changes. You should upload this as a separate file labeled 'Manuscript'.

If you would like to make changes to your financial disclosure, please include your updated statement in your cover letter. Guidelines for resubmitting your figure files are available below the reviewer comments at the end of this letter.

If applicable, we recommend that you deposit your laboratory protocols in protocols.io to enhance the reproducibility of your results. Protocols.io assigns your protocol its own identifier (DOI) so that it can be cited independently in the future. For instructions see: https://journals.plos.org/plosone/s/submission-guidelines#loc-laboratory-protocols. Additionally, PLOS ONE offers an option for publishing peer-reviewed Lab Protocol articles, which describe protocols hosted on protocols.io. Read more information on sharing protocols at https://plos.org/protocols?utm_medium=editorial-email&utm_source=authorletters&utm_campaign=protocols.

We look forward to receiving your revised manuscript.

Kind regards,

Maria Gasset, Ph.D.

Academic Editor

PLOS ONE

Journal Requirements:

When submitting your revision, we need you to address these additional requirements.

1. Please ensure that your manuscript meets PLOS ONE's style requirements, including those for file naming. The PLOS ONE style templates can be found at 

https://journals.plos.org/plosone/s/file?id=wjVg/PLOSOne_formatting_sample_main_body.pdf and 

https://journals.plos.org/plosone/s/file?id=ba62/PLOSOne_formatting_sample_title_authors_affiliations.pdf

2. Please note that PLOS ONE has specific guidelines on code sharing for submissions in which author-generated code underpins the findings in the manuscript. In these cases, all author-generated code must be made available without restrictions upon publication of the work. Please review our guidelines at https://journals.plos.org/plosone/s/materials-and-software-sharing#loc-sharing-code and ensure that your code is shared in a way that follows best practice and facilitates reproducibility and reuse.

3. Thank you for stating the following in the Acknowledgments Section of your manuscript: "The French National Research Agency (ANR) supported financially this work: ANR-19-CE09-0032-01. This work also benefited from a grant managed by the same agency, under the "Investissements d'Avenir" programme: ANR-18-EURE-0021. We thank Denis Jallet and Philippe Urban, both based at TBI institute, for careful reading and advices during the late phases of manuscript preparation."

We note that you have provided funding information that is not currently declared in your Funding Statement. However, funding information should not appear in the Acknowledgments section or other areas of your manuscript. We will only publish funding information present in the Funding Statement section of the online submission form. 

Please remove any funding-related text from the manuscript and let us know how you would like to update your Funding Statement. Currently, your Funding Statement reads as follows: "The French National Research Agency (ANR) supported financially this work: ANR-19-CE09-0032-01. This work also benefited from a grant managed by the same agency, under the "Investissements d'Avenir" programme: ANR-18-EURE-0021. The funders had no role in study design, data collection and analysis, decision to publish, or preparation of the manuscript."

Please include your amended statements within your cover letter; we will change the online submission form on your behalf.

4. PLOS ONE now requires that authors provide the original uncropped and unadjusted images underlying all blot or gel results reported in a submission’s figures or Supporting Information files. This policy and the journal’s other requirements for blot/gel reporting and figure preparation are described in detail at https://journals.plos.org/plosone/s/figures#loc-blot-and-gel-reporting-requirements and https://journals.plos.org/plosone/s/figures#loc-preparing-figures-from-image-files. When you submit your revised manuscript, please ensure that your figures adhere fully to these guidelines and provide the original underlying images for all blot or gel data reported in your submission. See the following link for instructions on providing the original image data: https://journals.plos.org/plosone/s/figures#loc-original-images-for-blots-and-gels. 

  

In your cover letter, please note whether your blot/gel image data are in Supporting Information or posted at a public data repository, provide the repository URL if relevant, and provide specific details as to which raw blot/gel images, if any, are not available. Email us at plosone@plos.org if you have any questions.

5. Please review your reference list to ensure that it is complete and correct. If you have cited papers that have been retracted, please include the rationale for doing so in the manuscript text, or remove these references and replace them with relevant current references. Any changes to the reference list should be mentioned in the rebuttal letter that accompanies your revised manuscript. If you need to cite a retracted article, indicate the article’s retracted status in the References list and also include a citation and full reference for the retraction notice.

[Note: HTML markup is below. Please do not edit.]

Reviewers' comments:

Reviewer's Responses to Questions

**Comments to the Author**

1. Is the manuscript technically sound, and do the data support the conclusions?

The manuscript must describe a technically sound piece of scientific research with data that supports the conclusions. Experiments must have been conducted rigorously, with appropriate controls, replication, and sample sizes. The conclusions must be drawn appropriately based on the data presented. 

Reviewer #1: Yes

Reviewer #2: Yes

**********

2. Has the statistical analysis been performed appropriately and rigorously? 

Reviewer #1: N/A

Reviewer #2: Yes

**********

3. Have the authors made all data underlying the findings in their manuscript fully available?

The PLOS Data policy requires authors to make all data underlying the findings described in their manuscript fully available without restriction, with rare exception (please refer to the Data Availability Statement in the manuscript PDF file). The data should be provided as part of the manuscript or its supporting information, or deposited to a public repository. For example, in addition to summary statistics, the data points behind means, medians and variance measures should be available. If there are restrictions on publicly sharing data—e.g. participant privacy or use of data from a third party—those must be specified.

Reviewer #1: Yes

Reviewer #2: Yes

**********

4. Is the manuscript presented in an intelligible fashion and written in standard English?

PLOS ONE does not copyedit accepted manuscripts, so the language in submitted articles must be clear, correct, and unambiguous. Any typographical or grammatical errors should be corrected at revision, so please note any specific errors here.

Reviewer #1: Yes

Reviewer #2: Yes

**********

5. Review Comments to the Author

Please use the space provided to explain your answers to the questions above. You may also include additional comments for the author, including concerns about dual publication, research ethics, or publication ethics. (Please upload your review as an attachment if it exceeds 20,000 characters)

Reviewer #1: This manuscript by Barthe et al. is a complete tour-de force to develop a screening method for exploring the formation of heterologous assemblies by distinct members of the protein shells forming micro-compartments in bacteria (BMCs). These constitute a hot topic in SynBio and protein engineering.

The authors build on the known technology of strand complementation in split variants of the green fluorescent protein (tGFP) in which a core formed by the antiparallel strands 1-9 is complemented with the two missing ones (#10 and 11), independently cloned and expressed as fusions to any protein of interest (in this case, RMM from Mycobacterium, CcmK3 from Synechocystis, and control proteins such as bacteriocin E9 and its immunity protein or the BWI protease inhibitor). Quite often, this technology suffers from aggregation of the fusions, failing to reconstruct a complete functional, fluorescence-emitting GFP barrel (the reviewer’s group tried once with the split GFP 1-10 + GFP11 variant of the assay, with no success, so we know well how difficult it may be!). To overcome this potential flaws, Barthe et al. carried out an exhaustive study to explore the suitability of distinct vector platforms: independent replicons, different promoters in a single DNA molecule, or alternative combinations of operon arrangements (the option favoured). In addition, it was also tested the optimal range of linker lengths (for 1 to 30 amino acid residues) between the BMC protein of interest and the fused GFP 10/11 stands or the GFP 1-9 core, with the finding that longer linkers usually provide more efficient fluorescence emission. Fusions to a bulky soluble domain (SUMO) and a number of mutations predicted by AlphaFold2 to perturb the oligomerization interface(s) in BMCs were also tested as the involved proteins naturally forms hexamers (H), trimers (T) or pentamers (P) at the shells. Finally, electron microscopy was used to study the formation of bundles or clusters of BMCs shells, or their disappearance, for the distinct constructs within the E. coli cells.

Such comprehensive approach allowed Barthe et al. to extract some valid conclusions on the ability of some of the BMC-H/T/P protein variants, among nine different proteins from bacterial micro-compartments tested, to establish oligomeric complexes. Overall, this is a solid scientific work that may constitute a methodological advance for the characterization of the association state in protein complexes.

Just a few minor points/comments:

- For the shake of transparency, in Figs. 1B, 4C, 6B and E, 7C, S2B, S3 and S6E, the vertical lines drawn to delimit the boundaries of the tracks must be removed. The lettering at the top of each of them is sufficiently clear for their identification. At most, a small (e.g., 2-3 mm length) indentation line could be drawn to delimitate the wells, if desired. On the contrary, as the authors indicate, the dashed lines marking which pieces of the figure come from cropped gels seem appropriate.

- Page 6, line 124: “Technical replicates”. If, as stated, three distinct samples were explored, and not successive measurements of fluorescence reads from a single one, it rather should be termed “experimental replicates”.

- P. 11, L. 241 and 243: “Apparition” should read better as “appearance”.

- P. 11, L. 265-268: This is a very insightful interpretation for the source of tGFP fluorescence in long vs. short linkers BMCs fusions: intramolecular vs. intermolecular assembly of pieces.

Reviewer #2: In the current article, the performance of the tripartite GFP reporter technology for the detection of homo and heteroassociations of proteins from the shell of bacterial Micro-compartments is evaluated. Aspects like the length of the linker between the probed proteins and the GFP fragments, the characteristics of the promoters, the genetic organization and the number of vectors used in the assay are tested and optimized. The detection of hetero-oligomerizations of BMC proteins using the optimized tGFP procedure is validated by comparison with previously reported data obtained by Western blot, finding a good agreement. Limitations of the technique are also stated and discussed, principally related with the solubility of the peptides.

This is a carefully conducted study addressing a relevant aspect for protein function, the formation of oligomers and heterocomplexes. The analysis of protein-protein interactions is of paramount importance for deciphering the molecular mechanisms underlying biological processes and it is particularly challenging. The proteins chosen for this study play important roles for the still enigmatic, yet most relevant assembly of bacterial microcompartments, key for the organization of the intracellular space in these cells, which lack the typical membrane-bound compartments of eukaryotic cytoplasms. Approaches such as the one proposed here would be of help to improve our current understanding of BMC assembly. The article is well written and organized. Suitable controls are thoughtfully designed at the beginning of the study and kept across the experiments.

Specific comments:

In the materials and methods and figure legends, it is stated that reported fluorescence values correspond to fits of a sigmoidal model to the experimental data. I suggest including examples of raw data (with errors) and sigmoidal fits, at least in the supplementary information. Was the upper plateau of the sigmoidal curve retrieved from the fits the one represented in the graphs, after normalization?

It is indicated that “data from a representative experiment are presented…. Averages and deviations could not be based on biological replicates, as absolute fluorescence values varied considerably among independent experiments” (Page 6). It is understandable that the fluorescence may vary between experiments. However, wouldn’t the performed normalization cancel these differences? In principle, normalized values should be consistent among biological replicates, supporting the robustness of the conclusions reached from the experiments. I strongly recommend showing averages of these normalized data and standard deviations, if possible. The error intervals would probably be wider than the ones currently shown, which are quite narrow, but they would better account for the intrinsic variability associated to the assays conducted.

Please, state how the reported errors of the fluorescence data were calculated. Do they correspond to the standard deviation?

I suggest revising the discussion and writing it more concisely, if possible.

A reference to the relevant figure should be included in the paragraph corresponding to lines 258-263, page 12.

Are the inter-hexameric contacts between the BMC proteins considered artefactual? Could they be important to explain BMC assembly?

**********

6. PLOS authors have the option to publish the peer review history of their article (what does this mean?). If published, this will include your full peer review and any attached files.

If you choose “no”, your identity will remain anonymous but your review may still be made public.

**Do you want your identity to be public for this peer review?** For information about this choice, including consent withdrawal, please see our Privacy Policy.

Reviewer #1: No

Reviewer #2: No

**********

[NOTE: If reviewer comments were submitted as an attachment file, they will be attached to this email and accessible via the submission site. Please log into your account, locate the manuscript record, and check for the action link "View Attachments". If this link does not appear, there are no attachment files.]

While revising your submission, please upload your figure files to the Preflight Analysis and Conversion Engine (PACE) digital diagnostic tool, https://pacev2.apexcovantage.com/. PACE helps ensure that figures meet PLOS requirements. To use PACE, you must first register as a user. Registration is free. Then, login and navigate to the UPLOAD tab, where you will find detailed instructions on how to use the tool. If you encounter any issues or have any questions when using PACE, please email PLOS at figures@plos.org. Please note that Supporting Information files do not need this step.

# Author comment: Author response to Decision Letter 0

**DOI:** 10.1371/journal.pone.0294760.r002

19 Oct 2023

Reviewer #1: 

Just a few minor points/comments:

- For the shake of transparency, in Figs. 1B, 4C, 6B and E, 7C, S2B, S3 and S6E, the vertical lines drawn to delimit the boundaries of the tracks must be removed. The lettering at the top of each of them is sufficiently clear for their identification. At most, a small (e.g., 2-3 mm length) indentation line could be drawn to delimitate the wells, if desired. On the contrary, as the authors indicate, the dashed lines marking which pieces of the figure come from cropped gels seem appropriate.

We changed the mentioned figures to satisfy reviewer concerns.

- Page 6, line 124: “Technical replicates”. If, as stated, three distinct samples were explored, and not successive measurements of fluorescence reads from a single one, it rather should be termed “experimental replicates”.

In fact, as it is explained in more detail in the reply to Reviewer #2, this sentence was not correct. We now indicate that plotted data correspond to averages of independent repetitions of the experiment.

- P. 11, L. 241 and 243: “Apparition” should read better as “appearance”.

It has been modified. Thank you.

- P. 11, L. 265-268: This is a very insightful interpretation for the source of tGFP fluorescence in long vs. short linkers BMCs fusions: intramolecular vs. intermolecular assembly of pieces.

We thank the reviewer for the globaly positive commentaries.

Reviewer #2: 

In the materials and methods and figure legends, it is stated that reported fluorescence values correspond to fits of a sigmoidal model to the experimental data. I suggest including examples of raw data (with errors) and sigmoidal fits, at least in the supplementary information. Was the upper plateau of the sigmoidal curve retrieved from the fits the one represented in the graphs, after normalization?

Examples of such fluorescence raw data and of the fitting process are now included as supplementary figure S9. A comparative view of averaged raw data and best fit to the sigmoidal function is also depicted.

It is indicated that “data from a representative experiment are presented…. Averages and deviations could not be based on biological replicates, as absolute fluorescence values varied considerably among independent experiments” (Page 6). It is understandable that the fluorescence may vary between experiments. However, wouldn’t the performed normalization cancel these differences? In principle, normalized values should be consistent among biological replicates, supporting the robustness of the conclusions reached from the experiments. I strongly recommend showing averages of these normalized data and standard deviations, if possible. The error intervals would probably be wider than the ones currently shown, which are quite narrow, but they would better account for the intrinsic variability associated to the assays conducted.

The reviewer is right. 

In fact, an updating error was introduced during the phases of preparation of the manuscript. For early versions of these figures, our intention was to present raw fluorescence values (averages from several independent experiments, each one including several clones for each case screened in the same microplate). But as mentioned in the manuscript, considerable variations of absolute signals occurred from experiment to experiment. 

However, as reviewer says, normalized values did not differ as strongly among independent experiments. Several experiments could therefore be finally combined to calculate values plotted in the submitted figures. Unfortunately, we did not realize to update the sentence.

The sentence was replaced in the revised version of the manuscrip by "Data correspond to averages and standard deviations of at least two independent experiments, each one including several replicates per case (i.e. 3 clones), and are presented normalized because absolute fluorescence signals varied considerably among independent experiments".

.

Please, state how the reported errors of the fluorescence data were calculated. Do they correspond to the standard deviation?

Absolutely, they correspond to standard deviations. This is now explicitly mentioned in the figure legends and materials and methods. 

I suggest revising the discussion and writing it more concisely, if possible.

In an attempt to be more concise, we removed a few sentences that were probably unnecessary in the discussion (lines 434-439 of the first submitted manuscript) and reorganized the neighbor paragraphs. We hope the reviewer will appreciate.

We were incapable of shrinking further the discussion without removing important considerations.

A reference to the relevant figure should be included in the paragraph corresponding to lines 258-263, page 12.

In fact, we did not plan to include any figure because the data are sufficiently simple as to be directly understood. In fact, such figure would consist of only two bars, reaching 100% and 15% levels. 

We would prefer not to add such a figure to the long list of supplementary figures. But we would obviously do it if the reviewer insisted.

Are the inter-hexameric contacts between the BMC proteins considered artefactual? Could they be important to explain BMC assembly?

We are not sure to understand the questions. 

There are two sources of inter-hexameric contacts: one deriving from random collisions of hexamers inside cells, the second one leading to higher-order BMC-H assembly (or BMC assembly when in the presence of other BMC components). The contribution to fluorescence signals of the first one is considered artefactual, as it does not inform on specific protein-protein interactions. The accumulation of such signals is likely a consequence of the irreversibility of GFP (and tGFP) reconstitution. On the other hand, contacts causing hexamer assembly are not considered artefactual. However, formation of these structures is supposed to start to occur above certain threshold concentrations.

Attachment Submitted filename: Response to reviewers.docx Click here for additional data file.

# Peer review: Decision Letter 1

**DOI:** 10.1371/journal.pone.0294760.r003

**Authors:** Maria Gasset

8 Nov 2023

Assessment of Oligomerization of Bacterial Micro-compartment Shell Components with the Tripartite GFP Reporter Technology

PONE-D-23-29365R1

Dear Dr. Luis F. García-Alles,

We’re pleased to inform you that your manuscript has been judged scientifically suitable for publication and will be formally accepted for publication once it meets all outstanding technical requirements.

Within one week, you’ll receive an e-mail detailing the required amendments. When these have been addressed, you’ll receive a formal acceptance letter and your manuscript will be scheduled for publication.

An invoice for payment will follow shortly after the formal acceptance. To ensure an efficient process, please log into Editorial Manager at http://www.editorialmanager.com/pone/, click the 'Update My Information' link at the top of the page, and double check that your user information is up-to-date. If you have any billing related questions, please contact our Author Billing department directly at authorbilling@plos.org.

If your institution or institutions have a press office, please notify them about your upcoming paper to help maximize its impact. If they’ll be preparing press materials, please inform our press team as soon as possible -- no later than 48 hours after receiving the formal acceptance. Your manuscript will remain under strict press embargo until 2 pm Eastern Time on the date of publication. For more information, please contact onepress@plos.org.

Kind regards,

Maria Gasset, Ph.D.

Academic Editor

PLOS ONE

Additional Editor Comments (optional):

Reviewers' comments:

Reviewer's Responses to Questions

**Comments to the Author**

1. If the authors have adequately addressed your comments raised in a previous round of review and you feel that this manuscript is now acceptable for publication, you may indicate that here to bypass the “Comments to the Author” section, enter your conflict of interest statement in the “Confidential to Editor” section, and submit your "Accept" recommendation.

Reviewer #1: All comments have been addressed

Reviewer #2: All comments have been addressed

**********

2. Is the manuscript technically sound, and do the data support the conclusions?

The manuscript must describe a technically sound piece of scientific research with data that supports the conclusions. Experiments must have been conducted rigorously, with appropriate controls, replication, and sample sizes. The conclusions must be drawn appropriately based on the data presented. 

Reviewer #1: Yes

Reviewer #2: (No Response)

**********

3. Has the statistical analysis been performed appropriately and rigorously? 

Reviewer #1: N/A

Reviewer #2: (No Response)

**********

4. Have the authors made all data underlying the findings in their manuscript fully available?

The PLOS Data policy requires authors to make all data underlying the findings described in their manuscript fully available without restriction, with rare exception (please refer to the Data Availability Statement in the manuscript PDF file). The data should be provided as part of the manuscript or its supporting information, or deposited to a public repository. For example, in addition to summary statistics, the data points behind means, medians and variance measures should be available. If there are restrictions on publicly sharing data—e.g. participant privacy or use of data from a third party—those must be specified.

Reviewer #1: Yes

Reviewer #2: (No Response)

**********

5. Is the manuscript presented in an intelligible fashion and written in standard English?

PLOS ONE does not copyedit accepted manuscripts, so the language in submitted articles must be clear, correct, and unambiguous. Any typographical or grammatical errors should be corrected at revision, so please note any specific errors here.

Reviewer #1: Yes

Reviewer #2: (No Response)

**********

6. Review Comments to the Author

Please use the space provided to explain your answers to the questions above. You may also include additional comments for the author, including concerns about dual publication, research ethics, or publication ethics. (Please upload your review as an attachment if it exceeds 20,000 characters)

Reviewer #1: (No Response)

Reviewer #2: (No Response)

**********

7. PLOS authors have the option to publish the peer review history of their article (what does this mean?). If published, this will include your full peer review and any attached files.

If you choose “no”, your identity will remain anonymous but your review may still be made public.

**Do you want your identity to be public for this peer review?** For information about this choice, including consent withdrawal, please see our Privacy Policy.

Reviewer #1: No

Reviewer #2: No

**********

# Editor report: Acceptance letter

**DOI:** 10.1371/journal.pone.0294760.r004

**Authors:** Maria Gasset

14 Nov 2023

PONE-D-23-29365R1 

Assessment of Oligomerization of Bacterial Micro-compartment Shell Components with the Tripartite GFP Reporter Technology 

Dear Dr. Garcia-Alles:

I'm pleased to inform you that your manuscript has been deemed suitable for publication in PLOS ONE. Congratulations! Your manuscript is now with our production department. 

If your institution or institutions have a press office, please let them know about your upcoming paper now to help maximize its impact. If they'll be preparing press materials, please inform our press team within the next 48 hours. Your manuscript will remain under strict press embargo until 2 pm Eastern Time on the date of publication. For more information please contact onepress@plos.org.

If we can help with anything else, please email us at customercare@plos.org.

Thank you for submitting your work to PLOS ONE and supporting open access. 

Kind regards, 

PLOS ONE Editorial Office Staff

on behalf of

Dr. Maria Gasset 

Academic Editor

PLOS ONE
